# The InBIO Barcoding Initiative Database: DNA barcodes of Iberian Bees

**DOI:** 10.3897/BDJ.12.e117172

**Published:** 2024-03-05

**Authors:** Thomas James Wood, Hugo Gaspar, Romain Le Divelec, Andreia Penado, Teresa Luísa Silva, Vanessa A. Mata, Joana Veríssimo, Denis Michez, Sílvia Castro, João Loureiro, Pedro Beja, Sónia Ferreira

**Affiliations:** 1 Naturalis Biodiversity Center, Leiden, Netherlands Naturalis Biodiversity Center Leiden Netherlands; 2 Centre for Functional Ecology, Associate Laboratory TERRA, Department of Life Sciences, University of Coimbra, Calçada Martim de Freitas, 3000-456, Coimbra, Portugal Centre for Functional Ecology, Associate Laboratory TERRA, Department of Life Sciences, University of Coimbra, Calçada Martim de Freitas, 3000-456 Coimbra Portugal; 3 University of Mons, Research Institute for Biosciences, Laboratory of Zoology, Place du parc 20, 7000, Mons, Belgium University of Mons, Research Institute for Biosciences, Laboratory of Zoology, Place du parc 20, 7000 Mons Belgium; 4 Ciência Viva - Agência Nacional para a Cultura Científica e Tecnológica, Largo José Mariano Gago n.º1, Parque das Nações, 1990-073, Lisboa, Portugal Ciência Viva - Agência Nacional para a Cultura Científica e Tecnológica, Largo José Mariano Gago n.º1, Parque das Nações, 1990-073 Lisboa Portugal; 5 BIOPOLIS Program in Genomics, Biodiversity and Land Planning, CIBIO, Campus de Vairão, 4485–661 Vairão, Vila do Conde, Portugal BIOPOLIS Program in Genomics, Biodiversity and Land Planning, CIBIO, Campus de Vairão, 4485–661 Vairão Vila do Conde Portugal; 6 CIBIO, Centro de Investigação em Biodiversidade e Recursos Genéticos, InBIO Laboratório Associado, Campus de Vairão, Universidade do Porto, 4485-661 Vairão, Vila do Conde, Portugal CIBIO, Centro de Investigação em Biodiversidade e Recursos Genéticos, InBIO Laboratório Associado, Campus de Vairão, Universidade do Porto, 4485-661 Vairão Vila do Conde Portugal; 7 University of Mons, Bruxelles, Belgium University of Mons Bruxelles Belgium; 8 EBM, Estação Biológica de Mertola, Praça Luis de Camoes, Mertola, Mertola, Portugal EBM, Estação Biológica de Mertola, Praça Luis de Camoes, Mertola Mertola Portugal

**Keywords:** Hymenoptera, occurrence records, species distributions, peninsular Portugal, peninsular Spain, DNA barcode, cytochrome c oxidase subunit I (COI), pollinator

## Abstract

**Background:**

Bees are important actors in terrestrial ecosystems and are recognised for their prominent role as pollinators. In the Iberian Peninsula, approximately 1,100 bee species are known, with nearly 100 of these species being endemic to the Peninsula. A reference collection of DNA barcodes, based on morphologically identified bee specimens, representing 514 Iberian species, was constructed. The "InBIO Barcoding Initiative Database: DNA Barcodes of Iberian bees" dataset contains records of 1,059 sequenced specimens. The species of this dataset correspond to about 47% of Iberian bee species diversity and 21% of endemic species diversity. For peninsular Portugal only, the corresponding coverage is 71% and 50%. Specimens were collected between 2014 and 2022 and are deposited in the research collection of Thomas Wood (Naturalis Biodiversity Center, The Netherlands), in the FLOWer Lab collection at the University of Coimbra (Portugal), in the Andreia Penado collection at the Natural History and Science Museum of the University of Porto (MHNC-UP) (Portugal) and in the InBIO Barcoding Initiative (IBI) reference collection (Vairão, Portugal).

**New information:**

Of the 514 species sequenced, 75 species from five different families are new additions to the Barcode of Life Data System (BOLD) and 112 new BINs were added. Whilst the majority of species were assigned to a single BIN (94.9%), 27 nominal species were assigned to multiple BINs. Although the placement into multiple BINs may simply reflect genetic diversity and variation, it likely also represents currently unrecognised species-level diversity across diverse taxa, such as *Amegillaalbigena* Lepeletier, 1841, *Andrenarussula* Lepeletier, 1841, *Lasioglossumleucozonium* (Schrank, 1781), *Nomadafemoralis* Morawitz, 1869 and *Sphecodesalternatus* Smith, 1853. Further species pairs of *Colletes*, *Hylaeus* and *Nomada* were placed into the same BINs, emphasising the need for integrative taxonomy within Iberia and across the Mediterranean Basin more broadly. These data substantially contribute to our understanding of bee genetic diversity and DNA barcodes in Iberia and provide an important baseline for ongoing taxonomic revisions in the West Palaearctic biogeographical region.

## Introduction

Bees are a diversified lineage of Hymenopteran insects, with nearly 21,000 species recognised globally (Ascher and Pickering 2023). The greatest bee diversity is found in parts of the world with a Mediterranean to xeric climate (Orr et al. 2021), most clearly south-western Australia, south-western North America, south-western South America, southern Africa and the Old World Mediterranean Basin. Within the Mediterranean Basin, the Iberian Peninsula dominates its western part and is made up of the two countries Portugal (mainland area 89,000 km^2^) and Spain (mainland area 492,000 km^2^). Peninsular Portugal hosts 725 species (Baldock et al. 2018, Wood et al. 2020, Gaspar et al. 2023, Reverté et al. 2023), of which only two bee species are currently recognised as endemic and peninsular Spain hosts approximately 1,100 species (Lhomme et al. 2020, Ortiz-Sánchez 2020).

The total Iberian fauna is represented by approximately 1,100 species belonging to the six major bee families (Apidae, Andrenidae, Colletidae, Halictidae, Megachilidae and Melittidae) with approximately 96 species endemic to the Peninsula (8.7%); these values continue to vary due to ongoing taxonomic revisions which both describe and synonymise taxa, as well as adding new records and exclusions (e.g. Ghisbain et al. (2021b), Cross (2023), Dorchin (2023), Wood (2023), Ghisbain et al. (2023a)). Iberia, therefore, hosts slightly over half of the 2,138 bee species from Europe (Ghisbain et al. 2023b) and around one third of the approximately 3,500 bee species found in the entire West Palaearctic biogeographical region (Rasmont et al. 2017 and subsequent taxonomic works).

Given the large size of the Iberian bee fauna, against the background of widely-reported bee declines (LeBuhn and Vargas Luna 2021, Zattara and Aizen 2021) and the global and regional importance of Iberian bees as pollinators of wild (e.g. Castro et al. (2013), Mendes et al. (2021)) and cultivated plants (e.g. Stefanescu et al. (2018), Gaspar et al. (2022), Mota et al. (2022)), a good understanding of this fauna is highly relevant to ensure appropriate conservation actions (LeBuhn and Vargas Luna 2021, Zattara and Aizen 2021). However, whilst the national totals for Portugal and Spain are now more or less complete, this somewhat masks how patchy the study of Iberian bees has been. Before 2018, knowledge of the Portuguese fauna was seriously incomplete (Baldock et al. 2018) and many taxonomic issues have plagued the understanding of the wider Iberian fauna. Some of the taxonomic issues remained unsolved or simply unresolved for hundreds of years (e.g. Pauly et al. (2019), Litman et al. (2021), Wood and Le Divelec (2022), Dorchin (2023), Praz and Bénon (2023), Wood (2023)) and some were only discovered and resolved much more recently (e.g. Wood et al. (2021), Wood (2022)). Many parts of Iberia remain poorly studied and much work is still required to fully understand the distribution of bees within Iberia (e.g. Álvarez Fidalgo et al. (2022), Gaspar et al. (2023)), certainly when compared to countries in Northern and Central Europe that have a long history of study and a large quantity of validated occurrence data (e.g. Peeters et al. (2012), Else and Edwards (2018), Drossart et al. (2019), Praz et al. (2023)).

In addition to the incomplete study, the Iberian bee fauna has also been seldom studied using genetic techniques as a way to taxonomically delineate its fauna (as opposed to focused population genetic studies or taxonomic studies on specific species, for example, Chávez‐Galarza et al. (2015), Cejas et al. (2019), de Sousa et al. (2023)) until very recently (e.g. Ghisbain et al. (2021a), Ghisbain et al. (2021b), Wood et al. (2021), Kasparek et al. (2023), Wood (2023)). These studies have predominantly employed DNA barcodes targeting the cytochrome oxidase I subunit (COI) of the mitochondrial genome, a technique which is widely used in bee taxonomy to delineate species (e.g.Packer and Ruz 2017, Williams et al. 2020, Brasero et al. 2021, McLaughlin et al. 2022, Praz et al. 2022, Wood 2022, Gardner and Gibbs 2023, Praz and Bénon 2023, Wood 2023). However, it is important to note that this approach can produce ambiguous results that do not consistently separate certain taxa or can lead to conflict with morphological characters (e.g. Gibbs (2018)), which can require more powerful analyses to resolve (e.g. Gueuning et al. (2020)).

More broadly, however, the DNA barcoding approach is largely supportive for the majority of existing taxonomic concepts in bees, at least in Europe (Schmidt et al. 2015). Indeed, the work of Schmidt et al. (2015) is one of the few studies to attempt to systematically barcode the entire fauna of an European country, in this case Germany (though see also Magnacca and Brown (2012), Villalta et al. (2021)). In order to meaningfully increase our knowledge of the DNA barcodes of Iberian bees and, hence, also their taxonomic status and presence in Iberia, we attempt to systematically barcode this fauna, with a focus on the species present in peninsular Portugal. We generated barcodes for species representing 46.5% (n = 512) of the bees known from the Peninsula and 20.8% (n = 20) of known endemic Iberian species. This work was conducted within the framework of the InBIO Barcoding Initiative.

## General description

### Purpose

This dataset aims to provide a contribution to an authoritative DNA barcode sequences library for Iberian bees, documenting biodiversity of this functional group, harbouring relevant pollinators in a Mediterranean hotspot. Such a library aims to enable DNA-based identification of species for both traditional molecular studies and DNA-metabarcoding studies. Furthermore, it constitutes a relevant resource for taxonomic research on Iberian bees and their distributions within and outside the Iberian Peninsula.

### Additional information

A total of 1,059 bee specimens were sequenced (Suppl. materials 1, 2, 3, 4). Fig. 2 illustrates examples of the diversity of species that are part of the dataset of distribution data and DNA barcodes of Iberian Bees. A full-length barcode of 658 bp was obtained for 1,007 specimens (95%) (Table 1, Suppl. material 2). These specimens represent 514 (46.5%) of the approximately 1,100 described bee species known to occur in the Iberian Peninsula, one species of *Melecta* being identified only to the level of genus. Furthermore, 20 taxa are Iberian endemics, representing 20.8% of the total species endemic to the Iberian Penisnsula (n = 96, subject to continuous variation due to ongoing taxonomic revisions and, hence, changing species concepts and distributions, for example, Wood (2023)). For peninsular Portugal, the corresponding values represent 70.8% of the fauna (ca. 725 species) and 50% of the endemic species (one of the only two species endemic to mainland Portugal). The dataset includes specimens from all six bee families known to occur in the Iberian Peninsula (Apidae, Andrenidae, Colletidae, Halictidae, Megachilidae, Melittidae) and 51 of the 56 (91.1%) genera present in the fauna (treating *Eucera* and *Tetralonia* as distinct genera and retaining broad *Halictus* genus including *Seladonia* and *Vestitohalictus*), lacking only *Simpanurgus* (ne species in Iberia), *Habropoda* (one species), *Camptopoeum* (one species), *Biastes* (three species) and *Rophites* (two species). These data substantially contribute to our understanding of bee barcodes with 75 species represented by barcodes for the first time in the BOLD database. Currently, 91% of the Portuguese bee species have public DNA barcodes; nevertheless, 64 species remain unrepresented in the DNA barcode reference collections (Suppl. material 5).

The BOLD BIN system uses algorithms to cluster sequences into operational taxonomic units (OTUs) that closely correspond to species (Ratnasingham and Hebert 2013). A total of 535 BINs were retrieved by BOLD (Ratnasingham and Hebert 2007) and 112 BINs are unique to our dataset (Table 1, Suppl. material 1). Sequences from two specimens were not attributed to BINs as their sequences were only 418 or 326 base pairs in length (Suppl. material 1). To our knowledge, this is the first study to systematically focus on DNA barcoding bees in the Iberian Peninsula. This study shows that DNA barcode sequences, based on the COI mitochondrial gene fragment, can be highly useful in identifying Iberian bee samples to species level with 486 of the 514 species (94.9%) assigned to unique BINs. However, 27 species were assigned to at least two BINS (Table 2); these cases are discussed below.

While is possible to consider that the taxonomy of Iberian Bees is currently better understood than in its recent past and the number of species currently known is close to the real number of species of the region, there are still some groups that require further research. High genetic diversity within a nominal species might be a signal of unresolved taxonomy, cryptic diversity or a signature of evolutionary history of the group that lead to the differentiation of isolated populations within the same species. These phenomena are well known for many organisms in the Iberian Peninsula as a result of the occurrence of several refugia within the Peninsula itself, referred to as “refugia within refugia” (Gómez and Lunt 2007, Abellán and Svenning 2014, de Sousa et al. 2023). Therefore, several species that present a rather homegeneous genetic signature across their European ranges can exhibit more than one genetically differentiated population within the Iberian Peninsula, potentially resulting in the classification of specimens in more than one BIN.

In this context, it is important to note immediately that the placement of nominal species into multiple BINs can only be considered an initial step in the recognition of potential overlooked or cryptic diversity. There are multiple cases within the global (e.g. Gibbs (2018)) or European (Gueuning et al. 2020) bee faunas where barcodes cannot resolve taxonomic issues alone and each problem must be dealt with on a case-by-case basis due to the variation in mechanisms underpinning each species or species-complex.

Overall, our results confirm that a DNA barcode library is an essential tool for incorporating DNA metabarcoding and environmental DNA techniques in biodiversity monitoring and to unveil the networks of species interactions that drive ecological and evolutionary dynamics in the ecosystems. Our results constitute a first and major step in the construction of a DNA barcode database of Iberian bee species.

**Outstanding taxonomic uncertainties**

As noted above, 27 nominal species were assigned to at least two BINs. The greatest number of cases were found in the genera *Andrena* (six species), *Lasioglossum* (five species) and *Nomada* (five species), which is not surprising since these are the three largest genera in Iberia and those that present the most difficult taxonomy.

***Amegillaalbigena* (Lepeletier, 1841)**

Three specimens of *Amegillaalbigena* are separated in two BINs, one that we consider to be the true *A.albigena* (BOLD:AEL4482) (Wood & Rasmont, *additional unpublished data*) and the other considered to be *A.talaris* (Pérez, 1895) (BOLD:AEO2968) that is not currently recognised as a species (Ghisbain & Rosa et al. 2023), but is clearly separated genetically from *Amegillaalbigena* by a maximum genetic distance of 14.02%. In the key of Ortiz-Sánchez and Jiménez-Rodríguez (1991), specimens of *A.talaris* key out as *A.fasciata* (Fabricius, 1775). True *A.fasciata* (sequenced here, BOLD:AEN9315) keys out as *A.andresi* Friese, 1914, but this species appears to be absent from Iberia (Wood & Rasmont, *unpublished data*). The formal elevation and recognition of *A.talaris* as a good species will be carried out in a future work; both *A.albigena* and *A.talaris* are widespread throughout Iberia.

**Andrenaampla Warncke, 1967**

The BOLD system identified two BINs for *Andrenaampla*, BOLD:ABA2611 from northern Portugal and BOLD:AES0278 from central Spain. The status of the species within the *Andrenaproxima*-group (including *A.ampla*, the only member of the group present in Iberia) has recently been conclusively resolved in Central Europe (McLaughlin et al. 2022). The BIN BOLD:ABA2611 containing the sequence from northern Portugal is the typical Central European BIN for *A.ampla* containing around 35 other sequences from Morocco, Spain (Granada, not sequenced in this study, published in dataset attached to Wood (2023)), France, Switzerland, Germany, Italy and the United Kingdom. The sequence from central Spain falling into BIN BOLD:AES0278 is unique and contains only this sequence. Whilst the genetic distance between the two specimens presented here (central Spain and northern Portugal) is 2.81%, the smallest distance between the sequence from central Spain and other members of the *A.ampla* BIN BOLD:AES0278 is 1.68%.

Since the overall geographic distribution of the typical *A.ampla* BIN (BOLD:AES0278) encompasses that of the unique specimen from central Spain and there do not appear to be morphological differences between specimens from central Spain and elsewhere, this observed genetic distance could simply be variation. Alternatively, the unique sequence could be a NUMT (nuclear-embedded mitochondrial DNA sequence) and, thus, does not represent a genuine mitochondrial sequence. Given these points, specimens from central Spain are considered to be conspecific with *A.ampla* pending further study.

**AndrenahesperiaSmith, 1853**

Four sequences of this widely-distributed species were generated. Two sequences from southern Portugal (Beja) fell into BOLD:AEN4997, which also includes a sequence from south-western Morocco (WPATW632-22). These sequences were strongly separated (maximum genetic distance of 10.20%) from two sequences from southern (Beja) and central (Castelo Branco) Portugal which fell into BOLD:AEO5653, with this BIN also containing three sequences from Spain (Granada, Málaga, Toledo) generated by Wood (2023). Barcoding members of the subgenusChrysandrena Hedicke, 1933, to which *Andrenahesperia* belongs, does not appear to be straightforward, with multiple mitochondrial lineages present in addition to those presented here (Wood, *unpublished data*). The three specimens from southern Portugal (Beja) were collected from the same site on the same day, with two mitochondrial lineages present and no apparent morphological differences. Further study is required, but based on morphology, the judgement is made that, at the present time, there is no justification to split up *A.hesperia*.

***Andrenalimata* Smith, 1853 and *A.thoracica* (Kirby, 1802)**

Andrena (Melandrena) species often present complex COI data (Wood 2023). Specimens identified in our study as *Andrenalimata* and *Andrenathoracica* were found to share a BIN (BOLD:AAE1815) and *A.limata* sequences were also placed in an additional BIN (BOLD:AEX3903). These two BINs were separated by a maximum genetic distance of 3.77%. Though we did not sequence any Iberian specimens of *A.nitida* (Müller, 1766), these three species combined present serious taxonomic problems regarding their consistent recognition across their range due to variable expression of morphological characters. Analysis of additional sequences from inside and outside of Iberia (Wood 2023) identified five mitochondrial clades covering these three nominal species, one for *A.thoracica*, one for *A.nitida* and three for *A.limata*.

From the data we present here, the *A.limata*/*A.thoracica* BIN BOLD:AAE1815 contains sequences from Portugal, Spain, Italy, Germany, Austria, Poland, Romania, Turkey, Lebanon, Israel and Kazakhstan and corresponds to the *limata #1* + *thoracica* lineages of Wood (2023). The *A.limata* BIN BOLD:AEX3903 contains sequences from Morocco, Portugal, Spain and France and corresponds to the *limata #2* lineage of Wood (2023). Though none of the data we presented fell into this BIN, an additional BIN BOLD:AEW6788 is available containing *A.limata*/*A.nitida* sequences from Spain, France, Switzerland, United Kingdom, Belgium, the Netherlands, Germany, Austria, Finland and Russia and corresponding to the *limata #3* + *nitida* lineages of Wood (2023). The five clades of Wood (2023), therefore, fall into three BINs, but without agreement with the existing morphological concepts.

In this context, the results presented here fit into this broader pattern of complex COI data. It is clear that more powerful genetic techniques are required to resolve species boundaries in this difficult group of species and, thus, no action is taken here.

***Andrenamorio* Brullé, 1832**

Sequences of *Andrenamorio* produced two BINs, with specimens from central Portugal (Castelo Branco) falling into both BINs, which were minimally separated by a maximum genetic distance of 2.64%. *Andrenamorio* produces a range of barcodes, with a broad species concept followed by Wood (2023) who synonymised the predominantly Iberian *A.hispania* Warncke, 1967 with it. The genetic distances reported here are considered to represent variation when considered across the entire range of *A.morio* and the genetic differentiation that it presents (Wood 2023).

***Andrenapropinqua* Schenck, 1853**

*Andrenapropinqua* has been a somewhat controversial taxon and has been placed either in synonymy with or as a subspecies of *A.dorsata* (Kirby, 1802) in the past. The current evidence supports its species status, based on analysis of Ultra-conserved Elements (Gueuning et al. 2020), as barcodes alone are insufficient to allow for consistent separation. Sequences from central Portugal (Castelo Branco and Coimbra) and southern Spain (Málaga) fell into BOLD:AAJ2115 which contains a mixture of *A.dorsata* and *A.propinqua* sequences from across Europe, whereas sequences from central and northern Portugal (Aveiro, Castelo Branco, Coimbra) fell into BOLD:AEN9473 which currently contains five sequences from Iberia only. The two BINs were separated by a maximum genetic distance of 5.41%. Based on morphological analysis, *A.dorsata* is restricted in Iberia to northern Spain, from Galicia across the Cantabrian mountains to the Pyrenees (Wood 2023). Given the complexity present, no taxonomic action is taken, as these variable barcodes are considered to represent only variation, following the study of Gueuning et al. (2020).

**AndrenarussulaLepeletier, 1841**

Praz et al. (2022) recently resolved many classification issues within the subgenusTaeniandrena Hedicke, 1933, including for *Andrenarussula*. Sequences of the true *A.russula* were generated from central Portugal (Castelo Branco, Santarém), falling into BOLD:AAZ1205, along with sequences from Portugal, Spain, Morocco and Italy. More broadly, these sequences correspond to the *A.russula* of Praz et al. (2022) and the *A.russula* #1 of Wood (2023). However, in southern Portugal (Faro, Setubal), this mitochondrial lineage is replaced by BOLD:AEN8931 which is strongly separated from the previous BIN (maximum genetic distance of 5.60%) and which conforms to the "*sp. nov. 2*" lineage of Praz et al. (2022) and the *A.russula* #2 lineage of Wood (2023). Morphologically, there are no apparent differences between the two lineages. Interestingly, BOLD:AEN8931 contains four currently unpublished sequences from France. Taxonomic revision is required to determine how to recognise this divergent mitochondrial lineage morphologically, as it likely represents yet another cryptic and undescribed *Taeniandrena* species in the west Mediterranean (Wood et al. 2021, Wood 2022, Praz et al. 2022). Given the occurrence in France, this lineage should be present also in southern and eastern Spain. No concrete action is taken here.

**AnthidiumdiademaLatreille, 1809**

Two specimens of *Anthidiumdiadema*, both from Madrid, were separated by a genetic distance of 2.81% and consequently fell into separate BINs. The two specimens were male and female, so a comparison of morphological differences is not possible. No further action is taken here without further genetic sampling.

***Anthophoraplumipes* (Pallas, 1772)**

*Anthophoraplumipes* is the most abundant anthophorine species in the West Palaearctic. Specimens from central Portugal (Coimbra) and southern Spain (Cádiz) fell into BOLD:AEN8725 which so far contains sequences from Iberia only. A specimen from northern Portugal (Braga) produced a sequence which fell into BOLD:AEO1358 which contains sequences from across the West Palaearctic. Whilst the sequences were moderately separated (maximum genetic distance of 5.86%), in a broader analysis of samples of *A.plumipes* from across the West Palaearctic (Wood, Boustani & Rasmont, *unpublished data*), no clear geographically consistent clades could be observed. Moreover, the morphology of specimens was consistent across this range, with variation predominantly comprising hair colour. A broad species concept is, therefore, retained for *A.plumipes*.

***Chelostomacampanularum* (Kirby, 1802)**

Two specimens from northern Portugal (Viseu) fell into BOLD:AAB1123 which contains typical *Chelostomacampanularum* sequences from across Europe (and Canada where the species is introduced). However, two specimens from southern Spain (Granada, Málaga) fell into BOLD:AET7155 which was separated by a maximum genetic distance of 4.25%. These divergent sequences merit further study, as the taxonomy of the small-bodied *Chelostoma* species around *C.campanularum* (subgenusFoveosmia) is challenging; no action is taken at the present time.

***Colleteshylaeiformis* Eversmann, 1852**

Specimens from central Portugal (Castelo Branco, BOLD:AEU8542) and central Spain (Ávila, BOLD:AEW0951) were assigned to different BINS (maximum genetic distance 4.70%). Both BINs contained only a single sequence and neither BIN matched BOLD:AAJ7533 which is the BIN for *Colleteshylaeiformis* sequences from Austria, France, Germany and Hungary. Further study is required to establish if Iberian material is simply genetically differentiated due to isolation by distance or whether these differences are more substantive.

**Flavipanurgusgranadensis(Warncke, 1987)**

*Flavipanurgusgranadensis* was described with a *locus typicus* in the Sierra Nevada (Granada) in southern Spain. Sequences from south-western Spain (Cádiz, BOLD:AEO4846) and south-eastern Spain (Murcia, BOLD:AEO4847) fell into distinct BINs and were separated by a maximum genetic distance of 5.93%. The BOLD:AEO4846 BIN additionally contains three sequences from Málaga and Cuenca (Wood & Boustani, *unpublished data*). Given the taxonomic complexity within *Flavipanurgus* (Cross and Wood 2018), it is not impossible that the specimen from Murcia represents an additional cryptic species, but dedicated study is necessary, as well as genotyping the population present on the Sierra Nevada.

**HalictusmaculatusSmith, 1848**

*Halictusmaculatus* is a widespread species and a sequence from northern Portugal (Porto) fell into BOLD:ACH4344 which contains sequences from Austria, France, Italy and Russia. However, a sequence from southern Spain (Granada) fell into BOLD:AEO0160 which is a unique BIN, separated by a maximum genetic distance of 3.6%. The specimen is a female and it is not impossible that it represents the Spanish endemic species *H.toparensis* Ortiz-Sánchez & Pauly, 2017 which was described from a single male from southern Spain (Almería) and which is morphologically close to *H.maculatus* (Ortiz-Sánchez and Pauly 2017). This species has recently been found to be more widespread in central Spain (Álvarez Fidalgo et al. 2023) and so is presence also in Granada is possible. Further barcoding of *H.toparensis* males is required in order to definitively associate the female sex for this species. It should also be noted that none of our material fell into BOLD:AAY5383 which is the principal European BIN for *H.maculatus* (by number of sequences and geographical distribution), containing sequences from Austria, France, Italy, Germany and Switzerland. Further study is required.

**Hoplitisravouxi(Pérez, 1902)**

*Hoplitisravouxi* is a widespread European species. A sequence from eastern Spain (Teruel, BOLD:AAI1840) fell into a BIN with sequences from France and Germany. A sequence from southern Spain (Málaga, BOLD:AET2330) was separated by a maximum genetic distance of 2.64%, with the resulting BIN being unique. Further study is required, as this southern sequence may simply represent separation by distance.

**Hylaeusangustatus(Schenck, 1861)**

Four specimens of *Hylaeusangustatus* were separated in two BINs, one that we consider to be the true *H.angustatus* (BOLD:AAK3477) containing specimens from northern and eastern Spain (León, Teruel, as well as other sequences from France, Italy, Germany and Austria) and the other containing specimens from Portugal (Castelo Branco, Viseu; BIN known from Iberia only) considered to be what was reported from Iberia as *H.angustatuspunctifrons* (Pérez, 1903) (BOLD:AEN9677) that is not currently recognised at the species level (Ghisbain & Rosa et al. 2023). However, this BIN was strongly separated genetically from *Hylaeusangustatus* by a high genetic distance of 6.84%. *Hylaeusangustatus* is a morphologically and genetically very variable species that most probably represents a species complex currently including two described species, *H.angustatus* and *H.mariannae* Theunert, 2013, this latter species being represented by an additional highly divergent BIN (Le Divelec, *unpublished data*) and which cannot be associated with Iberian material morphologically or genetically. The morphological boundaries of the two Iberian forms of *H.angustatus* are still unclear and further work is needed to clarify the taxonomy and nomenclature of this species group.

***Lasioglossumcalceatum* (Scopoli, 1763)**

For this extremely common and widespread species, sequences from northern Portugal (Aveiro, Viana do Castelo, BOLD:AAB0353) fell into the typical BIN for this species with hundreds of sequences from across the Palaearctic. However, a sequence from southern Spain (Málaga, BOLD:AEO1453) was placed in a unique BIN which was actually genetically closer to *L.albipes* (Fabricius, 1781). This BIN was separated from our typical *L.calceatum* BIN by a maximum genetic distance of 6.55%. Both *L.calceatum* and *L.albipes* are very rare in southern Iberia (Ortiz-Sánchez and Pauly 2017) and are more or less restricted to mountains. The specimen producing the unique BIN was collected at high altitude in the Sierra de las Nieves (between 1200 and 1600 m) and may represent an isolated population of *L.calceatum* or perhaps an undescribed species. Further study is required.

***Lasioglossumleucozonium* (Schrank, 1781) and *Lasioglossumleucozoniumcedri* Ebmer, 1976**

*Lasioglossumleucozonium* is a widespread Palaearctic species and a sequence from northern Portugal (Aveiro, BOLD:AAA2322) fell into the typical *L.leucozonium* BIN with hundreds of sequences from Europe to Central Asia, as well as North America where it is introduced. However, sequences from northern and central Portugal (Aveiro, Leiria) and southern Spain (Granada) fell into BOLD:AEN9620 which contains sequences from Portugal, Spain and Morocco. This BIN was separated by a maximum genetic distance of 6.42% and represents the taxon ssp. cedri which is not currently recognised at species level. A revision of the subgenusLeuchalictus Warncke, 1975 to which these taxa belong is currently in preparation (S. Flaminio, *pers. comm.*) and so no taxonomic action is taken here.

***Lasioglossummalachurum* (Kirby, 1802)**

A sequence from southern Spain (Málaga, BOLD:AAE5496) fell into the typical *L.malachurum* BIN with hundreds of sequences from the West Palaearctic. Surprisingly, a specimen from northern Portugal (Aveiro, BOLD:AEO8786) was strongly separated by a maximum genetic distance of 13.66%, though it was closest to the typical *L.malachurum* BIN. Further study is required.

**Lasioglossumtransitorium(Schenck, 1868) and *L.transitoriumplanulum* (Pérez, 1903)**

*Lasioglossumtransitoriumplanulum* is the western subspecies of *L.transitorium* and is found across Iberia (Ortiz-Sánchez and Pauly 2017). Sequences from northern Portugal (Aveiro) and eastern and southern Spain (Cuenca, Granada) fell into BOLD:AEM8844 that contains sequences from only Spain and Portugal. However, a specimen from north-eastern Spain (Lleida) fell into BOLD:AER6557 (containing additional sequences from France and Switzerland) which was separated by a maximum genetic distance of 2.01%. Detailed study is required to establish whether these represent the taxa *L.transitoriumplanulum* and *L.transitorium* *s. str.*, respectively and whether they should be treated as distinct species or not.

**Lasioglossumxanthopus(Kirby, 1802) and *L.xanthopussoreli* (Dours, 1872)**

A sequence from southern Spain (Granada, BOLD:AAE1789) fell into the typical *L.xanthopus* BIN with sequences from Belgium, France, Germany, Kyrgyzstan, the Netherlands, Switzerland, Turkey and the United Kingdom. A sequence from central Portugal (Castelo Branco) fell into BOLD:AEW1421 which contains one other sequence from Morocco (Wood, *unpublished data*) and which was separated by a maximum genetic distance of 2.01%. Further study is required to establish whether this material represents a distinct species and whether it could be referrable to the concept of *L.xanthopussoreli* (for which the type material is lost). *Lasioglossumsoreli* was described from Algeria and, although the type material is lost, if further genetic analysis of Iberian and North African *xanthopus* *sensu lato* is conducted, the taxon *soreli* can be newly delineated.

***Megachilealbisecta* (Klug, 1817)**

Sequences from southern Spain (Granada, BOLD:AEO5749) and northern Portugal (Bragança, BOLD:AEO5750) were separated by a maximum genetic distance of 2.17%. Both BINs also contained sequences from France and Italy in addition to the Spanish sequences. Given the low genetic distance, this is considered to simply represent variation.

**NomadabasalisHerrich-Schäffer, 1839**

This morphologically highly variable species was placed in three BINs, specifically specimens from central Portugal (Castelo Branco, BOLD:AEK6178, sequences also from France and Italy), eastern Spain (Cuenca, BOLD:AEN3462, Iberia only) and central Portugal (Lisbon) and central and southern Spain (Madrid, Málaga, BOLD:AEO4155, Iberia only). The maximum gentic distance between members of these BINs was 5.71%. BOLD:AEK6178 also contains sequences from France and Italy, but the two other BINs are currently known only from Iberia. *Nomadabasalis* is the brood-parasite of Eucera (Hetereucera) species, such as *E.elongatula* Vachal, 1907 (Baldock et al. 2018); this subgenus is diversified and species-rich in Iberia, offering many potential hosts for *N.basalis* and possibly unknown related taxa. A large number of names are available for *N.basalis* as, due to its morphological variation, it was described many times (Smit 2018). Taxonomic revision is required to establish whether *N.basalis* represents a species complex or whether it is simply a highly variable species.

**NomadadistinguendaMorawitz, 1874**

A sequence from northern Portugal (Aveiro, BOLD:ACY0250) clustered with *N.distinguenda* from France, Italy and the Netherlands. However, an additional sequence from northern Portugal (Bragança, BOLD:AET5764) formed a unique BIN. The maximum genetic distance between these BINs was 3.6%. Importantly, none of the Iberian material fell into BOLD:AAO6546 which contains *N.distinguenda* sequences from Bulgaria, France, Germany and Hungary or BOLD:AEA0257 which contains *N.distinguenda* sequences from Austria, Israel and Italy. Detailed taxonomic revision is clearly required to resolve species concepts within *N.distinguenda* given the genetic variation visible here.

***Nomadafemoralis* Morawitz, 1869**

A sequence from a female specimen from southern Spain (Granada, BOLD:AAI2830) clustered with *N.femoralis* sequences from Austria, France, Germany, Italy and Morocco. However, specimens from northern and southern Portugal (Aveiro, Faro) and central Spain (Ávila, BOLD:AEO6156) formed a distinct BIN, with an additional specimen from Morocco (Wood, *unpublished data*). The maximum genetic distance between the two BINs was 7.05%. This result is surprising, as *N.femoralis* is nominally one of the easiest European *Nomada* species to recognise in the male sex due to the form of shape of the anterior femorae which are produced ventrally into long triangular shapes (Smit 2018). The male specimen IBIHM124-21 from Ávila morphologically appears inseparable from typical *N.femoralis* from Central Europe, presenting these modified anterior femorae. Adding complexity, the two female specimens that fell into this BIN were originally identified as *N.femoralis* (IBIHM599-21, northern Portugal, Aveiro) and *N.corcyraea* Schmiedeknecht, 1882 (IBIHM1246-22, southern Portugal, Faro). The morphological characters allowing recognition of the females are therefore unclear and a revision of this group of species is required. The aberrant "*femoralis*" BIN (BOLD:AEO6156) is genetically closest to *N.armata* Herrich-Schäffer, 1839 and is strongly differentiated from typical *N.femoralis*, at least based on these barcoding results.

***Nomadaglaucopis* Pérez, 1890**

Most of the sequences we generated from across Spain (Granada, Guadalajara, Madrid, Málaga, BOLD:AEO3423) formed a BIN with additional sequences from France. However, a single sequence from central Portugal (Castelo Branco, BOLD:AEJ1237) was separated by a maximum genetic distance of 3.11%. Further study is required.

***Nomadaintegra* Brullé, 1832**

*Nomadaintegra* sequences were placed into three BINs, specifically with sequences from central Spain (Ávila, BOLD:ABZ1320), central Portugal (Castelo Branco) and southern Spain (Málaga, BOLD:AEN8926) and central Spain (Segovia, BOLD:AEO3904). The maximum genetic distance between these BINs was 3.43%. The sequence from Ávila (BOLD:ABZ1320) probably represents the true *N.integra*, with additional sequences from Austria, Belgium, Germany and Greece. The sequences from Portugal and southern Spain form a BIN (BOLD:AEN8926) with additional sequences from France and the sequence from Segovia (BOLD:AEO3904) forms a unique BIN. *Nomadaintegra* is a brood-parasite of members of the AndrenasubgenusChlorandrena Pérez, 1890 (Smit 2018), a subgenus that is highly diversified in Iberia (Wood 2023). Several lineages of *N.integra* may exist, possibly representing distinct species, all of which attack different *Chlorandrena* species. Detailed study is required to demonstrate this; for now, our data are presented simply as *N.integra*.

***Sphecodesalternatus* Smith, 1853 and *S.crassanus* Warncke, 1992**

Warncke (1992) described *S.crassanus* as distinct from *S.alternatus*, a position that has been largely followed by subsequent authors (Amiet et al. 2007, Bogusch and Straka 2012, Ghisbain et al. 2023b). In the female sex, *S.crassanus* can be separated from *S.alternatus* due to the denser punctation anterior to the ocellar triangle, which is slightly convex and more sparsely punctate in *S.alternatus* (Bogusch and Straka 2012). However, two specimens of *S.crassanus* showed minimal genetic differentiation from a specimen of *S.alternatus* despite this clear morphological difference and were placed in the same BIN (BOLD:ACD7802). Furthermore, an additional specimen of "*S.alternatus*" from central Portugal was strongly separated genetically from both *S.crassanus* + *S.alternatus*. This latter Portuguese specimen represents *S.algeriensis* Alfken, 1914 (considered as a subspecies of *S.alternatus* by Warncke (1992)), based on its morphology with a more densely punctate frons than typical *S.alternatus* and also because the BIN (BOLD:AEO6580) includes an unpublished sequence from Egypt; *S.algeriensis* is not currently recognised as a good species and focused taxonomic work is required to formally establish its status. The maximum genetic distance between the two BINs was 7.97%.

**Species that cannot currently be identified**

A single *Melecta* specimen from the Sierra Nevada (Granada, IBIHM072-21) fell into a unique BIN (BOLD:AEO2855) containing another unpublished sequence from Spain. The taxonomy of melectine bees is complicated and a full molecular revision is required to ensure that morphological concepts (Lieftinck 1980) actually work consistently given the extreme difficulty in species identification in this group.

**Confirmed or suspected synonymies**

***Euceralongicornis* (Linnaeus, 1758) and *Eucerahispaliensis* Pérez, 1902**

Dorchin (2023) concluded that *E.hispaliensis* was a junior synonym of the widespread *E.longicornis*. In line with this, sequences of "*E.hispaliensis*" generated from Portugal (Castelo Branco, Vila Real) and Spain (Granada, Málaga) fell into BOLD:ABZ4790, which contains *E.longicornis* sequences from across Europe. The synonymy of Dorchin (2023) is, therefore, further supported by this genetic evidence.

***Euceranigrescens* Pérez, 1879 and *Euceracodinai* Dusmet y Alonso, 1926**

It is suspected that *E.codinai* is a junior synonym of the widespread *E.nigrescens* (E. Dorchin, *pers. comm.*). The Iberian distribution appears to be that *E.nigrescens* is present in the north, being replaced in central and southern Iberia by *E.codinai* (Baldock et al. 2018). Morphological differences are minor and are predominantly based on changes in hair colour. Analysis of "*E.codinai*" sequences from southern Spain (Cádiz) and central Portugal (Castelo Branco) showed that they fell into BOLD:ABZ2397 which contains many sequences of *E.nigrescens* from across Europe eastwards to Japan. All our data are, therefore, presented as *E.nigrescens* and the formal synonymy of *E.codinai* with *E.nigrescens* is likely to be made in the future following type revision.

**Different species assigned to the same BIN**

Finally, in addition to the nominal species which were assigned to multiple BINs, there are a few species that unexpectedly fell into the same BIN despite some morphological differences. These are highlighted here.

**ColletesmarginatusSmith, 1846 and *Colletespulchellus* Pérez, 1903**

These two species are challenging to separate morphologically and old records of *C.marginatus* from Iberia can be impossible to assign to either *C.marginatus* or *C.pulchellus* without a morphological re-examination of the specimens in question (Baldock et al. 2018, M. Kuhlmann, *pers. comm.*). Three specimens nominally belonging to *C.marginatus* and *C.pulchellus* produced sequences that fell into the same BIN (BOLD:AAN3911). The specimens comprised two specimens identified as *C.pulchellus* (IBIHM1342-22; one male from Granada, IBIHM1341-22; one female from Girona; both det. M. Kuhlmann) and one specimen identified as *C.marginatus* (IBIHM429-21; one female from Segovia, det. M. Kuhlmann). This BIN contains specimens from Germany, the Netherlands, Finland, Norway, Spain and France and, thus, would certainly include sequences of the true *C.marginatus* which was described from Britain. Further study is required, as several *Colletes* species are very difficult to recognise through DNA barcoding alone, such as the *succinctus*-group (Kuhlmann et al. 2007, Zenz et al. 2021).

***Nomadasheppardana* (Kirby, 1802) and *Nomadaminuscula* Noskiewicz, 1930**

The species status of *N.minuscula* has proven controversial, more and more authors have recognised *N.minuscula* as distinct (e.g. Scheuchl 2000, Amiet et al. 2007, Schmidt et al. 2015, Scheuchl and Willner 2016, Smit 2018, Ghisbain et al. 2023b) . Schmidt et al. (2015) found that specimens belonging to these two species were assigned to two BINs - BOLD:AAP1578 for *N.minuscula* and BOLD:ABA2390 for *N.sheppardana* - and, hence, the authors supported a position of two distinct species.

Analysis of one female of "*N.sheppardana*" from central Spain (IBIMH160-21; Ávila) and one female of "*N.minuscula*" from southern Spain (IBIHM146-21; Málaga) produced sequences that fell into the same BIN (BOLD:AAP1578), i.e. the BIN of *N.minuscula* only. The two specimens clearly differ morphologically and conform to the morphological concepts of *N.sheppardana* and *N.minuscula*. The same problem occurs in other parts of Europe, with all Swiss members of this species pair producing the same barcode and falling into the *N.minuscula* BIN (Praz et al. 2023: Figure 7). It is not clear why this is occurring or why specimens of "*N.sheppardana*" from Germany represent a distinct BIN. Dedicated taxonomic work is required, including generating sequences from the United Kingdom, from which *N.sheppardana* was originally described.

***Hylaeusincongruus* Förster, 1871**

The taxonomy of the *Hylaeusgibbus*-group has a long and confused history (Dathe 1980, Straka and Bogusch 2011, Le Divelec 2022). Barcoding specimens of this group from Iberia produced sequences that fell into two BINs corresponding to *Hylaeusconfusus* Nylander, 1852 (BOLD:AAD9315) and *H.incongruus* (BOLD:ABU9205) in Central Europe. This caveat is important, as the scale of the analysis can impact interpretation, for example, when including data from the east Mediterranean (Le Divelec, *unpublished data*).

We sequenced one specimen morphologically identified as *H.confusus* from northern Spain (León) and this sequence clearly clusters with *H.confusus* sequences from across Europe (France, Switzerland, Belgium, the Netherlands, Ireland, United Kingdom, Germany, Austria, Denmark, Norway and Finland). In this context, the identification and genetic identity of *H.confusus* in Iberia does not appear to be problematic.

However, the situation with the remaining sequences generated from members of the *gibbus*-group is more challenging. A total of eight sequences were generated from across central and northern Iberia. Two sequences from north-western Iberia (INV12201; Bragança and INV12200; Ourense) were generated from specimens displaying red-markings on tergum 1 and were originally identified as *H.praenotatus* Förster, 1871. However, *H.praenotatus* as reported from Iberia by Ortiz-Sánchez et al. (2002) and Baldock et al. (2018) are *H.praenotatus* auctorum. True *H.praenotatus* is a synonym of *H.gibbus* Saunders, 1850 (Le Divelec 2022, see also Ghisbain et al. 2023b) which does not appear to occur in Iberia. The two sequences fell into the *H.incongruus* BIN BOLD:ABU9205 (additional sequences from Morocco, France, Switzerland, Italy, Belgium, the Netherlands, Germany, Austria, Czechia, Norway, Finland, Greece, Cyprus, Lebanon and Egypt). Morphologically, these *H.praenotatus* auctorum specimens do not meaningfully differ from *H.incongruus* morphologically, except in the colour of tergum 1.

Sequencing of six additional specimens with entirely dark terga from Portugal (Aveiro, Bragança, Viana do Castelo and Viseu) and Spain (Lleida) that were originally identified as *H.gibbus* produced sequences that also fell into the same *H.incongruus* BIN BOLD:ABU9205. Therefore, none of the eight specimens originally identified as either *H.praenotatus* or *H.gibbus* genetically matched with *H.gibbus* as currently delineated genetically (BIN BOLD:AAE1200, sequences from France, Switerland, Belgium, Italy, Germany, Austria and Hungary). There was a slight, but consistent genetic difference between the red-marked and dark specimens. This dark clade represents *H.purpurissatus* (Vachal, 1895), a taxon which was recently recognised at a specific level (Straka and Bogusch 2011, Le Divelec 2022). However, the morphological differences are small and inconsistent and may not represent good species (Le Divelec, *unpublished data*). Given these challenges associated with morphological delineation with species within the *gibbus*-group and these molecular results, we present all eight sequences from Iberia as *H.incongruus*; dedicated study of the *gibbus*-group across the Mediterranean Basin is required.

## Project description

### Title

The InBIO Barcoding Initiative Database: DNA Barcodes of Iberian bees

### Personnel

Thomas Wood , Romain Le Divelec and Denis Michez (taxonomists) are affiliated with the University of Mons; Hugo Gaspar (taxonomist), Sílvia Castro (ecologist) and João Loureiro (ecologist) are affiliated with the University of Coimbra; Pedro Beja (project coordinator), Sónia Ferreira (molecular biologist and IBI manager), Vanessa Mata (molecular biologist), Joana Veríssimo (molecular biologist), Teresa Luísa Silva (molecular biologist) are affiliated with the Associação BIOPOLIS / CIBIO-InBIO, University of Porto.

### Study area description

**Figure 1.**
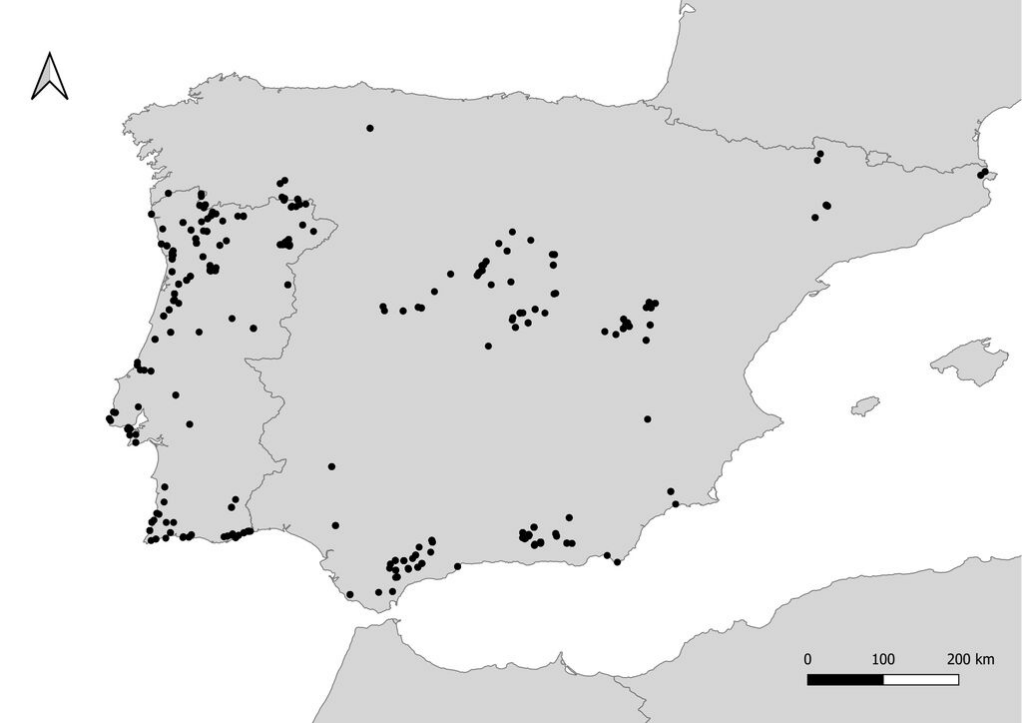
Sampling localities of the bees specimens analysed in this study.

The Iberian Peninsula (Fig. 1)

### Design description

Specimens were collected during field expeditions in the Iberian Peninsula, from 2014 to 2022 (n = 1,059 Fig. 1, Suppl. material 1), with nearly 90% of specimens collected in the period between 2019 and 2022. The majority of the specimens are deposited in the research collection of Thomas Wood (soon to be moved to the Naturalis Biodiversity Center, the Netherlands) (60%) and in the FLOWer Lab collection at the University of Coimbra (Portugal) (34%). Additional specimens are deposited in the Andreia Penado collection at the Natural History and Science Museum of the University of Porto (MHNC-UP) (Portugal) and in the InBIO Barcoding Initiative (IBI) reference collection (Vairão, Portugal).

DNA was extracted using two different kits: 96-Well Plate Animal Genomic DNA Mini-Preps Kit (Bio Basic, Ontario, Canada) or QIAmp DNA Micro Kit (Qiagen, Hilden, Germany). QIAmpDNA Micro Kit is designed to extract higher concentrations of genetic material from samples with small amounts of DNA. DNA amplification was performed using two different primer pairs, that amplify two overlapping fragments of the same 658 bp region of the COI mitochondrial gene. We used two primer pairs, LCO1490) Folmer et al. 1994) +Ill_C_R (Shokralla et al. 2015) and BF3 (Elbrecht et al. 2019) + BR2 (Elbrecht and Leese 2017) (henceforth referred to as LC and B3, respectively) to amplify two overlapping fragments of 325 bp and 418 bp, respectively. PCRs were performed in 10 μl reactions, containing 5 μl of Multiplex PCRMaster Mix (Qiagen, Germany), 0.3 – 0.4 mM of each primer and 1-2 μl ofDNA, with the remaining volume in water. PCR reactions were performed in T100 Thermal Cycler (Bio-Rad, California, USA) and carried out with an initial denaturation at 95ºC for 15 min, followed by 45 cycles at 95ºC for 30 sec, 45ºC for 45 sec, 72ºC for 45 sec; and a final elongation step at 60ºC for 10 min. All PCR products were analysed by agarose gel electrophoresis and samples selected for sequencing proceeded for a indexing PCR. One of two types of index was used for each sequencing run. For Nextera indexes (Illumina, USA), PCR products were previously pooled into one plate as described in Shokralla et al. (2015) and then the indexing PCR was performed according to the manufacturer's instructions. When using custom indexes, based on Meyer and Kircher (2010), no pooling was required. A second PCR was then performed where the indexes and Illumina sequencing adapters were attached to the PCR product. The index PCR was performed in a volume of 10 μl, including 5 μl of Phusion® High-Fidelity PCR Kit (NewEngland Biolabs, U.S.A.) or KAPA HiFi PCR Kit (KAPA Biosystems, U.S.A.), 0.5 μl of each index and 2 μl of diluted PCR product (usually 1:4). This PCR reaction runs for 10 cycle at an annealing temperature of 55ºC. The amplicons were purified using AMPure XP beads (Beckman Coulter Genomics, Massachusetts, United States) before quantification using NanoDrop 1000 (Thermo Fisher Scientific, Massachusetts, USA). Concentrations between samples were then normalised and samples were pooled, based on used primer sets. Quantification of final pools was assessed through qPCR using the KAPA Library Quantification Kit Illumina® Platforms (Kapa Biosystems) and the 2200 Tapestation System (Agilent Technologies, California, USA) was used for fragment length analysis as described by Paupério et al. (2018). Sequencing was conducted at CIBIO facilities in an Illumina MiSeq benchtop system, using V2 MiSeq sequencing kits (2x 250 bp). Illumina sequencing reads were processed using OBITools (Boyer et al. 2015) and VSEARCH (Rognes et al. 2016). Briefly, paired-end reads were aligned, collapsed into exact sequence variants, filtered by length, denoised and checked for chimeras. The resulting sequences from both LC and BH fragments of each sample were further assembled using CAP3 (Huang and Madan 1999) to produce a single 658 bp contig per sample. The obtained DNA sequences were then compared against the Barcode of Life Data Systems (BOLD) database (Ratnasingham and Hebert 2007) using the built-in identification engine, based on the BLAST algorithm. Sequences were submitted to the BOLD database and the Barcode Index Numbers (BIN) for every sequence were retrieved and analysed (Suppl. materials 2, 1Suppl. materials 1, 2, 3).

### Funding

The present work was funded by National Funds through FCT-Fundação para a Ciência e a Tecnologia in the scope of the project LA/P/0048/2020. InBIO Barcoding Initiative is co-funded by the European Union’s Horizon 2020 Research and Innovation Programme under grant agreement No 668981 and the project PORBIOTA—Portuguese E-Infrastructure for Information and Research on Biodiversity (POCI-01-0145- FEDER-022127), supported by Operational Thematic Program for Competitiveness and Internationalization (POCI), under the PORTUGAL 2020 Partnership Agreement, through the European Regional Development Fund (FEDER) and by Horizon Europe under the Biodiversity, Circular Economy and Environment call (REA.B.3); co-funded by the Swiss State Secretariat for Education, Research and Innovation (SERI) under contract number 22.00173; and by the UK Research and Innovation under the Department for Business, Energy and Industrial Strategy’s Horizon Europe Guarantee Scheme. JL, HG and SC were funded by the Integrated Program of Scientific Research and Technological Development CULTIVAR (CENTRO- 01- 0145-FEDER-000020), co-financed by the Regional Operational Programme Centro 2020, Portugal 2020 and European Union, through the European Fund for Regional Development (ERDF) and by The Portuguese Foundation for Science and Technology (FCT – Fundação para a Ciência e a Tecnologia, I.P.) within the project UID/BIA/04004/2020. SF, VM and SC were funded by the FCT through the programme ‘Stimulus of Scientific Employment, Individual Support—3rd Edition (https://doi.org/10.54499/2020.03526.CEECIND/CP1601/CT0010; https://doi.org/10.54499/2020.02547.CEECIND/CP1601/CT0006; 2021.02697.CEECIND). JV and HG were funded by PhD grants (SFRH/BD/133159/2017; 2023.01736.BD) from FCT. PR is supported by the EU Project ORBIT (DG Env 09.029901/2021/848268/SER/ENV.D.2). TJW is supported by an F.R.S.-FNRS Fellowship “Chargé de recherches”. DM, RLD and TJM are supported by SPRING - Strengthening Pollinator Recovery through Indicators and Monitoring, EC DG ENV project Contract No: 09.02001/2021/847887/SER/ENV.D.2; ORBIT- Taxonomic resources for European bees, EC DG Env project Contract No 09.029901/2021/848268/SER/ENV.D.2; PULSE - Providing technical and scientific support in measuring the pulse of European biodiversity using the Red List Index, EC DG ENV project Contract No 07.027755/2020/840209/SER/ENV.D.2; SAFEGUARD - Safeguarding European wild pollinators, H2020 grant agreement No. 101003476.

## Sampling methods

### Sampling description

Specimens were captured during direct searches of the environment, using mainly aerial-nets and hand-held sweep-nets. Specimens were euthanised, often through exposure to ethyl acetate, either subsequently pinned and dried immediately (the same day) or kept at -4ºC for some days until pinning and drying. From each specimen, one tissue sample (a leg, sometimes two legs depending on specimen size) was removed and stored in 96% ethanol for DNA extraction at the IBI collection. Photos of vouchers of all new species /BINs to BOLD will be made publicly available later in the year.

### Quality control

All DNA barcode sequences were compared against the BOLD database and the 99 top results were inspected in order to detect possible problems due to contaminations or misidentifications. Prior to GBIF submission, data were checked for errors and inconsistencies with OpenRefine 3.3 (http://openrefine.org).

### Step description

Specimens were collected in 140 different localities in Portugal and 120 localities in Spain. Collections were carried out between 2014 and 2022. Specimens were collected during fieldwork by direct search of specimens, by sweeping the vegetation with a hand-net. Captured specimens were deposited in reference collections at the Naturalis Biodiversity Center (the Netherlands) (60%) and in the FLOWer Lab collection at the University of Coimbra (Portugal) (34%). Additional specimens are deposited in the Andreia Penado collection at the Natural History and Science Museum of the University of Porto (MHNC-UP) (Portugal) and in the InBIO Barcoding Initiative (IBI) reference collection (Vairão, Portugal). Specimens were morphologically identified using stereoscopic microscopes (Wild Heerbrugg M5). DNA barcodes were sequenced from all specimens. For this, one leg (sometimes two legs depending on specimen size) was removed from each individual, DNA was then extracted and two overlying DNA fragments were amplified and sequenced to 658 bp COI DNA barcode fragment. All obtained sequences were submitted to BOLD and GenBank databases and, to each sequenced specimen, the morphological identification, was contrasted with the results of the BLAST of the newly-generated DNA barcodes in the BOLD Identification Engine. Prior to submission to GBIF, data were checked for errors and inconsistencies with OpenRefine 3.3 (http://openrefine.org/).

## Geographic coverage

### Description

Specimens were collected in the Iberian Peninsula, 590 specimens from 140 localities in mainland Portugal and 469 specimens from 120 localities in mainland Spain (Fig. 1, Suppl. material 1).

### Coordinates

0.691 and -9.484 Latitude; 42.983 and 36.268 Longitude.

## Taxonomic coverage

### Description

This dataset is composed of data relating to 1,059 bee specimens. All specimens were determined to species level. Overall, 514 described species are represented in the dataset. These species belong to six families: Andrenidae, Apidae, Colletidae, Halictidae, Megachilidae and Melittidae.

## Collection data

### Collection name

InBIO Barcoding Initiative

### Collection identifier

4ec2b246-f5fa-4b90-9a8d-ddafc2a3f970

### Specimen preservation method

Dry

### Curatorial unit

DNA extractions - 1 to 1,059.

## Usage licence

### Usage licence

Creative Commons Public Domain Waiver (CC-Zero)

## Data resources

### Data package title

The InBIO Barcoding Initiative Database: DNA barcodes of Iberian bees

### Resource link

http://dx.doi.org/10.5883/DS-IBIHY01

### Number of data sets

1

### Data set 1.

#### Data set name

DS-IBIHY01 IBI Bees of Iberia

#### Data format

dwc, xml, tsv, fasta

#### Download URL

http://www.boldsystems.org/index.php/Public_SearchTerms?query=DS-IBIHY01

#### Description

The InBIO Barcoding Initiative Database: DNA Barcodes of Iberian bees dataset can be downloaded from the Public Data Portal of BOLD (http://www.boldsystems.org/index.php/Public_SearchTerms?query=DS-IBIHY01) in different formats (data as dwc, xml or tsv and sequences as fasta files). Alternatively, BOLD users can log-in and access the dataset via the Workbench platform of BOLD. All records are also searchable within BOLD, using the research function of the database.

**Data set 1.** 

Column label	Column description
processid	Unique identifier for the sample.
sampleid	Identifier for the sample being sequenced, i.e. IBI catalogue number at Cibio-InBIO, Porto University. Often identical to the "Field ID" or "Museum ID".
recordID	Identifier for specimen assigned in the field.
catalognum	Catalogue number.
fieldnum	Field number.
institution_storing	The full name of the institution that has physical possession of the voucher specimen.
bin_uri	Barcode Index Number system identifier.
phylum_taxID	Phylum taxonomic numeric code.
phylum_name	Phylum name.
class_taxID	Class taxonomic numeric code.
class_name	class_name.
order_taxID	Order taxonomic numeric code.
order_name	Order name.
family_taxID	Family taxonomic numeric code.
family_name	Family name.
subfamily_taxID	Subfamily taxonomic numeric code.
subfamily_name	Subfamily name.
genus_taxID	Genus taxonomic numeric code.
genus_name	Genus name.
species_taxID	Species taxonomic numeric code.
species_name	Species name.
identification_provided_by	Full name of primary individual who assigned the specimen to a taxonomic group.
identification_method	The method used to identify the specimen.
voucher_status	Status of the specimen in an accessioning process (BOLD controlled vocabulary).
tissue_type	A brief description of the type of tissue or material analysed.
collectors	The full or abbreviated names of the individuals or team responsible for collecting the sample in the field.
lifestage	The age class or life stage of the specimen at the time of sampling.
sex	The sex of the specimen.
lat	The geographical latitude (in decimal degrees) of the geographic centre of a location.
lon	The geographical longitude (in decimal degrees) of the geographic centre of a location.
elev	Elevation of sampling site (in metres above sea level).
country	The full, unabbreviated name of the country where the organism was collected.
province_state	The full, unabbreviated name of the province ("Distrito" in Portugal) where the organism was collected.
region	The full, unabbreviated name of the municipality ("Concelho" in Portugal) where the organism was collected.
exactsite	Additional name/text description regarding the exact location of the collection site relative to a geographic relevant landmark.
habitat	A category or description of the habitat in which the event occurred.
sampling_protocol	The name of, reference to, or description of the method or protocol used during an event.
subspecies_taxID	subspecies taxonomic numeric code.
subspecies_name	subspecies name.

## Supplementary Material

C1370D3F-2EC4-57C1-BC17-EB14DCEC124E 10.3897/BDJ.12.e117172.suppl1 Supplementary material 1 DS-IBIHY01 IBI Bees of Iberia library - Specimen details Data type Record information - specimen data Brief description The file includes information about all records in BOLD for the DS-IBIHY01 IBI Bees of Iberia library. It contains collection and identification data. File: oo_949902.txt https://binary.pensoft.net/file/949902 Thomas Wood, Hugo Gaspar, Romain Le Divelec, Andreia Penado, Teresa Luisa Silva, Vanessa Mata, Joana Veríssimo, Deniz Michez, Silvia Castro, João Loureiro, Pedro Beja, Sónia Ferreira

5A23BFD8-3222-5729-BA7C-C979EA0F3162 10.3897/BDJ.12.e117172.suppl2 Supplementary material 2 DS-IBIHY01 IBI Bees of Iberia library - DNA sequences Data type Genomic data, DNA sequences Brief description COI sequences in fasta format. Each sequence is identified by the BOLD ProcessID, species name, marker and GenBank accession number, separated by pipe. File: oo_949900.fas https://binary.pensoft.net/file/949900 Thomas Wood, Hugo Gaspar, Romain Le Divelec, Andreia Penado, Teresa Luisa Silva, Vanessa Mata, Joana Veríssimo, Deniz Michez, Silvia Castro, João Loureiro, Pedro Beja, Sónia Ferreira

2F7134FE-98C5-5C38-AF5A-29E8CAB3D804 10.3897/BDJ.12.e117172.suppl3 Supplementary material 3 DS-IBIHY01 IBI Bees of Iberia library - Darwin Core Standard Data type Record information - specimen data in Darwin Core Standard format Brief description The file includes information about all records in BOLD for the DS-IBIHY01 IBI Bees of Iberia library. It contains collection and identification data. File: oo_950147.txt https://binary.pensoft.net/file/950147 Thomas Wood, Hugo Gaspar, Romain Le Divelec, Andreia Penado, Teresa Luisa Silva, Vanessa Mata, Joana Veríssimo, Deniz Michez, Silvia Castro, João Loureiro, Pedro Beja, Sónia Ferreira

5C78E33D-AB40-5C99-89FB-3321ECE0A531 10.3897/BDJ.12.e117172.suppl4 Supplementary material 4 NJ tree of bee DNA barcodes Data type Phylogenetic tree Brief description NJ tree of all Bee DNA barcodes generated in the InBIO Barcoding Initiative Database: DNA barcodes of Iberian Bees. File: oo_980838.pdf https://binary.pensoft.net/file/980838 Thomas Wood, Hugo Gaspar, Romain Le Divelec, Andreia Penado, Teresa Luisa Silva, Vanessa Mata, Joana Veríssimo, Deniz Michez, Silvia Castro, João Loureiro, Pedro Beja, Sónia Ferreira

0D47F5E8-3731-55A1-A771-5CAABAAC33B1 10.3897/BDJ.12.e117172.suppl5 Supplementary material 5 BOLD Systems Hitlist of Portuguese Bees Data type Species list Brief description List of bee species with occurrence known in Portugal, but lacking specimens and/or sequences and/or DNA barcodes in BOLD database on 8 February 2024. File: oo_984354.xlsx https://binary.pensoft.net/file/984354 Thomas Wood, Sónia Ferreira

## Figures and Tables

**Figure 2a.**
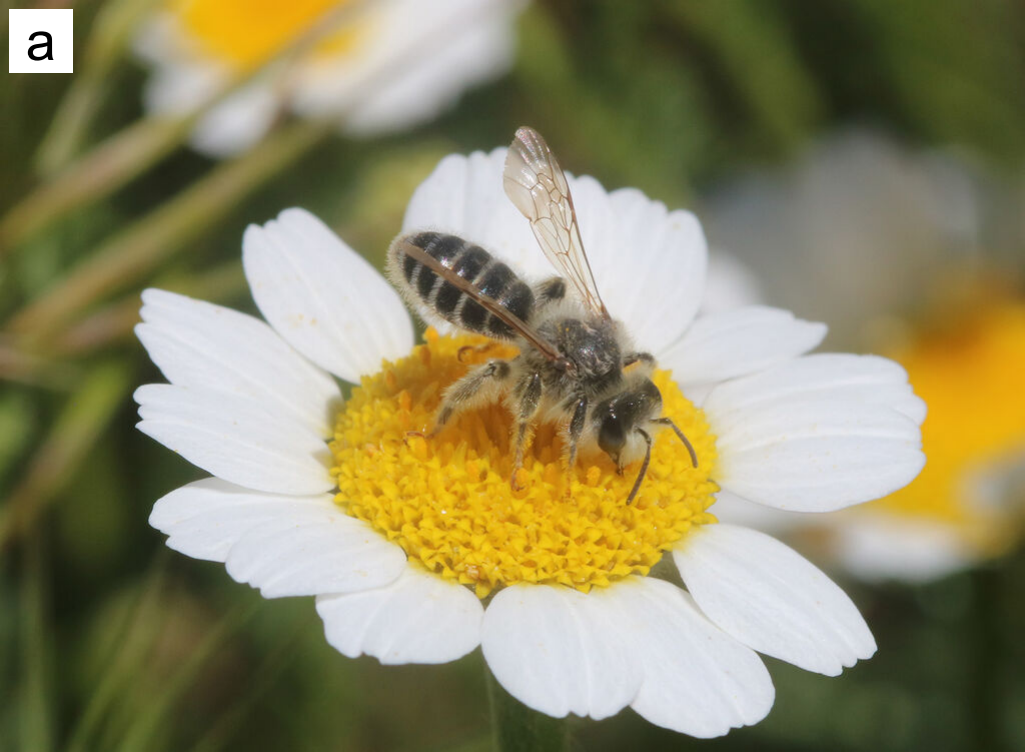
*Andrenaleucolippa* Pérez, 1895 (Andrenidae) - BIN URI BOLD:AEO2473.

**Figure 2b.**
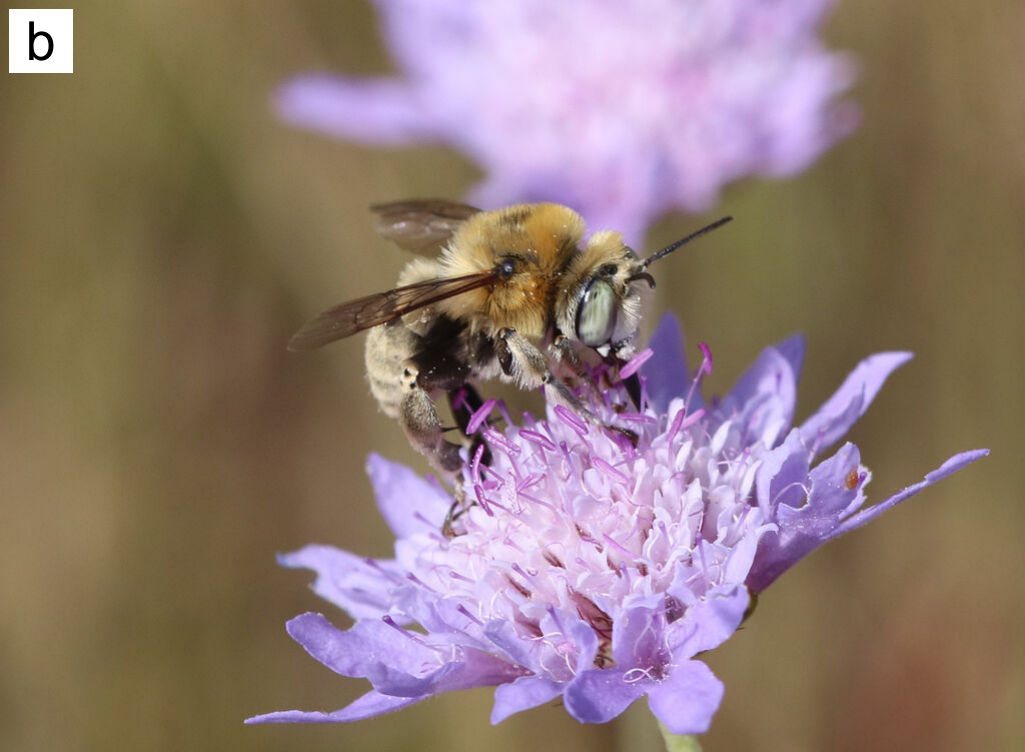
*Anthophorapodagra* Lepeletier, 1841 (Apidae) - BIN URI BOLD:AEO2867.

**Figure 2c.**
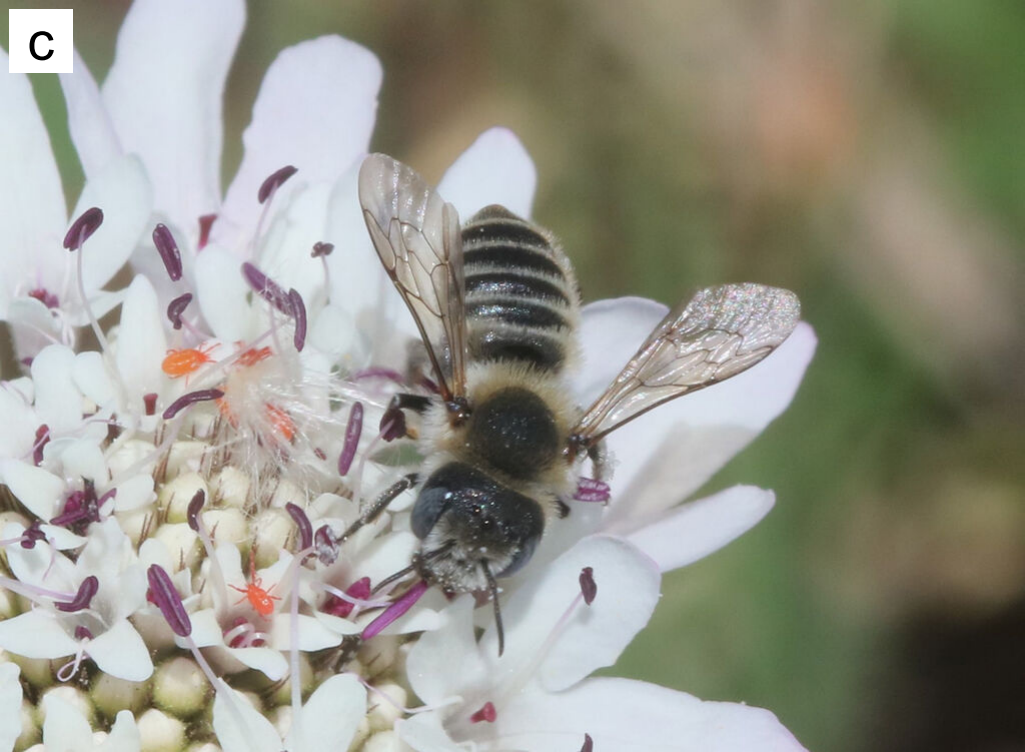
*Hoplitisbisulca* (Gerstäcker, 1869) (Megachilidae) - BIN URI BOLD:AEO0529.

**Figure 2d.**
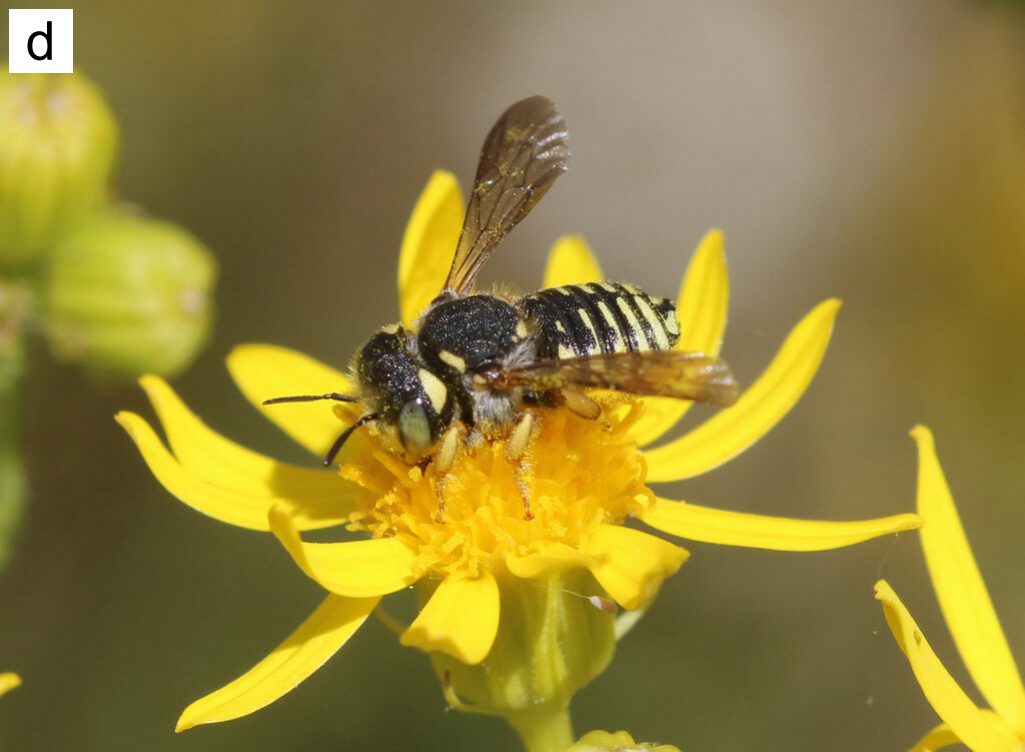
*Pseudoanthidiumeximium* (Giraud, 1863) (Megachilidae) - BIN URI BOLD:AEO0467.

**Figure 2e.**
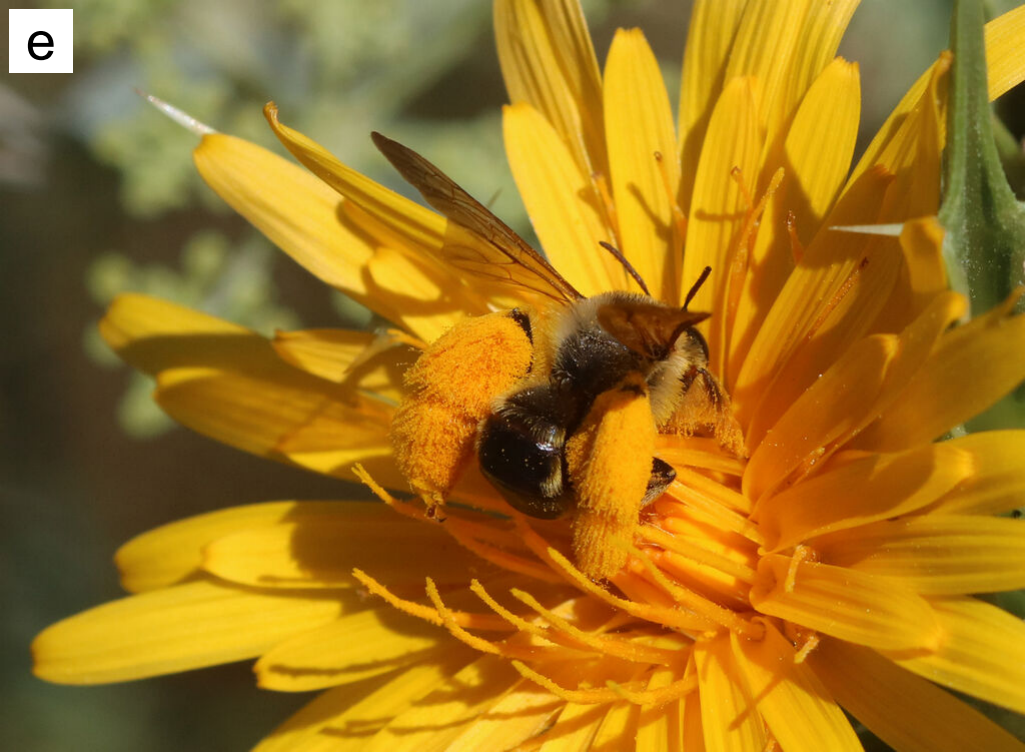
*Dasypodavisnaga* (Rossi, 1790) (Melittidae) - BIN URI BOLD:AEO0075.

**Figure 2f.**
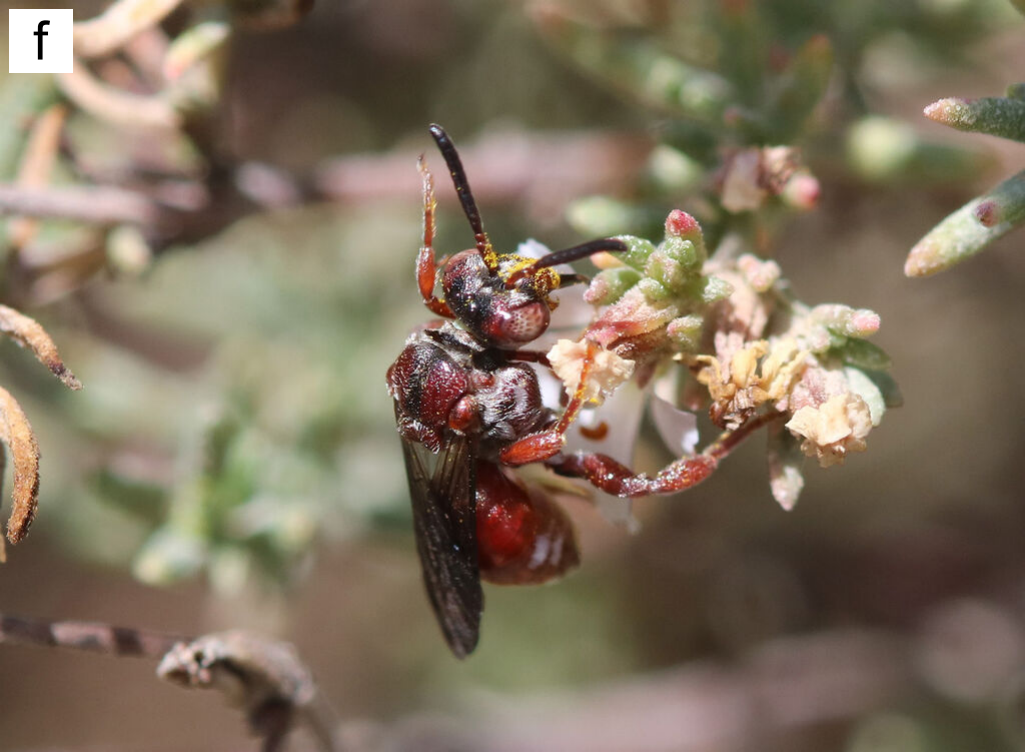
*Pasitesmaculatus* Jurine, 1807 (Apidae) - BIN URI BOLD:AAL3976.

**Table 1.** List of species that were collected and DNA barcoded for this project. "IBI code" is the Sample ID, while "BOLD code" is the Process ID attributed by BOLD. In the Species column, # indicates taxa for which no DNA barcode was available prior to this study; in the BOLD BIN column, ' indicates unique BINs.

Family	Species	IBI code	BOLD code	BOLD BIN	GenBank
Andrenidae	*Andrenaaerinifrons* Dours, 1873	INV12500	IBIHM471-21	BOLD:ADJ3594	OR795910
Andrenidae	*Andrenaaerinifrons* Dours, 1873	INV12633	IBIHM1034-22	BOLD:AET5046'	OR796236
Andrenidae	*Andrenaaerinifrons* Dours, 1873	INV12827	IBIHM1228-22	BOLD:AET5046'	OR795930
Andrenidae	*Andrenaafzeliella* (Kirby, 1802)	INV12647	IBIHM1048-22	BOLD:AAP2754	OR795919
Andrenidae	*Andrenaagilissima* (Scopoli, 1770)	INV12501	IBIHM472-21	BOLD:AEO1903	OR795937
Andrenidae	*Andrenaagilissima* (Scopoli, 1770)	INV12502	IBIHM473-21	BOLD:AEO1903	OR796727
Andrenidae	*Andrenaagilissima* (Scopoli, 1770)	INV13382	IBIHM952-21	BOLD:AEO1903	OR796085
Andrenidae	*Andrenaalfkenella* Perkins, 1914	INV12503	IBIHM474-21	BOLD:AAK0552	OR796780
Andrenidae	*Andrenaalfkenella* Perkins, 1914	INV13052	IBIHM623-21	BOLD:AAK0552	OR796891
Andrenidae	*Andrenaalfkenella* Perkins, 1914	INV13198	IBIHM769-21	BOLD:AAK0552	OR796142
Andrenidae	*Andrenaalfkenella* Perkins, 1914	INV13199	IBIHM770-21	BOLD:AAK0552	OR796444
Andrenidae	*Andrenaalluaudi* Benoist, 1961#	INV12504	IBIHM475-21	BOLD:AEN9732'	OR796776
Andrenidae	*Andrenaalluaudi* Benoist, 1961#	INV12505	IBIHM476-21	BOLD:AEN9732'	OR796724
Andrenidae	*Andrenaalma* Warncke, 1975	INV12828	IBIHM1229-22	BOLD:AEN9431'	OR796777
Andrenidae	*Andrenaalma* Warncke, 1975	INV12829	IBIHM1230-22	BOLD:AEN9431'	OR796133
Andrenidae	*Andrenaalma* Warncke, 1975	INV13377	IBIHM947-21	BOLD:AEN9431'	OR796746
Andrenidae	*Andrenaampla* Warncke, 1967	INV13168	IBIHM739-21	BOLD:ABA2611	OR796274
Andrenidae	*Andrenaampla* Warncke, 1967	INV12635	IBIHM1036-22	BOLD:AES0278'	OR796123
Andrenidae	*Andrenaangustior* (Kirby, 1802)	INV12638	IBIHM1039-22	BOLD:AAE5176	OR796639
Andrenidae	*Andrenaangustior* (Kirby, 1802)	INV13174	IBIHM745-21	BOLD:AAE5176	OR796475
Andrenidae	*Andrenaangustior* (Kirby, 1802)	INV13175	IBIHM746-21	BOLD:AAE5176	OR795960
Andrenidae	*Andrenaangustior* (Kirby, 1802)	INV13176	IBIHM747-21	BOLD:AAE5176	OR796180
Andrenidae	*Andrenaangustior* (Kirby, 1802)	INV13180	IBIHM751-21	BOLD:AAE5176	OR796837
Andrenidae	*Andrenaangustior* (Kirby, 1802)	INV13181	IBIHM752-21	BOLD:AAE5176	OR796285
Andrenidae	*Andrenaangustior* (Kirby, 1802)	INV13182	IBIHM753-21	BOLD:AAE5176	OR796538
Andrenidae	*Andrenaangustior* (Kirby, 1802)	INV13183	IBIHM754-21	BOLD:AAE5176	OR796374
Andrenidae	*Andrenaangustior* (Kirby, 1802)	INV13184	IBIHM755-21	BOLD:AAE5176	OR796685
Andrenidae	*Andrenaangustior* (Kirby, 1802)	INV13188	IBIHM759-21	BOLD:AAE5176	OR796929
Andrenidae	*Andrenaangustior* (Kirby, 1802)	INV13192	IBIHM763-21	BOLD:AAE5176	OR796292
Andrenidae	*Andrenaangustior* (Kirby, 1802)	INV13196	IBIHM767-21	BOLD:AAE5176	OR796082
Andrenidae	*Andrenaangustior* (Kirby, 1802)	INV13226	IBIHM797-21	BOLD:AAE5176	OR796580
Andrenidae	*Andrenaangustior* (Kirby, 1802)	INV13227	IBIHM798-21	BOLD:AAE5176	OR796304
Andrenidae	*Andrenaantigana* Pérez, 1895	INV12506	IBIHM477-21	BOLD:AEO4687	OR796250
Andrenidae	*Andrenaantigana* Pérez, 1895	INV12507	IBIHM478-21	BOLD:AEO4687	OR796852
Andrenidae	*Andrenaassimilis* Radoszkowski, 1876	INV12627	IBIHM1028-22	BOLD:AEU3679'	OR796869
Andrenidae	*Andrenabaetica* Wood, 2020	INV12821	IBIHM1222-22	BOLD:AEN8421	OR796472
Andrenidae	*Andrenabellidis* Pérez, 1895#	INV12824	IBIHM1225-22	BOLD:AET0142'	OR796068
Andrenidae	*Andrenabellidis* Pérez, 1895#	INV12825	IBIHM1226-22	BOLD:AET0142'	OR796765
Andrenidae	*Andrenabenoisti* Wood, 2021	INV12637	IBIHM1038-22	BOLD:AEO0062	OR796387
Andrenidae	*Andrenabicolor* Fabricius, 1775	INV13051	IBIHM622-21	BOLD:AAD0135	OR796905
Andrenidae	*Andrenabicolorata* (Rossi, 1790)	INV12628	IBIHM1029-22	BOLD:AEK9626	OR796512
Andrenidae	*Andrenabicolorata* (Rossi, 1790)	INV12826	IBIHM1227-22	BOLD:AEK9626	OR796382
Andrenidae	*Andrenabimaculata* (Kirby, 1802)	INV12508	IBIHM479-21	BOLD:AAY9952	OR796913
Andrenidae	*Andrenabimaculata* (Kirby, 1802)	INV13398	IBIHM968-21	BOLD:AAY9952	OR796445
Andrenidae	*Andrenacinerea* Brullé, 1832	INV12509	IBIHM480-21	BOLD:AEM8298	OR796689
Andrenidae	*Andrenacinerea* Brullé, 1832	INV12823	IBIHM1224-22	BOLD:AEM8298	OR796590
Andrenidae	*Andrenacombinata* (Christ, 1791)	INV12634	IBIHM1035-22	BOLD:AAK0530	OR796412
Andrenidae	*Andrenacombinata* (Christ, 1791)	INV12649	IBIHM1050-22	BOLD:AAK0530	OR796847
Andrenidae	*Andrenadiscors* Erichson, 1841	INV12546	IBIHM517-21	BOLD:AEI2585	OR796311
Andrenidae	*Andrenadjelfensis* Pérez, 1895	INV12818	IBIHM1219-22	BOLD:AET0920	OR796145
Andrenidae	*Andrenadoursanacitreola* Warncke, 1975	INV12822	IBIHM1223-22	BOLD:AET0918	OR796522
Andrenidae	*Andrenafabrella* Pérez, 1903	INV12639	IBIHM1040-22	BOLD:AEO4550	OR796649
Andrenidae	*Andrenafabrella* Pérez, 1903	INV12645	IBIHM1046-22	BOLD:AEO4550	OR796248
Andrenidae	*Andrenaferrugineicrus* Dours, 1872	INV12512	IBIHM483-21	BOLD:AEN6074	OR795954
Andrenidae	*Andrenaflavipes* Panzer, 1799	INV12513	IBIHM484-21	BOLD:ACX2946	OR796075
Andrenidae	*Andrenaflavipes* Panzer, 1799	INV12817	IBIHM1218-22	BOLD:ACX2946	OR796258
Andrenidae	*Andrenaflavipes* Panzer, 1799	INV13161	IBIHM732-21	BOLD:ACX2946	OR796504
Andrenidae	*Andrenaflavipes* Panzer, 1799	INV13162	IBIHM733-21	BOLD:ACX2946	OR796762
Andrenidae	*Andrenaflavipes* Panzer, 1799	INV13163	IBIHM734-21	BOLD:ACX2946	OR796170
Andrenidae	*Andrenaflavipes* Panzer, 1799	INV13164	IBIHM735-21	BOLD:ACX2946	OR796714
Andrenidae	*Andrenaflavipes* Panzer, 1799	INV13165	IBIHM736-21	BOLD:ACX2946	OR796733
Andrenidae	*Andrenaflavipes* Panzer, 1799	INV13392	IBIHM962-21	BOLD:ACX2946	OR796291
Andrenidae	*Andrenaflorea* Fabricius, 1793	INV12514	IBIHM485-21	BOLD:AAK0287	OR796059
Andrenidae	*Andrenaflorea* Fabricius, 1793	INV13166	IBIHM737-21	BOLD:AAK0287	OR796159
Andrenidae	*Andrenaflorea* Fabricius, 1793	INV13169	IBIHM740-21	BOLD:AAK0287	OR796279
Andrenidae	*Andrenaflorea* Fabricius, 1793	INV13172	IBIHM743-21	BOLD:AAK0287	OR796755
Andrenidae	*Andrenaflorentina* Magretti, 1883	INV12515	IBIHM486-21	BOLD:ADZ9682	OR795967
Andrenidae	*Andrenaflorentina* Magretti, 1883	INV13193	IBIHM764-21	BOLD:ADZ9682	OR796604
Andrenidae	*Andrenaflorentina* Magretti, 1883	INV13387	IBIHM957-21	BOLD:ADZ9682	OR796889
Andrenidae	*Andrenaflorentina* Magretti, 1883	INV13388	IBIHM958-21	BOLD:ADZ9682	OR796719
Andrenidae	*Andrenaflorentina* Magretti, 1883	INV13394	IBIHM964-21	BOLD:ADZ9682	OR796136
Andrenidae	*Andrenafulica* Warncke, 1974	INV12629	IBIHM1030-22	BOLD:AEN6978	OR796196
Andrenidae	*Andrenafulica* Warncke, 1974	INV13379	IBIHM949-21	BOLD:AEN6978	OR796041
Andrenidae	*Andrenafulvago* (Christ, 1791)	INV12516	IBIHM487-21	BOLD:AAK0296	OR795999
Andrenidae	*Andrenafuscipes* (Kirby, 1802)	INV15655	IBIHY003-22	BOLD:AAE1706	OR796101
Andrenidae	*Andrenafuscipes* (Kirby, 1802)	INV15677	IBIHY020-22	BOLD:AAE1706	OR796027
Andrenidae	*Andrenafuscosa* Erichson, 1835	INV12517	IBIHM488-21	BOLD:AEO6270	OR796949
Andrenidae	*Andrenagranulosa* Pérez, 1902	INV12518	IBIHM489-21	BOLD:AAR3409	OR796195
Andrenidae	*Andrenahaemorrhoa* (Fabricius, 1781)	INV12519	IBIHM490-21	BOLD:AAD8891	OR796468
Andrenidae	*Andrenahedikae* Jaeger, 1934	INV12520	IBIHM491-21	BOLD:AEO3736	OR796737
Andrenidae	*Andrenahedikae* Jaeger, 1934	INV12521	IBIHM492-21	BOLD:AEO3736	OR796601
Andrenidae	*Andrenahedikae* Jaeger, 1934	INV13418	IBIHM988-21	BOLD:AEO3736	OR796558
Andrenidae	*Andrenahesperia* Smith, 1853	INV12836	IBIHM1237-22	BOLD:AEN4997	OR796647
Andrenidae	*Andrenahesperia* Smith, 1853	INV12841	IBIHM1242-22	BOLD:AEN4997	OR796360
Andrenidae	*Andrenahesperia* Smith, 1853	INV12842	IBIHM1243-22	BOLD:AEO5653	OR796396
Andrenidae	*Andrenahesperia* Smith, 1853	INV13376	IBIHM946-21	BOLD:AEO5653	OR796299
Andrenidae	*Andrenahumilis* Imhoff, 1832	INV12524	IBIHM495-21	BOLD:AER4065	OR796904
Andrenidae	*Andrenahypopolia* Schmiedeknecht, 1884	INV12630	IBIHM1031-22	BOLD:AAJ2215	OR796330
Andrenidae	*Andrenahypopolia* Schmiedeknecht, 1884	INV12648	IBIHM1049-22	BOLD:AAJ2215	OR796871
Andrenidae	*Andrenahystrix* Schmiedeknecht, 1883	INV12631	IBIHM1032-22	BOLD:AEO0162	OR796029
Andrenidae	*Andrenahystrix* Schmiedeknecht, 1883	INV12632	IBIHM1033-22	BOLD:AEO0162	OR796383
Andrenidae	*Andrenaimpressa* Warncke, 1967	INV12646	IBIHM1047-22	BOLD:AEO5002	OR796456
Andrenidae	*Andrenajuliana* Wood, 2021	INV12525	IBIHM496-21	BOLD:AEO3956'	OR796882
Andrenidae	*Andrenalabialis* (Kirby, 1802)	INV12526	IBIHM497-21	BOLD:AAK0232	OR796783
Andrenidae	*Andrenalabialis* (Kirby, 1802)	INV13189	IBIHM760-21	BOLD:AAK0232	OR796394
Andrenidae	*Andrenalabiata* Fabricius, 1781	INV02352	IBIHM019-19	BOLD:ADS9137	OR796910
Andrenidae	*Andrenalabiata* Fabricius, 1781	INV02353	IBIHM020-19	BOLD:ADS9137	OR796320
Andrenidae	*Andrenalabiata* Fabricius, 1781	INV12527	IBIHM498-21	BOLD:ADS9137	OR796000
Andrenidae	*Andrenalabiata* Fabricius, 1781	INV13419	IBIHM989-21	BOLD:ADS9137	OR796244
Andrenidae	*Andrenalagopus* Latreille, 1809	INV12528	IBIHM499-21	BOLD:AAK0222	OR796638
Andrenidae	*Andrenalagopus* Latreille, 1809	INV13389	IBIHM959-21	BOLD:AAK0222	OR796802
Andrenidae	*Andrenalagopus* Latreille, 1809	INV13396	IBIHM966-21	BOLD:AAK0222	OR795990
Andrenidae	*Andrenaleptopyga* Pérez, 1895	INV13371	IBIHM941-21	BOLD:AEO3338	OR796421
Andrenidae	*Andrenaleucolippa* Pérez, 1895	INV12529	IBIHM500-21	BOLD:AEO2473'	OR796565
Andrenidae	*Andrenalimata* Smith, 1853	INV12530	IBIHM501-21	BOLD:AAE1815	OR796200
Andrenidae	*Andrenalimata* Smith, 1853	INV13386	IBIHM956-21	BOLD:AAE1815	OR796862
Andrenidae	*Andrenalimata* Smith, 1853	INV13384	IBIHM954-21	BOLD:AEX3903	OR796675
Andrenidae	*Andrenalivens* Pérez, 1895	INV12531	IBIHM502-21	BOLD:AEL4518	OR796809
Andrenidae	*Andrenalivens* Pérez, 1895	INV12532	IBIHM503-21	BOLD:AEL4518	OR796116
Andrenidae	*Andrenalongibarbis* Pérez, 1895	INV12533	IBIHM504-21	BOLD:AEO0110	OR795913
Andrenidae	*Andrenalongibarbis* Pérez, 1895	INV12534	IBIHM505-21	BOLD:AEO0110	OR796779
Andrenidae	*Andrenalongibarbis* Pérez, 1895	INV13422	IBIHM992-21	BOLD:AEO0110	OR796557
Andrenidae	*Andrenalusitania* Wood & Ortiz-Sánchez, 2022#	INV13428	IBIHM998-21	BOLD:AET5292	OR796355
Andrenidae	*Andrenaminutula* (Kirby, 1802)	INV12535	IBIHM506-21	BOLD:AAJ2143	OR796477
Andrenidae	*Andrenaminutula* (Kirby, 1802)	INV12536	IBIHM507-21	BOLD:AAJ2143	OR796060
Andrenidae	*Andrenaminutula* (Kirby, 1802)	INV12564	IBIHM535-21	BOLD:AAJ2143	OR796044
Andrenidae	*Andrenaminutula* (Kirby, 1802)	INV13191	IBIHM762-21	BOLD:AAJ2143	OR796198
Andrenidae	*Andrenaminutula* (Kirby, 1802)	INV13407	IBIHM977-21	BOLD:AAJ2143	OR796039
Andrenidae	*Andrenaminutuloides* Perkins, 1914	INV12537	IBIHM508-21	BOLD:AAE4947	OR796022
Andrenidae	*Andrenamorio* Brullé, 1832	INV12523	IBIHM494-21	BOLD:AAJ2141	OR795955
Andrenidae	*Andrenamorio* Brullé, 1832	INV13380	IBIHM950-21	BOLD:AAJ2141	OR796420
Andrenidae	*Andrenamorio* Brullé, 1832	INV13381	IBIHM951-21	BOLD:AER2061	OR796324
Andrenidae	*Andrenanana* (Kirby, 1802)	INV12538	IBIHM509-21	BOLD:AAR3413	OR796476
Andrenidae	*Andrenanana* (Kirby, 1802)	INV13404	IBIHM974-21	BOLD:AAR3413	OR796337
Andrenidae	*Andrenanana* (Kirby, 1802)	INV13406	IBIHM976-21	BOLD:AAR3413	OR795968
Andrenidae	*Andrenanigroaenea* (Kirby, 1802)	INV12539	IBIHM510-21	BOLD:AAC8068	OR796900
Andrenidae	*Andrenanigroaenea* (Kirby, 1802)	INV13173	IBIHM744-21	BOLD:AAC8068	OR796237
Andrenidae	*Andrenanigroaenea* (Kirby, 1802)	INV13186	IBIHM757-21	BOLD:AAC8068	OR796885
Andrenidae	*Andrenanigroolivacea* Dours, 1873	INV12540	IBIHM511-21	BOLD:ADZ3313	OR796223
Andrenidae	*Andrenanigroolivacea* Dours, 1873	INV13194	IBIHM765-21	BOLD:ADZ3313	OR796879
Andrenidae	*Andrenanigroolivacea* Dours, 1873	INV13195	IBIHM766-21	BOLD:ADZ3313	OR795987
Andrenidae	*Andrenanitidula* Pérez, 1903	INV12642	IBIHM1043-22	BOLD:AAJ2142	OR796203
Andrenidae	*Andrenanitidula* Pérez, 1903	INV12814	IBIHM1215-22	BOLD:AAJ2142	OR795912
Andrenidae	*Andrenaorana* Warncke, 1975	INV12542	IBIHM513-21	BOLD:AEI8573	OR796921
Andrenidae	*Andrenaorbitalis* Morawitz, 1871#	INV12543	IBIHM514-21	BOLD:AEO3428'	OR796140
Andrenidae	*Andrenaorbitalis* Morawitz, 1871#	INV13373	IBIHM943-21	BOLD:AEO3428'	OR796432
Andrenidae	*Andrenaorbitalis* Morawitz, 1871#	INV13374	IBIHM944-21	BOLD:AEO3428'	OR796307
Andrenidae	*Andrenaorbitalis* Morawitz, 1871#	INV13425	IBIHM995-21	BOLD:AEO3428'	OR796096
Andrenidae	*Andrenaovatula* (Kirby, 1802)	INV12644	IBIHM1045-22	BOLD:AAK0399	OR796018
Andrenidae	*Andrenaovatula* (Kirby, 1802)	INV13414	IBIHM984-21	BOLD:AAK0399	OR796216
Andrenidae	*Andrenaovatula* (Kirby, 1802)	INV13426	IBIHM996-21	BOLD:AAK0399	OR796108
Andrenidae	*Andrenaovatula* (Kirby, 1802)	INV13429	IBIHM999-21	BOLD:AAK0399	OR796389
Andrenidae	*Andrenaoviventris* Pérez, 1895	INV12544	IBIHM515-21	BOLD:AEO5504	OR796772
Andrenidae	*Andrenaoviventris* Pérez, 1895	INV13378	IBIHM948-21	BOLD:AEO5504	OR796443
Andrenidae	*Andrenapandellei* Pérez, 1895	INV12545	IBIHM516-21	BOLD:AEO5453	OR796629
Andrenidae	*Andrenapanurgina* De Stefani, 1889	INV12830	IBIHM1231-22	BOLD:AEM7346	OR796268
Andrenidae	*Andrenapanurgina* De Stefani, 1889	INV12843	IBIHM1244-22	BOLD:AEM7346	OR796086
Andrenidae	*Andrenapilipes* Fabricius, 1781	INV12549	IBIHM520-21	BOLD:AAF1031	OR796038
Andrenidae	*Andrenapilipes* Fabricius, 1781	INV12550	IBIHM521-21	BOLD:AAF1031	OR796288
Andrenidae	*Andrenapraecox* (Scopoli, 1763)	INV13395	IBIHM965-21	BOLD:AER0527'	OR796097
Andrenidae	*Andrenapropinqua* Schenck, 1853	INV12643	IBIHM1044-22	BOLD:AAJ2115	OR795957
Andrenidae	*Andrenapropinqua* Schenck, 1853	INV13167	IBIHM738-21	BOLD:AAJ2115	OR796428
Andrenidae	*Andrenapropinqua* Schenck, 1853	INV13417	IBIHM987-21	BOLD:AAJ2115	OR796648
Andrenidae	*Andrenapropinqua* Schenck, 1853	INV13185	IBIHM756-21	BOLD:AEN9473	OR796102
Andrenidae	*Andrenapropinqua* Schenck, 1853	INV13228	IBIHM799-21	BOLD:AEN9473	OR795997
Andrenidae	*Andrenapropinqua* Schenck, 1853	INV13410	IBIHM980-21	BOLD:AEN9473	OR796158
Andrenidae	*Andrenapropinqua* Schenck, 1853	INV13423	IBIHM993-21	BOLD:AEN9473	OR796953
Andrenidae	*Andrenapropinqua* Schenck, 1853	INV13427	IBIHM997-21	BOLD:AEN9473	OR796473
Andrenidae	*Andrenaramosa* Wood 2022#	INV12553	IBIHM524-21	BOLD:AEO3339'	OR796144
Andrenidae	*Andrenarhenana* E.Stöckhert, 1930	INV02286	IBIHM011-19	BOLD:AAN4168	OR796726
Andrenidae	*Andrenarhenana* E.Stöckhert, 1930	INV12551	IBIHM522-21	BOLD:AAN4168	OR796914
Andrenidae	*Andrenarhenana* E.Stöckhert, 1930	INV13177	IBIHM748-21	BOLD:AAN4168	OR796721
Andrenidae	*Andrenarhenana* E.Stöckhert, 1930	INV13178	IBIHM749-21	BOLD:AAN4168	OR796860
Andrenidae	*Andrenarhenana* E.Stöckhert, 1930	INV13179	IBIHM750-21	BOLD:AAN4168	OR796263
Andrenidae	*Andrenarhenana* E.Stöckhert, 1930	INV13413	IBIHM983-21	BOLD:AAN4168	OR796640
Andrenidae	*Andrenarhyssonota* Pérez, 1895	INV12552	IBIHM523-21	BOLD:AEO1135	OR796265
Andrenidae	*Andrenarhyssonota* Pérez, 1895	INV13372	IBIHM942-21	BOLD:AEO1135	OR796615
Andrenidae	*Andrenarussula* Lepeletier, 1841	INV12558	IBIHM529-21	BOLD:AAZ1205	OR795971
Andrenidae	*Andrenarussula* Lepeletier, 1841	INV13375	IBIHM945-21	BOLD:AAZ1205	OR796594
Andrenidae	*Andrenarussula* Lepeletier, 1841	INV13408	IBIHM978-21	BOLD:AAZ1205	OR795949
Andrenidae	*Andrenarussula* Lepeletier, 1841	INV12555	IBIHM526-21	BOLD:AEN8931	OR796728
Andrenidae	*Andrenarussula* Lepeletier, 1841	INV12556	IBIHM527-21	BOLD:AEN8931	OR796861
Andrenidae	*Andrenarussula* Lepeletier, 1841	INV12557	IBIHM528-21	BOLD:AEN8931	OR796074
Andrenidae	*Andrenasaxonica* E.Stöckhert, 1935	INV12636	IBIHM1037-22	BOLD:AEO3917	OR796266
Andrenidae	*Andrenaschencki* Morawitz, 1866	INV13225	IBIHM796-21	BOLD:AEO3916	OR796322
Andrenidae	*Andrenasenecionis* Pérez, 1895	INV12554	IBIHM525-21	BOLD:AEN9475	OR796568
Andrenidae	*Andrenasenecionis* Pérez, 1895	INV13187	IBIHM758-21	BOLD:AEN9475	OR796757
Andrenidae	*Andrenaspreta* Pérez, 1895	INV12560	IBIHM531-21	BOLD:ABA7470	OR796803
Andrenidae	*Andrenaspreta* Pérez, 1895	INV12561	IBIHM532-21	BOLD:ABA7470	OR796844
Andrenidae	*Andrenaspreta* Pérez, 1895	INV12562	IBIHM533-21	BOLD:ABA7470	OR796436
Andrenidae	*Andrenaspreta* Pérez, 1895	INV12565	IBIHM536-21	BOLD:ABA7470	OR796356
Andrenidae	*Andrenaspreta* Pérez, 1895	INV13190	IBIHM761-21	BOLD:ABA7470	OR796876
Andrenidae	*Andrenaspreta* Pérez, 1895	INV13415	IBIHM985-21	BOLD:ABA7470	OR796057
Andrenidae	*Andrenaspreta* Pérez, 1895	INV13416	IBIHM986-21	BOLD:ABA7470	OR796676
Andrenidae	*Andrenasuerinensis* Friese, 1884	INV12566	IBIHM537-21	BOLD:AEO0042	OR796364
Andrenidae	*Andrenasuerinensis* Friese, 1884	INV12567	IBIHM538-21	BOLD:AEO0042	OR796618
Andrenidae	*Andrenasynadelpha* Perkins, 1914	INV12568	IBIHM539-21	BOLD:ACQ8363	OR796754
Andrenidae	*Andrenasynadelpha* Perkins, 1914	INV12569	IBIHM540-21	BOLD:ACQ8363	OR796617
Andrenidae	*Andrenatenuistriata* Pérez, 1895	INV12570	IBIHM541-21	BOLD:AEO5588'	OR796070
Andrenidae	*Andrenatenuistriata* Pérez, 1895	INV13197	IBIHM768-21	BOLD:AEO5588'	OR796461
Andrenidae	*Andrenathoracica* (Fabricius, 1775)	INV12571	IBIHM542-21	BOLD:AAE1815	OR795911
Andrenidae	*Andrenathoracica* (Fabricius, 1775)	INV13159	IBIHM730-21	BOLD:AAE1815	OR796489
Andrenidae	*Andrenathoracica* (Fabricius, 1775)	INV13160	IBIHM731-21	BOLD:AAE1815	OR796023
Andrenidae	*Andrenathoracica* (Fabricius, 1775)	INV13391	IBIHM961-21	BOLD:AAE1815	OR796668
Andrenidae	*Andrenatrimmerana* (Kirby, 1802)	INV12640	IBIHM1041-22	BOLD:AAD2472	OR796778
Andrenidae	*Andrenatrimmerana* (Kirby, 1802)	INV13170	IBIHM741-21	BOLD:AAD2472	OR796441
Andrenidae	*Andrenatruncatilabris* Morawitz, 1877	INV12641	IBIHM1042-22	BOLD:AEO1242	OR795998
Andrenidae	*Andrenavariabilis* Smith, 1853	INV12572	IBIHM543-21	BOLD:AEK5331	OR796637
Andrenidae	*Andrenaventricosa* Dours, 1873	INV12573	IBIHM544-21	BOLD:AEK6031	OR796830
Andrenidae	*Andrenaverticalis* Pérez, 1895	INV12650	IBIHM1051-22	BOLD:AEN0289	OR796479
Andrenidae	*Andrenavetula* Lepeletier, 1841	INV12831	IBIHM1232-22	BOLD:AEO0268	OR796124
Andrenidae	*Andrenavillipes* Pérez, 1895	INV12574	IBIHM545-21	BOLD:AEO1830	OR795991
Andrenidae	*Andrenavillipes* Pérez, 1895	INV12819	IBIHM1220-22	BOLD:AEO1830	OR796796
Andrenidae	*Andrenavillipes* Pérez, 1895	INV12820	IBIHM1221-22	BOLD:AEO1830	OR796205
Andrenidae	*Andrenavulcana* Dours, 1873	INV12815	IBIHM1216-22	BOLD:AES1212'	OR796672
Andrenidae	*Andrenavulcana* Dours, 1873	INV12816	IBIHM1217-22	BOLD:AES1212'	OR796049
Andrenidae	*Andrenawilkella* (Kirby, 1802)	INV12576	IBIHM547-21	BOLD:AAA8959	OR796493
Andrenidae	*Flavipanurgusgranadensis* (Warncke, 1987)	INV12377	IBIHM448-21	BOLD:AEO4846	OR796833
Andrenidae	*Flavipanurgusgranadensis* (Warncke, 1987)	INV12376	IBIHM447-21	BOLD:AEO4847'	OR796866
Andrenidae	*Flavipanurguskastiliensis* (Warncke, 1985)#	INV13322	IBIHM892-21	BOLD:AEO4911	OR796378
Andrenidae	*Halopanurgusbaldocki* (Wood & Cross, 2017)#	INV12378	IBIHM449-21	BOLD:AEO5533'	OR796744
Andrenidae	*Melitturgacaudata* Pérez, 1879#	INV12364	IBIHM435-21	BOLD:AEO3399'	OR796657
Andrenidae	*Panurginusalbopilosus* (Lucas, 1849)#	INV12372	IBIHM443-21	BOLD:AEN9481'	OR796143
Andrenidae	*Panurginusalbopilosus* (Lucas, 1849)#	INV12373	IBIHM444-21	BOLD:AEN9481'	OR796546
Andrenidae	*Panurginusalbopilosus* (Lucas, 1849)#	INV12374	IBIHM445-21	BOLD:AEN9481'	OR796113
Andrenidae	*Panurgusbanksianus* (Kirby, 1802)	INV12379	IBIHM450-21	BOLD:AAO8748	OR796587
Andrenidae	*Panurgusbanksianus* (Kirby, 1802)	INV13324	IBIHM894-21	BOLD:AAO8748	OR796543
Andrenidae	*Panurguscalcaratus* (Scopoli, 1763)	INV01510	IBIHM025-19	BOLD:ADD7208	OR796789
Andrenidae	*Panurguscalcaratus* (Scopoli, 1763)	INV01526	IBIHM027-19	BOLD:ADD7208	OR796501
Andrenidae	*Panurguscalcaratus* (Scopoli, 1763)	INV01530	IBIHM028-19	BOLD:ADD7208	OR796361
Andrenidae	*Panurguscalcaratus* (Scopoli, 1763)	INV01540	IBIHM029-19	BOLD:ADD7208	OR796025
Andrenidae	*Panurguscalcaratus* (Scopoli, 1763)	INV01550	IBIHM031-19	BOLD:ADD7208	OR796209
Andrenidae	*Panurguscalcaratus* (Scopoli, 1763)	INV01557	IBIHM033-19	BOLD:ADD7208	OR796252
Andrenidae	*Panurguscalcaratus* (Scopoli, 1763)	INV01567	IBIHM035-19	BOLD:ADD7208	OR796169
Andrenidae	*Panurguscalcaratus* (Scopoli, 1763)	INV12380	IBIHM451-21	BOLD:ADD7208	OR796931
Andrenidae	*Panurguscalcaratus* (Scopoli, 1763)	INV13323	IBIHM893-21	BOLD:ADD7208	OR796698
Andrenidae	*Panurguscanescens* Latreille, 1811#	INV12798	IBIHM1199-22	BOLD:AES8898'	OR795952
Andrenidae	*Panurguscanescens* Latreille, 1811#	INV12799	IBIHM1200-22	BOLD:AES8898'	OR795986
Andrenidae	*Panurguscephalotes* Latreille, 1811#	INV12375	IBIHM446-21	BOLD:AEO4429'	OR795946
Andrenidae	*Panurguscephalotes* Latreille, 1811#	INV13200	IBIHM771-21	BOLD:AEO4429'	OR796316
Andrenidae	*Panurguscephalotes* Latreille, 1811#	INV13201	IBIHM772-21	BOLD:AEO4429'	OR796106
Andrenidae	*Panurguscephalotes* Latreille, 1811#	INV13202	IBIHM773-21	BOLD:AEO4429'	OR796537
Andrenidae	*Panurguscephalotes* Latreille, 1811#	INV13328	IBIHM898-21	BOLD:AEO4429'	OR796155
Andrenidae	*Panurgusdargius* Warncke, 1972#	INV12369	IBIHM440-21	BOLD:AEO3268'	OR796741
Andrenidae	*Panurgusperezi* Saunders, 1882#	INV12382	IBIHM453-21	BOLD:AEO3269'	OR796449
Andrenidae	*Panurgusperezi* Saunders, 1882#	INV13203	IBIHM774-21	BOLD:AEO3269'	OR796573
Andrenidae	*Panurgusperezi* Saunders, 1882#	INV13204	IBIHM775-21	BOLD:AEO3269'	OR795992
Andrenidae	*Panurgusperezi* Saunders, 1882#	INV13205	IBIHM776-21	BOLD:AEO3269'	OR796150
Apidae	*Amegillaalbigena* (Lepeletier, 1841)	INV12150	IBIHM221-21	BOLD:AEL4482	OR795951
Apidae	*Amegillaalbigena* (Lepeletier, 1841)	INV12149	IBIHM220-21	BOLD:AEO2968	OR796701
Apidae	*Amegillaalbigena* (Lepeletier, 1841)	INV13344	IBIHM914-21	BOLD:AEO2968	OR796696
Apidae	*Amegillafasciata* (Fabricius, 1775)	INV12151	IBIHM222-21	BOLD:AEN9315	OR795963
Apidae	*Amegillaquadrifasciata* (de Villers, 1789)	INV12152	IBIHM223-21	BOLD:ABX1552	OR796854
Apidae	*Amegillasavignyi* (Lepeletier, 1841)	INV12468	IBIHM1321-22	BOLD:AEU2956	OR796202
Apidae	*Ammobatesmuticus* Spinola, 1843	INV12013	IBIHM084-21	BOLD:AEO2733	OR796197
Apidae	*Ammobatesmuticus* Spinola, 1843	INV12014	IBIHM085-21	BOLD:AEO2733	OR796670
Apidae	*Ammobatesmuticus* Spinola, 1843	INV12015	IBIHM086-21	BOLD:AEO2733	OR796112
Apidae	*Ammobatespunctatus* (Fabricius, 1804)	INV12030	IBIHM101-21	BOLD:AAC7051	OR796870
Apidae	*Ammobatoidesscriptus* (Gerstäcker, 1869)	INV12028	IBIHM099-21	BOLD:AEO4234	OR796004
Apidae	*Anthophoraaestivalis* (Panzer, 1801)	INV12129	IBIHM200-21	BOLD:AAK2880	OR796008
Apidae	*Anthophoraagama* Radoszkowski, 1869	INV12130	IBIHM201-21	BOLD:AEO0218	OR796697
Apidae	*Anthophoraatriceps* Pérez, 1879	INV12131	IBIHM202-21	BOLD:AEO3578	OR796507
Apidae	*Anthophoraatroalba* Lepeletier, 1841	INV12132	IBIHM203-21	BOLD:AER1825	OR796230
Apidae	*Anthophorabalneorum* Lepeletier, 1841	INV12133	IBIHM204-21	BOLD:AEN8225'	OR796118
Apidae	*Anthophorabimaculata* (Panzer, 1798)	INV12134	IBIHM205-21	BOLD:AAJ2426	OR796067
Apidae	*Anthophorabimaculata* (Panzer, 1798)	INV13332	IBIHM902-21	BOLD:AAJ2426	OR796528
Apidae	*Anthophorabimaculata* (Panzer, 1798)	INV15650	IBIHY001-22	BOLD:AAJ2426	OR796881
Apidae	*Anthophorabimaculata* (Panzer, 1798)	INV15661	IBIHY005-22	BOLD:AAJ2426	OR796319
Apidae	*Anthophorabimaculata* (Panzer, 1798)	INV15665	IBIHY009-22	BOLD:AAJ2426	OR795923
Apidae	*Anthophorabimaculata* (Panzer, 1798)	INV15667	IBIHY010-22	BOLD:AAJ2426	OR796624
Apidae	*Anthophorabimaculata* (Panzer, 1798)	INV15681	IBIHY023-22	BOLD:AAJ2426	OR796782
Apidae	*Anthophoracrassipes* Lepeletier, 1841	INV12135	IBIHM206-21	BOLD:AEN9016	OR796093
Apidae	*Anthophoracrinipes* Smith, 1854	INV12136	IBIHM207-21	BOLD:ADK6129	OR796500
Apidae	*Anthophoracrinipes* Smith, 1854	INV13331	IBIHM901-21	BOLD:ADK6129	OR796452
Apidae	*Anthophoradispar* Lepeletier, 1841	INV12137	IBIHM208-21	BOLD:AEO5509	OR796028
Apidae	*Anthophoradispar* Lepeletier, 1841	INV13330	IBIHM900-21	BOLD:AEO5509	OR795975
Apidae	*Anthophorafemorata* (Olivier, 1789)	INV12142	IBIHM213-21	BOLD:ADD5374	OR796890
Apidae	*Anthophorafulvitarsis* Brullé, 1832	INV12138	IBIHM209-21	BOLD:AEO0884	OR796359
Apidae	*Anthophorafulvodimidiata* Dours, 1869	INV12139	IBIHM210-21	BOLD:AEO1252	OR796163
Apidae	*Anthophorafurcata* (Panzer, 1798)	INV12141	IBIHM212-21	BOLD:AAC5304	OR796405
Apidae	*Anthophoragallica* (Dalla Torre & Friese, 1895)	INV12140	IBIHM211-21	BOLD:AEO4497	OR796718
Apidae	*Anthophoragallica* (Dalla Torre & Friese, 1895)	INV12143	IBIHM214-21	BOLD:AEO4497	OR796370
Apidae	*Anthophorahispanica* (Fabricius, 1787)	INV12153	IBIHM224-21	BOLD:AEO5858'	OR796469
Apidae	*Anthophorahispanica* (Fabricius, 1787)	INV13329	IBIHM899-21	BOLD:AEO5858'	OR796257
Apidae	*Anthophoraleucophaea* Pérez, 1879	INV12469	IBIHM1322-22	BOLD:AEN6387	OR796621
Apidae	*Anthophoramucida* Gribodo, 1873	INV12144	IBIHM215-21	BOLD:AEO0770	OR796368
Apidae	*Anthophoraplumipes* (Pallas, 1772)	INV12146	IBIHM217-21	BOLD:AEN8725	OR796306
Apidae	*Anthophoraplumipes* (Pallas, 1772)	INV13015	IBIHM586-21	BOLD:AEN8725	OR796367
Apidae	*Anthophoraplumipes* (Pallas, 1772)	INV13013	IBIHM584-21	BOLD:AEO1358	OR796547
Apidae	*Anthophorapodagra* Lepeletier, 1841	INV12145	IBIHM216-21	BOLD:AEO2867	OR796574
Apidae	*Anthophoraretusa* (Linnaeus, 1758)	INV12147	IBIHM218-21	BOLD:ACD9731	OR796280
Apidae	*Anthophoraretusa* (Linnaeus, 1758)	INV13014	IBIHM585-21	BOLD:ACD9731	OR796327
Apidae	*Anthophoraretusa* (Linnaeus, 1758)	INV13016	IBIHM587-21	BOLD:ACD9731	OR796227
Apidae	*Anthophorarobusta* (Klug, 1845)	INV12148	IBIHM219-21	BOLD:AEO3449'	OR796191
Apidae	*Apismellifera* Linnaeus, 1758	INV01913	IBIHM040-19	BOLD:AAA2326	OR796894
Apidae	*Apismellifera* Linnaeus, 1758	INV04881	IBIHM061-19	BOLD:AAA2326	OR796077
Apidae	*Apismellifera* Linnaeus, 1758	INV13041	IBIHM612-21	BOLD:AAA2326	OR796534
Apidae	*Apismellifera* Linnaeus, 1758	INV13042	IBIHM613-21	BOLD:AAA2326	OR796172
Apidae	*Bombusbarbutellus* (Kirby, 1802)	INV12154	IBIHM225-21	BOLD:AAF7051	OR795945
Apidae	*Bombuscampestris* (Panzer, 1801)	INV12155	IBIHM226-21	BOLD:AAD8221	OR796002
Apidae	*Bombushortorum* (Linnaeus, 1761)	INV08813	IBIHM065-20	BOLD:AAD2566	OR796294
Apidae	*Bombushortorum* (Linnaeus, 1761)	INV13005	IBIHM576-21	BOLD:AAD2566	OR795929
Apidae	*Bombushortorum* (Linnaeus, 1761)	INV13006	IBIHM577-21	BOLD:AAD2566	OR796835
Apidae	*Bombushortorum* (Linnaeus, 1761)	INV15676	IBIHY019-22	BOLD:AAD2566	OR796174
Apidae	*Bombushumilis* Illiger, 1806	INV12470	IBIHM1323-22	BOLD:ABY7210	OR796485
Apidae	*Bombuslapidarius* (Linnaeus, 1758)	INV12175	IBIHM246-21	BOLD:AEF7156	OR796287
Apidae	*Bombusmuscorum* (Linnaeus, 1758)	INV12472	IBIHM1325-22	BOLD:AAD8159	OR796064
Apidae	*Bombusmuscorum* (Linnaeus, 1758)	INV12473	IBIHM1326-22	BOLD:AAD8159	OR796795
Apidae	*Bombuspascuorum* (Scopoli, 1763)	INV12156	IBIHM227-21	BOLD:AAC4378	OR796627
Apidae	*Bombuspascuorum* (Scopoli, 1763)	INV12159	IBIHM230-21	BOLD:AAC4378	OR795916
Apidae	*Bombuspascuorum* (Scopoli, 1763)	INV13007	IBIHM578-21	BOLD:AAC4378	OR796775
Apidae	*Bombuspascuorum* (Scopoli, 1763)	INV13008	IBIHM579-21	BOLD:AAC4378	OR796317
Apidae	*Bombuspascuorum* (Scopoli, 1763)	INV13009	IBIHM580-21	BOLD:AAC4378	OR796199
Apidae	*Bombuspascuorum* (Scopoli, 1763)	INV13010	IBIHM581-21	BOLD:AAC4378	OR796654
Apidae	*Bombuspascuorum* (Scopoli, 1763)	INV13038	IBIHM609-21	BOLD:AAC4378	OR796132
Apidae	*Bombuspascuorum* (Scopoli, 1763)	INV13039	IBIHM610-21	BOLD:AAC4378	OR796646
Apidae	*Bombuspratorum* (Linnaeus, 1761)	INV12157	IBIHM228-21	BOLD:AAD4735	OR796816
Apidae	*Bombuspratorum* (Linnaeus, 1761)	INV13001	IBIHM572-21	BOLD:AAD4735	OR796607
Apidae	*Bombusruderatus* (Fabricius, 1775)	INV02336	IBIHM018-19	BOLD:AAJ7737	OR795958
Apidae	*Bombusruderatus* (Fabricius, 1775)	INV12158	IBIHM229-21	BOLD:AAJ7737	OR796611
Apidae	*Bombussylvarum* (Linnaeus, 1761)	INV12177	IBIHM248-21	BOLD:AAD2551	OR796379
Apidae	*Bombusterrestris* (Linnaeus, 1758)	INV12471	IBIHM1324-22	BOLD:AAB1062	OR796774
Apidae	*Bombusterrestris* (Linnaeus, 1758)	INV13000	IBIHM571-21	BOLD:AAB1062	OR796798
Apidae	*Bombusterrestris* (Linnaeus, 1758)	INV13002	IBIHM573-21	BOLD:AAB1062	OR796260
Apidae	*Bombusterrestris* (Linnaeus, 1758)	INV13003	IBIHM574-21	BOLD:AAB1062	OR796592
Apidae	*Bombusterrestris* (Linnaeus, 1758)	INV13004	IBIHM575-21	BOLD:AAB1062	OR796269
Apidae	*Bombusterrestris* (Linnaeus, 1758)	INV13011	IBIHM582-21	BOLD:AAB1062	OR796107
Apidae	*Bombusterrestris* (Linnaeus, 1758)	INV13234	IBIHM804-21	BOLD:AAB1062	OR796239
Apidae	*Bombusvestalis* (Geoffroy, 1785)	INV09330	IBIHM070-20	BOLD:AAI8745	OR796818
Apidae	*Bombusvestalis* (Geoffroy, 1785)	INV12160	IBIHM231-21	BOLD:AAI8745	OR796909
Apidae	*Bombusvestalis* (Geoffroy, 1785)	INV13233	IBIHM803-21	BOLD:AAI8745	OR796815
Apidae	*Ceratinacallosa* (Fabricius, 1794)#	INV12162	IBIHM233-21	BOLD:AEN0370	OR796945
Apidae	*Ceratinachalcites* Germar, 1839	INV12165	IBIHM236-21	BOLD:ACM3088	OR796839
Apidae	*Ceratinachalcites* Germar, 1839	INV13258	IBIHM828-21	BOLD:ACM3088	OR796036
Apidae	*Ceratinachalybea* Chevrier, 1872	INV12164	IBIHM235-21	BOLD:AAW6323	OR795927
Apidae	*Ceratinacucurbitina* (Rossi, 1792)	INV12163	IBIHM234-21	BOLD:AAO0285	OR796251
Apidae	*Ceratinacucurbitina* (Rossi, 1792)	INV13037	IBIHM608-21	BOLD:AAO0285	OR796824
Apidae	*Ceratinacucurbitina* (Rossi, 1792)	INV13214	IBIHM785-21	BOLD:AAO0285	OR796954
Apidae	*Ceratinacucurbitina* (Rossi, 1792)	INV15669	IBIHY012-22	BOLD:AAO0285	OR796797
Apidae	*Ceratinacyanea* (Kirby, 1802)	INV02339	IBIHM046-19	BOLD:AAE9812	OR796425
Apidae	*Ceratinacyanea* (Kirby, 1802)	INV12170	IBIHM241-21	BOLD:AAE9812	OR796723
Apidae	*Ceratinacyanea* (Kirby, 1802)	INV13034	IBIHM605-21	BOLD:AAE9812	OR796828
Apidae	*Ceratinacyanea* (Kirby, 1802)	INV13215	IBIHM786-21	BOLD:AAE9812	OR796552
Apidae	*Ceratinacyanea* (Kirby, 1802)	INV13216	IBIHM787-21	BOLD:AAE9812	OR796259
Apidae	*Ceratinacyanea* (Kirby, 1802)	INV13252	IBIHM822-21	BOLD:AAE9812	OR796823
Apidae	*Ceratinacyanea* (Kirby, 1802)	INV13254	IBIHM824-21	BOLD:AAE9812	OR795924
Apidae	*Ceratinadallatorreana* Friese, 1896	INV12169	IBIHM240-21	BOLD:ADF8180	OR796186
Apidae	*Ceratinadallatorreana* Friese, 1896	INV13035	IBIHM606-21	BOLD:ADF8180	OR796583
Apidae	*Ceratinadallatorreana* Friese, 1896	INV13255	IBIHM825-21	BOLD:ADF8180	OR796684
Apidae	*Ceratinagravidula* Gerstäcker, 1869	INV12161	IBIHM232-21	BOLD:AEA2549	OR796616
Apidae	*Ceratinamocsaryi* Friese, 1896	INV12167	IBIHM238-21	BOLD:AEO3844	OR796766
Apidae	*Ceratinamocsaryi* Friese, 1896	INV13253	IBIHM823-21	BOLD:AEO3844	OR796608
Apidae	*Ceratinamocsaryi* Friese, 1896	INV13257	IBIHM827-21	BOLD:AEO3844	OR796376
Apidae	*Ceratinanigrolabiata* Friese, 1896	INV12474	IBIHM1327-22	BOLD:ACG0777	OR796448
Apidae	*Ceratinanigrolabiata* Friese, 1896	INV12475	IBIHM1328-22	BOLD:ACG0777	OR796678
Apidae	*Ceratinanigrolabiata* Friese, 1896	INV13256	IBIHM826-21	BOLD:ACG0777	OR796868
Apidae	*Ceratinaparvula* Smith, 1854	INV12166	IBIHM237-21	BOLD:AEO2595'	OR796596
Apidae	*Ceratinasaundersi* Daly, 1983#	INV12176	IBIHM247-21	BOLD:AEO3675'	OR796877
Apidae	*Epeoluscrucigermarginatus* Bischoff, 1930	INV12012	IBIHM083-21	BOLD:AAN3663	OR796946
Apidae	*Epeoluscruciger* (Panzer, 1799)	INV12462	IBIHM1315-22	BOLD:AAN3663	OR796164
Apidae	*Epeolusintermedius* Pérez, 1884	INV12464	IBIHM1317-22	BOLD:AAJ0611	OR796091
Apidae	*Epeolusintermedius* Pérez, 1884	INV12467	IBIHM1320-22	BOLD:AAJ0611	OR795988
Apidae	*Epeolusjulliani* Pérez, 1884	INV12463	IBIHM1316-22	BOLD:ACD1277	OR796659
Apidae	*Epeolusvariegatus* (Linnaeus, 1758)	INV12466	IBIHM1319-22	BOLD:AAJ0611	OR796525
Apidae	*Eucerabarbiventris* Pérez, 1902#	INV12100	IBIHM171-21	BOLD:AEN2742	OR796562
Apidae	*Eucerabarbiventris* Pérez, 1902#	INV13239	IBIHM809-21	BOLD:AEN2742	OR796912
Apidae	*Eucerachrysopyga* Pérez, 1879	INV12114	IBIHM185-21	BOLD:ABV4745	OR796487
Apidae	*Eucerachrysopyga* Pérez, 1879	INV13017	IBIHM588-21	BOLD:ABV4745	OR796235
Apidae	*Eucerachrysopyga* Pérez, 1879	INV13021	IBIHM592-21	BOLD:ABV4745	OR796079
Apidae	*Euceracollaris* Dours, 1873	INV12104	IBIHM175-21	BOLD:AEO1485'	OR796413
Apidae	*Euceracollaris* Dours, 1873	INV13237	IBIHM807-21	BOLD:AEO1485'	OR796855
Apidae	*Euceradalmatica* Lepeletier, 1841	INV13235	IBIHM805-21	BOLD:ADE3334	OR795980
Apidae	*Euceradalmatica* Lepeletier, 1841	INV13236	IBIHM806-21	BOLD:ADE3334	OR796856
Apidae	*Euceraelongatula* Vachal, 1907	INV12108	IBIHM179-21	BOLD:ABU9563	OR796076
Apidae	*Euceraelongatula* Vachal, 1907	INV12109	IBIHM180-21	BOLD:ABU9563	OR796825
Apidae	*Euceraelongatula* Vachal, 1907	INV13241	IBIHM811-21	BOLD:ABU9563	OR796516
Apidae	*Euceraelongatula* Vachal, 1907	INV13242	IBIHM812-21	BOLD:ABU9563	OR796786
Apidae	*Euceraeucnemidea* Dours, 1873	INV12110	IBIHM181-21	BOLD:AEO0071'	OR796893
Apidae	*Euceraeucnemidea* Dours, 1873	INV12111	IBIHM182-21	BOLD:AEO0071'	OR796875
Apidae	*Eucerahispana* Lepeletier, 1841#	INV12116	IBIHM187-21	BOLD:AEO1591'	OR796391
Apidae	*Eucerahungarica* Friese, 1895	INV12099	IBIHM170-21	BOLD:AAL4610	OR796898
Apidae	*Euceraiberica* (Dusmet y Alonso 1926)	INV13251	IBIHM821-21	BOLD:AER1385'	OR795982
Apidae	*Eucerainterrupta* Bär, 1850	INV12118	IBIHM189-21	BOLD:ABZ4791	OR796865
Apidae	*Euceralongicornis* (Linnaeus, 1758)	INV12115	IBIHM186-21	BOLD:ABZ4790	OR796805
Apidae	*Euceralongicornis* (Linnaeus, 1758)	INV12482	IBIHM1335-22	BOLD:ABZ4790	OR796183
Apidae	*Euceralongicornis* (Linnaeus, 1758)	INV12483	IBIHM1336-22	BOLD:ABZ4790	OR796957
Apidae	*Euceralongicornis* (Linnaeus, 1758)	INV13238	IBIHM808-21	BOLD:ABZ4790	OR796431
Apidae	*Euceranigrescens* Pérez, 1879	INV12105	IBIHM176-21	BOLD:ABZ2397	OR796395
Apidae	*Euceranigrescens* Pérez, 1879	INV13240	IBIHM810-21	BOLD:ABZ2397	OR796255
Apidae	*Euceranigrescens* Pérez, 1879	INV13246	IBIHM816-21	BOLD:ABZ2397	OR795989
Apidae	*Euceranigrescens* Pérez, 1879	INV13248	IBIHM818-21	BOLD:ABZ2397	OR796872
Apidae	*Euceranigrescens* Pérez, 1879	INV13250	IBIHM820-21	BOLD:ABZ2397	OR796333
Apidae	*Euceranigrifacies* Lepeletier, 1841	INV12120	IBIHM191-21	BOLD:AER1612'	OR795899
Apidae	*Euceranigrilabris* Lepeletier, 1841	INV12121	IBIHM192-21	BOLD:AEM9739	OR796302
Apidae	*Euceranigrilabris* Lepeletier, 1841	INV13019	IBIHM590-21	BOLD:AEM9739	OR796072
Apidae	*Euceranigrilabris* Lepeletier, 1841	INV13020	IBIHM591-21	BOLD:AEM9739	OR795907
Apidae	*Euceranotata* Lepeletier, 1841#	INV12122	IBIHM193-21	BOLD:AEN4764	OR796709
Apidae	*Euceranotata* Lepeletier, 1841#	INV13244	IBIHM814-21	BOLD:AEN4764	OR796384
Apidae	*Euceranotata* Lepeletier, 1841#	INV13245	IBIHM815-21	BOLD:AEN4764	OR796357
Apidae	*Euceranumida* Lepeletier, 1841	INV12123	IBIHM194-21	BOLD:AEA1287	OR796729
Apidae	*Euceraproxima* Morawitz, 1875	INV12832	IBIHM1233-22	BOLD:AAI4916	OR796770
Apidae	*Euceraproxima* Morawitz, 1875	INV12833	IBIHM1234-22	BOLD:AAI4916	OR796908
Apidae	*Eucerapunctatissima* Pérez, 1895#	INV12834	IBIHM1235-22	BOLD:AET2033'	OR796940
Apidae	*Eucerapunctatissima* Pérez, 1895#	INV12835	IBIHM1236-22	BOLD:AET2033'	OR795939
Apidae	*Eucerarufa* (Lepeletier, 1841)	INV12124	IBIHM195-21	BOLD:ADZ9496	OR796720
Apidae	*Euceraruficollis* (Brullé, 1832)	INV12098	IBIHM169-21	BOLD:AEM7547	OR796148
Apidae	*Eucerataurica* Morawitz, 1870	INV12126	IBIHM197-21	BOLD:AEK9612	OR796182
Apidae	*Euceratricincta* Erichson, 1835#	INV12127	IBIHM198-21	BOLD:AEO1994'	OR796080
Apidae	*Melectaalbifrons* (Forster, 1771)	INV12002	IBIHM073-21	BOLD:ACR2895	OR796340
Apidae	*Melectaalbifrons* (Forster, 1771)	INV12003	IBIHM074-21	BOLD:ACR2895	OR796141
Apidae	*Melectafestiva* Lieftinck, 1980#	INV13336	IBIHM906-21		OR796751
Apidae	*Melecta* sp.	INV12001	IBIHM072-21	BOLD:AEO2855	OR796791
Apidae	*Nomadaagrestis* Fabricius, 1787	INV12035	IBIHM106-21	BOLD:AEN9589	OR796773
Apidae	*Nomadaagrestis* Fabricius, 1787	INV13265	IBIHM835-21	BOLD:AEN9589	OR796836
Apidae	*Nomadabasalis* Herrich-Schäffer, 1839	INV13270	IBIHM840-21	BOLD:AEK6178	OR796576
Apidae	*Nomadabasalis* Herrich-Schäffer, 1839	INV12036	IBIHM107-21	BOLD:AEN3462	OR796691
Apidae	*Nomadabasalis* Herrich-Schäffer, 1839	INV12037	IBIHM108-21	BOLD:AEO4155'	OR796398
Apidae	*Nomadabasalis* Herrich-Schäffer, 1839	INV12038	IBIHM109-21	BOLD:AEO4155'	OR796767
Apidae	*Nomadabasalis* Herrich-Schäffer, 1839	INV12039	IBIHM110-21	BOLD:AEO4155'	OR795917
Apidae	*Nomadabeaumonti* Schwarz, 1967	INV12041	IBIHM112-21	BOLD:AEO4341	OR796899
Apidae	*Nomadabifasciata* Olivier, 1811	INV12040	IBIHM111-21	BOLD:AAE5755	OR795993
Apidae	*Nomadabifasciata* Olivier, 1811	INV12054	IBIHM125-21	BOLD:AAE5755	OR796048
Apidae	*Nomadabifasciata* Olivier, 1811	INV12055	IBIHM126-21	BOLD:AAE5755	OR796033
Apidae	*Nomadabifasciata* Olivier, 1811	INV13025	IBIHM596-21	BOLD:AAE5755	OR796641
Apidae	*Nomadabluethgeni* Stoeckhert, 1943	INV12608	IBIHM1009-22	BOLD:AES2302	OR796419
Apidae	*Nomadabluethgeni* Stoeckhert, 1943	INV12609	IBIHM1010-22	BOLD:AES2302	OR796527
Apidae	*Nomadacarnifex* Mocsáry, 1883	INV12042	IBIHM113-21	BOLD:AEO0078	OR796582
Apidae	*Nomadacarnifex* Mocsáry, 1883	INV12043	IBIHM114-21	BOLD:AEO0078	OR796575
Apidae	*Nomadaconjungens* Herrich-Schäffer, 1839	INV12606	IBIHM1007-22	BOLD:AER6266'	OR796630
Apidae	*Nomadaconjungens* Herrich-Schäffer, 1839	INV13024	IBIHM595-21	BOLD:AER6266'	OR796943
Apidae	*Nomadacorcyraea* Schmiedeknecht, 1882#	INV12845	IBIHM1246-22	BOLD:AEO6156	OR796137
Apidae	*Nomadacristata* Pérez, 1896#	INV12044	IBIHM115-21	BOLD:AEO1919'	OR795922
Apidae	*Nomadadira* Schmiedeknecht, 1882#	INV12045	IBIHM116-21	BOLD:AEN3179	OR795964
Apidae	*Nomadadiscrepans* Schmiedeknecht, 1882	INV12048	IBIHM119-21	BOLD:AEN8935'	OR796695
Apidae	*Nomadadiscrepans* Schmiedeknecht, 1882	INV13259	IBIHM829-21	BOLD:AEN8935'	OR796009
Apidae	*Nomadadiscrepans* Schmiedeknecht, 1882	INV13260	IBIHM830-21	BOLD:AEN8935'	OR796323
Apidae	*Nomadadistinguenda* Morawitz, 1874	INV13023	IBIHM594-21	BOLD:ACY0250	OR796567
Apidae	*Nomadadistinguenda* Morawitz, 1874	INV13027	IBIHM598-21	BOLD:ACY0250	OR796090
Apidae	*Nomadadistinguenda* Morawitz, 1874	INV12602	IBIHM1003-22	BOLD:AET5764'	OR796083
Apidae	*Nomadaduplex* Smith, 1854#	INV12049	IBIHM120-21	BOLD:AEO0073'	OR796114
Apidae	*Nomadaduplex* Smith, 1854#	INV13264	IBIHM834-21	BOLD:AEO0073'	OR796760
Apidae	*Nomadafallax* Pérez, 1913	INV12611	IBIHM1012-22	BOLD:AEW3802'	OR796010
Apidae	*Nomadafallax* Pérez, 1913	INV12612	IBIHM1013-22	BOLD:AEW3802'	OR796435
Apidae	*Nomadafallax* Pérez, 1913	INV12844	IBIHM1245-22	BOLD:AEW3802'	OR796400
Apidae	*Nomadafallax* Pérez, 1913	INV13268	IBIHM838-21	BOLD:AEW3802'	OR796892
Apidae	*Nomadafemoralis* Morawitz, 1869	INV12052	IBIHM123-21	BOLD:AAI2830	OR796271
Apidae	*Nomadafemoralis* Morawitz, 1869	INV12053	IBIHM124-21	BOLD:AEO6156	OR796559
Apidae	*Nomadafemoralis* Morawitz, 1869	INV13028	IBIHM599-21	BOLD:AEO6156	OR796372
Apidae	*Nomadafenestrata* Lepeletier, 1841#	INV12837	IBIHM1238-22	BOLD:AER8574'	OR796254
Apidae	*Nomadafenestrata* Lepeletier, 1841#	INV12838	IBIHM1239-22	BOLD:AER8574'	OR796509
Apidae	*Nomadaflavoguttata* (Kirby, 1802)	INV12046	IBIHM117-21	BOLD:AAD4959	OR796305
Apidae	*Nomadaflavoguttata* (Kirby, 1802)	INV12047	IBIHM118-21	BOLD:AAD4959	OR796404
Apidae	*Nomadaflavoguttata* (Kirby, 1802)	INV13033	IBIHM604-21	BOLD:AAD4959	OR796386
Apidae	*Nomadaflavoguttata* (Kirby, 1802)	INV13209	IBIHM780-21	BOLD:AAD4959	OR796192
Apidae	*Nomadafulvicornis* Fabricius, 1793	INV12056	IBIHM127-21	BOLD:ACE0147	OR796635
Apidae	*Nomadafulvicornis* Fabricius, 1793	INV12057	IBIHM128-21	BOLD:ACE0147	OR796850
Apidae	*Nomadafulvicornis* Fabricius, 1793	INV13207	IBIHM778-21	BOLD:ACE0147	OR795935
Apidae	*Nomadafuscicornis* Nylander, 1848	INV12601	IBIHM1002-22	BOLD:AAF3571	OR795956
Apidae	*Nomadaglaucopis* Pérez, 1890	INV13263	IBIHM833-21	BOLD:AEJ1237	OR795970
Apidae	*Nomadaglaucopis* Pérez, 1890	INV12058	IBIHM129-21	BOLD:AEO3423	OR796246
Apidae	*Nomadaglaucopis* Pérez, 1890	INV12059	IBIHM130-21	BOLD:AEO3423	OR796282
Apidae	*Nomadaglaucopis* Pérez, 1890	INV12078	IBIHM149-21	BOLD:AEO3423	OR795948
Apidae	*Nomadaglaucopis* Pérez, 1890	INV12603	IBIHM1004-22	BOLD:AEO3423	OR796184
Apidae	*Nomadagoodeniana* (Kirby, 1802)	INV13026	IBIHM597-21	BOLD:AEB4149	OR796347
Apidae	*Nomadahispanica* Dusmet y Alonso, 1913#	INV12060	IBIHM131-21	BOLD:AAE5753	OR796030
Apidae	*Nomadahispanica* Dusmet y Alonso, 1913#	INV13030	IBIHM601-21	BOLD:AAE5753	OR795915
Apidae	*Nomadaillustris* Schmiedeknecht, 1882	INV12061	IBIHM132-21	BOLD:AEN8381	OR796081
Apidae	*Nomadaillustris* Schmiedeknecht, 1882	INV12062	IBIHM133-21	BOLD:AEN8381	OR796134
Apidae	*Nomadaillustris* Schmiedeknecht, 1882	INV12600	IBIHM1001-22	BOLD:AEN8381	OR796058
Apidae	*Nomadainsignipes* Schmiedeknecht, 1882#	INV12063	IBIHM134-21	BOLD:AEL5320	OR796742
Apidae	*Nomadainsignipes* Schmiedeknecht, 1882#	INV12064	IBIHM135-21	BOLD:AEL5320	OR796730
Apidae	*Nomadaintegra* Brullé, 1832	INV12065	IBIHM136-21	BOLD:ABZ1320	OR796129
Apidae	*Nomadaintegra* Brullé, 1832	INV12095	IBIHM166-21	BOLD:AEN8926	OR796937
Apidae	*Nomadaintegra* Brullé, 1832	INV12096	IBIHM167-21	BOLD:AEN8926	OR796270
Apidae	*Nomadaintegra* Brullé, 1832	INV12097	IBIHM168-21	BOLD:AEN8926	OR796201
Apidae	*Nomadaintegra* Brullé, 1832	INV13266	IBIHM836-21	BOLD:AEN8926	OR796275
Apidae	*Nomadaintegra* Brullé, 1832	INV12066	IBIHM137-21	BOLD:AEO3904'	OR796826
Apidae	*Nomadakohli* Schmiedeknecht, 1882	INV12067	IBIHM138-21	BOLD:AAX4976	OR796380
Apidae	*Nomadakohli* Schmiedeknecht, 1882	INV12090	IBIHM161-21	BOLD:AAX4976	OR796272
Apidae	*Nomadalinsenmaieri* Schwarz, 1974	INV12068	IBIHM139-21	BOLD:AAF3571	OR796021
Apidae	*Nomadalinsenmaieri* Schwarz, 1974	INV12069	IBIHM140-21	BOLD:AAF3571	OR796221
Apidae	*Nomadalinsenmaieri* Schwarz, 1974	INV13211	IBIHM782-21	BOLD:AAF3571	OR796156
Apidae	*Nomadalinsenmaieri* Schwarz, 1974	INV13267	IBIHM837-21	BOLD:AAF3571	OR796673
Apidae	*Nomadamaculicornis* Pérez, 1884	INV12070	IBIHM141-21	BOLD:AEO4739	OR796126
Apidae	*Nomadamarshamella* (Kirby, 1802)	INV09254	IBIHM067-20	BOLD:AAC4884	OR796390
Apidae	*Nomadamelathoracica* Imhoff, 1834	INV12071	IBIHM142-21	BOLD:AAX4983	OR796895
Apidae	*Nomadamelathoracica* Imhoff, 1834	INV12072	IBIHM143-21	BOLD:AAX4983	OR796185
Apidae	*Nomadamerceti* Alfken, 1909#	INV12073	IBIHM144-21	BOLD:AEO1963'	OR796423
Apidae	*Nomadamerceti* Alfken, 1909#	INV12074	IBIHM145-21	BOLD:AEO1963'	OR796917
Apidae	*Nomadamerceti* Alfken, 1909#	INV12084	IBIHM155-21	BOLD:AEO1963'	OR796138
Apidae	*Nomadamerceti* Alfken, 1909#	INV13210	IBIHM781-21	BOLD:AEO1963'	OR796735
Apidae	*Nomadamerceti* Alfken, 1909#	INV13212	IBIHM783-21	BOLD:AEO1963'	OR796586
Apidae	*Nomadamerceti* Alfken, 1909#	INV13213	IBIHM784-21	BOLD:AEO1963'	OR795932
Apidae	*Nomadaminuscula* Noskiewicz, 1930	INV12075	IBIHM146-21	BOLD:AAP1578	OR796161
Apidae	*Nomadamutabilis* Morawitz, 1870	INV12076	IBIHM147-21	BOLD:AAX4985	OR796609
Apidae	*Nomadanobilis* Herrich-Schäffer, 1839	INV12077	IBIHM148-21	BOLD:AAU8975	OR796447
Apidae	*Nomadanobilis* Herrich-Schäffer, 1839	INV13262	IBIHM832-21	BOLD:AAU8975	OR796171
Apidae	*Nomadapectoralis* Morawitz, 1877	INV12079	IBIHM150-21	BOLD:AEO0458	OR796426
Apidae	*Nomadapiccioliana* Magretti, 1883#	INV12080	IBIHM151-21	BOLD:AAF3496	OR796703
Apidae	*Nomadapiccioliana* Magretti, 1883#	INV12081	IBIHM152-21	BOLD:AAF3496	OR795947
Apidae	*Nomadarubricoxa* Schwarz, 1977#	INV12082	IBIHM153-21	BOLD:AEO1962'	OR796120
Apidae	*Nomadarubricoxa* Schwarz, 1977#	INV12083	IBIHM154-21	BOLD:AEO1962'	OR796308
Apidae	*Nomadarubricoxa* Schwarz, 1977#	INV12085	IBIHM156-21	BOLD:AEO1962'	OR796117
Apidae	*Nomadasanguinea* Smith, 1854	INV12086	IBIHM157-21	BOLD:AEN8255	OR796588
Apidae	*Nomadasanguinea* Smith, 1854	INV12087	IBIHM158-21	BOLD:AEN8255	OR796950
Apidae	*Nomadasanguinea* Smith, 1854	INV13271	IBIHM841-21	BOLD:AEN8255	OR796187
Apidae	*Nomadasexfasciata* Panzer, 1799	INV12088	IBIHM159-21	BOLD:AAI2916	OR796031
Apidae	*Nomadasexfasciata* Panzer, 1799	INV13269	IBIHM839-21	BOLD:AAI2916	OR795973
Apidae	*Nomadasheppardana* (Kirby, 1802)	INV12089	IBIHM160-21	BOLD:AAP1578	OR796595
Apidae	*Nomadasimilis* Morawitz, 1872	INV12091	IBIHM162-21	BOLD:AEX4551'	OR796147
Apidae	*Nomadastigma* Fabricius, 1804	INV12092	IBIHM163-21	BOLD:ABA8671	OR796450
Apidae	*Nomadastriata* Fabricius, 1793	INV12093	IBIHM164-21	BOLD:ABY7961	OR796506
Apidae	*Nomadastriata* Fabricius, 1793	INV12605	IBIHM1006-22	BOLD:ABY7961	OR796348
Apidae	*Nomadastriata* Fabricius, 1793	INV13206	IBIHM777-21	BOLD:ABY7961	OR795994
Apidae	*Nomadastriata* Fabricius, 1793	INV13208	IBIHM779-21	BOLD:ABY7961	OR796502
Apidae	*Nomadasuccincta* Panzer, 1798	INV12094	IBIHM165-21	BOLD:ABX5010	OR796256
Apidae	*Nomadasuccincta* Panzer, 1798	INV13261	IBIHM831-21	BOLD:ABX5010	OR796153
Apidae	*Pasitesmaculatus* Jurine, 1807	INV12027	IBIHM098-21	BOLD:AAL3976	OR796569
Apidae	*Tetraloniacinctella* (Saunders, 1908)	INV12102	IBIHM173-21	BOLD:AEN9450'	OR796857
Apidae	*Tetraloniadentata* (Germar, 1839)	INV12107	IBIHM178-21	BOLD:AEO2705'	OR796915
Apidae	*Tetraloniafulvescens* Giraud, 1863	INV12113	IBIHM184-21	BOLD:ADJ0585	OR796497
Apidae	*Tetraloniafulvescens* Giraud, 1863	INV12112	IBIHM183-21		OR796536
Apidae	*Tetraloniaiberica* Dusmet y Alonso 1926	INV12117	IBIHM188-21	BOLD:AER1385	OR796061
Apidae	*Tetralonianana* Morawitz, 1874	INV12119	IBIHM190-21	BOLD:ABW6400	OR796841
Apidae	*Tetraloniastrigata* (Lepeletier, 1841)	INV12125	IBIHM196-21	BOLD:AEN8168	OR796240
Apidae	*Thyreushirtus* (de Beaumont, 1940)	INV12004	IBIHM075-21	BOLD:AEO3985	OR796533
Apidae	*Thyreushirtus* (de Beaumont, 1940)	INV12005	IBIHM076-21	BOLD:AEO3985	OR796549
Apidae	*Thyreushirtus* (de Beaumont, 1940)	INV12007	IBIHM078-21	BOLD:AEO3985	OR796598
Apidae	*Thyreushistrionicus* (Illiger, 1806)	INV12457	IBIHM1310-22	BOLD:AAO3396	OR796245
Apidae	*Thyreushistrionicus* (Illiger, 1806)	INV12458	IBIHM1311-22	BOLD:AAO3396	OR796238
Apidae	*Thyreushistrionicus* (Illiger, 1806)	INV12460	IBIHM1313-22	BOLD:AAO3396	OR796771
Apidae	*Thyreusorbatus* (Lepeletier, 1841)	INV12006	IBIHM077-21	BOLD:AAI0456	OR796313
Apidae	*Thyreusorbatus* (Lepeletier, 1841)	INV12010	IBIHM081-21	BOLD:AAI0456	OR796110
Apidae	*Thyreusorbatus* (Lepeletier, 1841)	INV12011	IBIHM082-21	BOLD:AAI0456	OR796498
Apidae	*Thyreuspicaron* Lieftinck, 1968	INV12461	IBIHM1314-22	BOLD:AED2570	OR796358
Apidae	*Thyreusramosus* (Lepeletier, 1841)	INV12008	IBIHM079-21	BOLD:AAK1852	OR796224
Apidae	*Thyreusramosus* (Lepeletier, 1841)	INV12009	IBIHM080-21	BOLD:AAK1852	OR796037
Apidae	*Thyreustruncatus* (Pérez, 1883)	INV12459	IBIHM1312-22	BOLD:AES2873	OR795902
Apidae	*Xylocopacantabrita* Lepeletier, 1841#	INV12173	IBIHM244-21	BOLD:AEO2264	OR796867
Apidae	*Xylocopacantabrita* Lepeletier, 1841#	INV13040	IBIHM611-21	BOLD:AEO2264	OR796858
Apidae	*Xylocopairis* (Christ, 1791)	INV12172	IBIHM243-21	BOLD:AEO4657'	OR796217
Apidae	*Xylocopairis* (Christ, 1791)	INV13231	IBIHM801-21	BOLD:AEO4657'	OR796736
Apidae	*Xylocopavalga* Gerstäcker, 1872	INV12171	IBIHM242-21	BOLD:AEN0425	OR796293
Apidae	*Xylocopaviolacea* (Linnaeus, 1758)	INV12174	IBIHM245-21	BOLD:AAJ9209	OR796614
Apidae	*Xylocopaviolacea* (Linnaeus, 1758)	INV13232	IBIHM802-21	BOLD:AAJ9209	OR796465
Colletidae	*Colletesabeillei* Pérez, 1903	INV12347	IBIHM418-21	BOLD:ACP8014	OR796715
Colletidae	*Colletesabeillei* Pérez, 1903	INV13153	IBIHM724-21		OR796563
Colletidae	*Colletesacutus* Pérez, 1903	INV12348	IBIHM419-21	BOLD:AAI9265	OR796731
Colletidae	*Colletesalbomaculatus* (Lucas, 1849)	INV12349	IBIHM420-21	BOLD:ABU9046	OR796089
Colletidae	*Colletesalbomaculatus* (Lucas, 1849)	INV13308	IBIHM878-21	BOLD:ABU9046	OR795920
Colletidae	*Colletesdinizi* Kuhlmann, Ortiz & Ornosa, 2001#	INV12490	IBIHM1343-22	BOLD:AEU9447'	OR796281
Colletidae	*Colleteseous* Morice, 1904	INV12353	IBIHM424-21	BOLD:ADW0513	OR796015
Colletidae	*Colletesfodiens* (Fourcroy, 1785)	INV12354	IBIHM425-21	BOLD:ABZ6809	OR795931
Colletidae	*Colletesfoveolaris* Pérez, 1903	INV12484	IBIHM1337-22	BOLD:AEU9446	OR796674
Colletidae	*Colleteshylaeiformis* Eversmann, 1852	INV13309	IBIHM879-21	BOLD:AEU8542'	OR796326
Colletidae	*Colleteshylaeiformis* Eversmann, 1852	INV12355	IBIHM426-21	BOLD:AEW0951'	OR796829
Colletidae	*Colletesmarginatus* Smith, 1846	INV12358	IBIHM429-21	BOLD:AAN3911	OR796699
Colletidae	*Colletesmlokossewiczi* Radoszkowski, 1891	INV12487	IBIHM1340-22	BOLD:ABA8594	OR796561
Colletidae	*Colletesnigricans* Gistel, 1857	INV12357	IBIHM428-21	BOLD:AEZ9412	OR796581
Colletidae	*Colletesnigricans* Gistel, 1857	INV12485	IBIHM1338-22	BOLD:AEZ9412	OR796377
Colletidae	*Colletesnigricans* Gistel, 1857	INV13310	IBIHM880-21	BOLD:AEZ9412	OR796103
Colletidae	*Colletespulchellus* Pérez, 1903	INV12488	IBIHM1341-22	BOLD:AAN3911	OR796951
Colletidae	*Colletespulchellus* Pérez, 1903	INV12489	IBIHM1342-22	BOLD:AAN3911	OR796173
Colletidae	*Hylaeusangustatus* (Schenck, 1861)	INV12178	IBIHM249-21	BOLD:AAK3477	OR796750
Colletidae	*Hylaeusangustatus* (Schenck, 1861)	INV12194	IBIHM265-21	BOLD:AAK3477	OR796433
Colletidae	*Hylaeusangustatus* (Schenck, 1861)	INV13224	IBIHM795-21	BOLD:AEN9677'	OR796842
Colletidae	*Hylaeusangustatus* (Schenck, 1861)	INV13316	IBIHM886-21	BOLD:AEN9677'	OR796848
Colletidae	*Hylaeusannularis* (Kirby, 1802)	INV12179	IBIHM250-21	BOLD:AEK2943	OR796470
Colletidae	*Hylaeusannularis* (Kirby, 1802)	INV12180	IBIHM251-21	BOLD:AEK2943	OR796346
Colletidae	*Hylaeusannularis* (Kirby, 1802)	INV13320	IBIHM890-21	BOLD:AEK2943	OR796738
Colletidae	*Hylaeusclypearis* (Schenck, 1853)	INV12183	IBIHM254-21	BOLD:AAK3480	OR796853
Colletidae	*Hylaeuscommunis* Nylander, 1852	INV13222	IBIHM793-21	BOLD:AAE5080	OR796880
Colletidae	*Hylaeusconfusus* Nylander, 1852	INV12184	IBIHM255-21	BOLD:AAD9315	OR796100
Colletidae	*Hylaeuscoriaceus* (Pérez, 1895)	INV12195	IBIHM266-21	BOLD:AEO4115'	OR796508
Colletidae	*Hylaeuscoriaceus* (Pérez, 1895)	INV12196	IBIHM267-21	BOLD:AEO4115'	OR796623
Colletidae	*Hylaeuscornutus* Curtis, 1831	INV12185	IBIHM256-21	BOLD:AAF4031	OR796243
Colletidae	*Hylaeuscornutus* Curtis, 1831	INV13315	IBIHM885-21	BOLD:AAF4031	OR796231
Colletidae	*Hylaeuscrassanus* (Warncke, 1972)	INV12187	IBIHM258-21	BOLD:AEN8873'	OR796190
Colletidae	*Hylaeusdifformis* (Eversmann, 1852)	INV12186	IBIHM257-21	BOLD:AAK3468	OR796480
Colletidae	*Hylaeusdilatatus* (Kirby, 1802)	INV12498	IBIHM1351-22	BOLD:AEU1509'	OR796942
Colletidae	*Hylaeusdilatatus* (Kirby, 1802)	INV12188	IBIHM259-21		OR796510
Colletidae	*Hylaeusgredleri* Förster, 1871	INV12190	IBIHM261-21	BOLD:AEO3886'	OR796225
Colletidae	*Hylaeushyalinatus* Smith, 1842	INV12191	IBIHM262-21	BOLD:AAE1166	OR796593
Colletidae	*Hylaeushyalinatus* Smith, 1842	INV13156	IBIHM727-21	BOLD:AAE1166	OR796740
Colletidae	*Hylaeusimparilis* Förster, 1871	INV12192	IBIHM263-21	BOLD:AAE5079	OR796253
Colletidae	*Hylaeusincongruus* Förster, 1871	INV01584	IBIHM038-19	BOLD:ABU9205	OR796047
Colletidae	*Hylaeusincongruus* Förster, 1871	INV12189	IBIHM260-21	BOLD:ABU9205	OR795925
Colletidae	*Hylaeusincongruus* Förster, 1871	INV12200	IBIHM271-21	BOLD:ABU9205	OR796457
Colletidae	*Hylaeusincongruus* Förster, 1871	INV12201	IBIHM272-21	BOLD:ABU9205	OR796935
Colletidae	*Hylaeusincongruus* Förster, 1871	INV13154	IBIHM725-21	BOLD:ABU9205	OR796429
Colletidae	*Hylaeusincongruus* Förster, 1871	INV13155	IBIHM726-21	BOLD:ABU9205	OR796655
Colletidae	*Hylaeusincongruus* Förster, 1871	INV13157	IBIHM728-21	BOLD:ABU9205	OR795953
Colletidae	*Hylaeusincongruus* Förster, 1871	INV13223	IBIHM794-21	BOLD:ABU9205	OR796339
Colletidae	*Hylaeuskahri* Förster, 1871	INV12193	IBIHM264-21	BOLD:AAN3379	OR796084
Colletidae	*Hylaeuslineolatus* (Schenck, 1861)	INV12496	IBIHM1349-22	BOLD:AAI8781	OR796924
Colletidae	*Hylaeuslineolatus* (Schenck, 1861)	INV12497	IBIHM1350-22	BOLD:AAI8781	OR796005
Colletidae	*Hylaeuspenalaris* Dathe, 1979#	INV12197	IBIHM268-21	BOLD:AEO2266'	OR796414
Colletidae	*Hylaeuspictipes* Nylander, 1852	INV12198	IBIHM269-21	BOLD:AAK3488	OR796642
Colletidae	*Hylaeuspictipes* Nylander, 1852	INV13314	IBIHM884-21	BOLD:AAK3488	OR796490
Colletidae	*Hylaeuspictipes* Nylander, 1852	INV13319	IBIHM889-21	BOLD:AAK3488	OR796261
Colletidae	*Hylaeuspictus* (Smith, 1853)	INV12202	IBIHM273-21	BOLD:AEO2267	OR796295
Colletidae	*Hylaeuspilosulus* (Pérez, 1903)	INV12203	IBIHM274-21	BOLD:AAX2603	OR796362
Colletidae	*Hylaeuspilosulus* (Pérez, 1903)	INV12204	IBIHM275-21	BOLD:AAX2603	OR796658
Colletidae	*Hylaeuspunctatus* (Brullé, 1832)	INV12205	IBIHM276-21	BOLD:AAE5060	OR796056
Colletidae	*Hylaeuspunctulatissimus* Smith, 1842	INV12206	IBIHM277-21	BOLD:AAK3323	OR796210
Colletidae	*Hylaeussignatus* (Panzer, 1798)	INV12207	IBIHM278-21	BOLD:AAI8577	OR796312
Colletidae	*Hylaeussoror* (Pérez, 1903)	INV12499	IBIHM1352-22	BOLD:AET8250'	OR796434
Colletidae	*Hylaeussulphuripes* (Gribodo, 1894)	INV12208	IBIHM279-21	BOLD:AEJ3211	OR796043
Colletidae	*Hylaeustaeniolatus* Förster, 1871	INV12199	IBIHM270-21	BOLD:AEO5452'	OR796300
Colletidae	*Hylaeusvariegatus* (Fabricius, 1798)	INV12209	IBIHM280-21	BOLD:AAK3338	OR796896
Colletidae	*Hylaeusvariegatus* (Fabricius, 1798)	INV12210	IBIHM281-21	BOLD:AAK3338	OR796050
Colletidae	*Hylaeusvariegatus* (Fabricius, 1798)	INV13317	IBIHM887-21	BOLD:AAK3338	OR795903
Halictidae	*Ceylalictusvariegatus* (Olivier, 1789)	INV12279	IBIHM350-21	BOLD:AAV7281	OR796006
Halictidae	*Ceylalictusvariegatus* (Olivier, 1789)	INV12281	IBIHM352-21	BOLD:AAV7281	OR796925
Halictidae	*Dufoureagaullei* Vachal, 1897#	INV12275	IBIHM346-21	BOLD:AEN9216'	OR796099
Halictidae	*Dufoureagaullei* Vachal, 1897#	INV12276	IBIHM347-21	BOLD:AEN9216'	OR796808
Halictidae	*Dufoureagaullei* Vachal, 1897#	INV13293	IBIHM863-21	BOLD:AEN9216'	OR795909
Halictidae	*Dufoureahalictula* (Nylander, 1852)	INV13217	IBIHM788-21	BOLD:AAI9074	OR796349
Halictidae	*Dufoureatrautmanni* Dusmet y Alonso, 1935#	INV12273	IBIHM344-21	BOLD:AEN3645	OR796622
Halictidae	*Dufoureatrautmanni* Dusmet y Alonso, 1935#	INV12274	IBIHM345-21	BOLD:AEN3645	OR796922
Halictidae	*Halictusbrunnescens* (Eversmann, 1852)	INV12259	IBIHM330-21	BOLD:AAE3033	OR796366
Halictidae	*Halictusbrunnescens* (Eversmann, 1852)	INV12263	IBIHM334-21	BOLD:AAE3033	OR796704
Halictidae	*Halictusbrunnescens* (Eversmann, 1852)	INV13054	IBIHM625-21	BOLD:AAE3033	OR796014
Halictidae	*Halictusconfusus* Smith, 1853	INV12257	IBIHM328-21	BOLD:AEN9591'	OR795965
Halictidae	*Halictuscrenicornis* Blüthgen, 1923	INV12258	IBIHM329-21	BOLD:AAD5869	OR796521
Halictidae	*Halictuscrenicornis* Blüthgen, 1923	INV13055	IBIHM626-21	BOLD:AAD5869	OR796393
Halictidae	*Halictusfulvipes* (Klug, 1817)	INV02289	IBIHM013-19	BOLD:ADS5152	OR796212
Halictidae	*Halictusgemmeus* Dours, 1872	INV01552	IBIHM032-19	BOLD:ABV4897	OR796916
Halictidae	*Halictusgemmeus* Dours, 1872	INV01553	IBIHM005-19	BOLD:ABV4897	OR796430
Halictidae	*Halictusgemmeus* Dours, 1872	INV12260	IBIHM331-21	BOLD:ABV4897	OR796392
Halictidae	*Halictusgemmeus* Dours, 1872	INV13058	IBIHM629-21	BOLD:ABV4897	OR796602
Halictidae	*Halictusmaculatus* Smith, 1848	INV13053	IBIHM624-21	BOLD:ACH4344	OR796541
Halictidae	*Halictusmaculatus* Smith, 1848	INV12261	IBIHM332-21	BOLD:AEO0160'	OR796283
Halictidae	*Halictuspollinosus* Sichel, 1860	INV12262	IBIHM333-21	BOLD:ADZ7031	OR796345
Halictidae	*Halictuspollinosus* Sichel, 1860	INV13218	IBIHM789-21	BOLD:ADZ7031	OR796620
Halictidae	*Halictuspollinosus* Sichel, 1860	INV13276	IBIHM846-21	BOLD:ADZ7031	OR796705
Halictidae	*Halictusquadripartitus* Blüthgen, 1923	INV13274	IBIHM844-21	BOLD:AEW0985	OR796296
Halictidae	*Halictusrubicundus* (Christ, 1791)	INV12265	IBIHM336-21	BOLD:AAA3534	OR796486
Halictidae	*Halictusscabiosae* (Rossi, 1790)	INV13047	IBIHM618-21	BOLD:AAN4492	OR796578
Halictidae	*Halictusscabiosae* (Rossi, 1790)	INV13048	IBIHM619-21	BOLD:AAN4492	OR796127
Halictidae	*Halictusseladonius* (Fabricius, 1794)#	INV12267	IBIHM338-21	BOLD:AEL5026	OR796301
Halictidae	*Halictusseladonius* (Fabricius, 1794)#	INV13062	IBIHM633-21	BOLD:AEL5026	OR795962
Halictidae	*Halictusseladonius* (Fabricius, 1794)#	INV15673	IBIHY016-22	BOLD:AEL5026	OR796677
Halictidae	*Halictussexcinctus* (Fabricius, 1775)	INV12264	IBIHM335-21	BOLD:AAI6486	OR795933
Halictidae	*Halictussmaragdulus* Vachal, 1895	INV01583	IBIHM006-19	BOLD:ABV8170	OR796761
Halictidae	*Halictussmaragdulus* Vachal, 1895	INV02260	IBIHM041-19	BOLD:ABV8170	OR796804
Halictidae	*Halictussmaragdulus* Vachal, 1895	INV02317	IBIHM044-19	BOLD:ABV8170	OR796213
Halictidae	*Halictussmaragdulus* Vachal, 1895	INV12270	IBIHM341-21	BOLD:ABV8170	OR796407
Halictidae	*Halictussubauratus* (Rossi, 1792)	INV01563	IBIHM034-19	BOLD:AAI6480	OR796314
Halictidae	*Halictussubauratus* (Rossi, 1792)	INV01579	IBIHM036-19	BOLD:AAI6480	OR796948
Halictidae	*Halictussubauratus* (Rossi, 1792)	INV02297	IBIHM014-19	BOLD:AAI6480	OR796785
Halictidae	*Halictussubauratus* (Rossi, 1792)	INV02318	IBIHM045-19	BOLD:AAI6480	OR796388
Halictidae	*Halictussubauratus* (Rossi, 1792)	INV12268	IBIHM339-21	BOLD:AAI6480	OR796189
Halictidae	*Halictussubauratus* (Rossi, 1792)	INV13059	IBIHM630-21	BOLD:AAI6480	OR796619
Halictidae	*Halictussubauratus* (Rossi, 1792)	INV13060	IBIHM631-21	BOLD:AAI6480	OR796511
Halictidae	*Halictussubauratus* (Rossi, 1792)	INV13061	IBIHM632-21	BOLD:AAI6480	OR796052
Halictidae	*Halictussubauratus* (Rossi, 1792)	INV13277	IBIHM847-21	BOLD:AAI6480	OR796020
Halictidae	*Halictussubmediterraneus* (Pauly, 2015)	INV12269	IBIHM340-21	BOLD:AAK4967	OR796807
Halictidae	*Halictussubmediterraneus* (Pauly, 2015)	INV13050	IBIHM621-21	BOLD:AAK4967	OR796277
Halictidae	*Halictusvestitus* Lepeletier, 1841	INV12271	IBIHM342-21	BOLD:ACU5548	OR796104
Halictidae	*Lasioglossumaeratum* (Kirby, 1802)	INV12310	IBIHM381-21	BOLD:AAJ3200	OR796901
Halictidae	*Lasioglossumalbocinctum* (Lucas, 1849)	INV12299	IBIHM370-21	BOLD:AAE5835	OR795996
Halictidae	*Lasioglossumalbocinctum* (Lucas, 1849)	INV13072	IBIHM643-21	BOLD:AAE5835	OR795978
Halictidae	*Lasioglossumalbocinctum* (Lucas, 1849)	INV13074	IBIHM645-21	BOLD:AAE5835	OR796790
Halictidae	*Lasioglossumalgericolellum* (Strand, 1909)	INV12492	IBIHM1345-22	BOLD:AEN2990	OR796290
Halictidae	*Lasioglossumalgericolellum* (Strand, 1909)	INV12493	IBIHM1346-22	BOLD:AEN2990	OR795985
Halictidae	*Lasioglossumalgericolellum* (Strand, 1909)	INV13102	IBIHM673-21	BOLD:AEN2990	OR796756
Halictidae	*Lasioglossumangusticeps* (Perkins, 1895)	INV12494	IBIHM1347-22	BOLD:AAK8433	OR796241
Halictidae	*Lasioglossumangusticeps* (Perkins, 1895)	INV13114	IBIHM685-21	BOLD:AAK8433	OR796610
Halictidae	*Lasioglossumaureolum* (Pérez, 1903)	INV12613	IBIHM1014-22	BOLD:ADZ5328	OR796354
Halictidae	*Lasioglossumaureolum* (Pérez, 1903)	INV13107	IBIHM678-21	BOLD:ADZ5328	OR796462
Halictidae	*Lasioglossumaureolum* (Pérez, 1903)	INV13108	IBIHM679-21	BOLD:ADZ5328	OR796111
Halictidae	*Lasioglossumaureolum* (Pérez, 1903)	INV13109	IBIHM680-21	BOLD:ADZ5328	OR796332
Halictidae	*Lasioglossumbimaculatum* (Dours, 1872)	INV12301	IBIHM372-21	BOLD:AAW9939	OR796222
Halictidae	*Lasioglossumbimaculatum* (Dours, 1872)	INV13286	IBIHM856-21	BOLD:AAW9939	OR796873
Halictidae	*Lasioglossumbimaculatum* (Dours, 1872)	INV13287	IBIHM857-21	BOLD:AAW9939	OR796402
Halictidae	*Lasioglossumbrevicorne* (Schenck, 1870)	INV12302	IBIHM373-21	BOLD:AAK8357	OR796406
Halictidae	*Lasioglossumbrevicorne* (Schenck, 1870)	INV13104	IBIHM675-21	BOLD:AAK8357	OR796403
Halictidae	*Lasioglossumbrevicorne* (Schenck, 1870)	INV13105	IBIHM676-21	BOLD:AAK8357	OR796165
Halictidae	*Lasioglossumbreviventre* (Schenck, 1853)	INV12303	IBIHM374-21	BOLD:AAY8321	OR796298
Halictidae	*Lasioglossumbuccale* (Pérez, 1903)	INV12304	IBIHM375-21	BOLD:ADZ4420	OR796734
Halictidae	*Lasioglossumbuccale* (Pérez, 1903)	INV13281	IBIHM851-21	BOLD:ADZ4420	OR796229
Halictidae	*Lasioglossumcalceatum* (Scopoli, 1763)	INV12318	IBIHM389-21	BOLD:AAB0353	OR796321
Halictidae	*Lasioglossumcalceatum* (Scopoli, 1763)	INV13086	IBIHM657-21	BOLD:AAB0353	OR796631
Halictidae	*Lasioglossumcalceatum* (Scopoli, 1763)	INV13089	IBIHM660-21	BOLD:AAB0353	OR796926
Halictidae	*Lasioglossumcalceatum* (Scopoli, 1763)	INV12305	IBIHM376-21	BOLD:AEO1453'	OR796851
Halictidae	*Lasioglossumcallizonium* (Pérez, 1896)	INV12306	IBIHM377-21	BOLD:ABA0006	OR796460
Halictidae	*Lasioglossumcallizonium* (Pérez, 1896)	INV13066	IBIHM637-21	BOLD:ABA0006	OR796952
Halictidae	*Lasioglossumcorvinum* (Morawitz, 1876)	INV12312	IBIHM383-21	BOLD:ACR3210	OR796045
Halictidae	*Lasioglossumcostulatum* (Kriechbaumer, 1873)	INV12313	IBIHM384-21	BOLD:AAJ3116	OR796353
Halictidae	*Lasioglossumcostulatum* (Kriechbaumer, 1873)	INV13075	IBIHM646-21	BOLD:AAJ3116	OR796176
Halictidae	*Lasioglossumdiscus* (Smith, 1853)#	INV02261	IBIHM007-19	BOLD:ABA0007	OR796702
Halictidae	*Lasioglossumdiscus* (Smith, 1853)#	INV02333	IBIHM017-19	BOLD:ABA0007	OR796055
Halictidae	*Lasioglossumdiscus* (Smith, 1853)#	INV12314	IBIHM385-21	BOLD:ABA0007	OR796303
Halictidae	*Lasioglossumdiscus* (Smith, 1853)#	INV13291	IBIHM861-21	BOLD:ABA0007	OR796335
Halictidae	*Lasioglossumdusmeti* (Blüthgen, 1924)#	INV12315	IBIHM386-21	BOLD:AEN9127'	OR796098
Halictidae	*Lasioglossumdusmeti* (Blüthgen, 1924)#	INV13288	IBIHM858-21	BOLD:AEN9127'	OR796653
Halictidae	*Lasioglossumglabriusculum* (Morawitz, 1872)	INV12316	IBIHM387-21	BOLD:AAZ1489	OR796016
Halictidae	*Lasioglossumgriseolum* (Morawitz, 1872)	INV12495	IBIHM1348-22	BOLD:AET1905'	OR796488
Halictidae	*Lasioglossumibericum* Ebmer, 1975#	INV12317	IBIHM388-21	BOLD:AAJ1741	OR796707
Halictidae	*Lasioglossumimmunitum* (Vachal, 1895)#	INV13083	IBIHM654-21	BOLD:AEW5079'	OR796193
Halictidae	*Lasioglossuminterruptum* (Panzer, 1798)	INV13093	IBIHM664-21	BOLD:ABU8540	OR795984
Halictidae	*Lasioglossuminterruptum* (Panzer, 1798)	INV13290	IBIHM860-21	BOLD:ABU8540	OR795938
Halictidae	*Lasioglossuminterruptum* (Panzer, 1798)	INV12319	IBIHM390-21	BOLD:AFK8181	OR796652
Halictidae	*Lasioglossumlaevigatum* (Kirby, 1802)	INV12320	IBIHM391-21	BOLD:AAK8392	OR796656
Halictidae	*Lasioglossumlativentre* (Schenck, 1853)	INV12321	IBIHM392-21	BOLD:AAE1129	OR796681
Halictidae	*Lasioglossumleucopus* (Kirby, 1802)	INV12614	IBIHM1015-22	BOLD:AAF3848	OR795974
Halictidae	*Lasioglossumleucozonium* (Schrank, `78`)	INV13113	IBIHM684-21	BOLD:AEN9620	OR796178
Halictidae	*Lasioglossumleucozonium* (Schrank, 1781)	INV13112	IBIHM683-21	BOLD:AAA2322	OR796526
Halictidae	*Lasioglossumleucozonium* (Schrank, 1781)	INV12322	IBIHM393-21	BOLD:AEN9620	OR795969
Halictidae	*Lasioglossumleucozonium* (Schrank, 1781)	INV13064	IBIHM635-21	BOLD:AEN9620	OR796566
Halictidae	*Lasioglossumleucozonium* (Schrank, 1781)	INV13110	IBIHM681-21	BOLD:AEN9620	OR796764
Halictidae	*Lasioglossumleucozonium* (Schrank, 1781)	INV13111	IBIHM682-21	BOLD:AEN9620	OR796471
Halictidae	*Lasioglossumlimbellum* (Morawitz, 1876)	INV12323	IBIHM394-21	BOLD:ADZ7745	OR796454
Halictidae	*Lasioglossumlittorale* (Blüthgen, 1924)	INV12615	IBIHM1016-22	BOLD:ADZ2937	OR795906
Halictidae	*Lasioglossumlittorale* (Blüthgen, 1924)	INV12616	IBIHM1017-22	BOLD:ADZ2937	OR796708
Halictidae	*Lasioglossumlucidulum* (Schenck, 1861)	INV12338	IBIHM409-21	BOLD:AAE5918	OR796530
Halictidae	*Lasioglossumlucidulum* (Schenck, 1861)	INV12617	IBIHM1018-22	BOLD:AAE5918	OR796667
Halictidae	*Lasioglossummalachurum* (Kirby, 1802)	INV12324	IBIHM395-21	BOLD:AAE5496	OR795908
Halictidae	*Lasioglossummalachurum* (Kirby, 1802)	INV13092	IBIHM663-21	BOLD:AEO8786'	OR796130
Halictidae	*Lasioglossummandibulare* (Morawitz, 1866)	INV12326	IBIHM397-21	BOLD:AEO4941	OR796211
Halictidae	*Lasioglossummarginatum* (Brullé, 1832)	INV12325	IBIHM396-21	BOLD:AFA0740	OR796273
Halictidae	*Lasioglossummaurusium* (Blüthgen, 1935)	INV12797	IBIHM1198-22	BOLD:ADZ2523	OR795966
Halictidae	*Lasioglossummediterraneum* (Blüthgen, 1926)#	INV12327	IBIHM398-21	BOLD:AEO8781	OR796365
Halictidae	*Lasioglossummediterraneum* (Blüthgen, 1926)#	INV13292	IBIHM862-21	BOLD:AEO8781	OR796599
Halictidae	*Lasioglossumminutissimum* (Kirby, 1802)	INV12328	IBIHM399-21	BOLD:ACQ8646	OR796936
Halictidae	*Lasioglossumminutissimum* (Kirby, 1802)	INV13094	IBIHM665-21	BOLD:ACQ8646	OR796887
Halictidae	*Lasioglossumminutissimum* (Kirby, 1802)	INV13095	IBIHM666-21	BOLD:ACQ8646	OR796063
Halictidae	*Lasioglossummorio* (Fabricius, 1793)	INV12618	IBIHM1019-22	BOLD:AAD9335	OR796267
Halictidae	*Lasioglossummorio* (Fabricius, 1793)	INV12619	IBIHM1020-22	BOLD:AAD9335	OR796671
Halictidae	*Lasioglossummorio* (Fabricius, 1793)	INV13229	IBIHM800-21	BOLD:AAD9335	OR796651
Halictidae	*Lasioglossummorio* (Fabricius, 1793)	INV15678	IBIHY021-22	BOLD:AAD9335	OR796442
Halictidae	*Lasioglossumnitidiusculum* (Kirby, 1802)	INV12329	IBIHM400-21	BOLD:AAZ9229	OR796415
Halictidae	*Lasioglossumorihuelicum* (Blüthgen, 1924)#	INV12330	IBIHM401-21	BOLD:AEO0527'	OR796381
Halictidae	*Lasioglossumpallens* (Brullé, 1832)	INV12331	IBIHM402-21	BOLD:ADM1219	OR796492
Halictidae	*Lasioglossumparvulum* (Schenck, 1853)	INV12346	IBIHM417-21	BOLD:ADT1404	OR796636
Halictidae	*Lasioglossumpauperatum* (Brullé, 1832)	INV01536	IBIHM004-19	BOLD:AAK7827	OR796092
Halictidae	*Lasioglossumpauperatum* (Brullé, 1832)	INV02264	IBIHM008-19	BOLD:AAK7827	OR796162
Halictidae	*Lasioglossumpauperatum* (Brullé, 1832)	INV02277	IBIHM009-19	BOLD:AAK7827	OR796363
Halictidae	*Lasioglossumpauperatum* (Brullé, 1832)	INV12332	IBIHM403-21	BOLD:AAK7827	OR796713
Halictidae	*Lasioglossumpauperatum* (Brullé, 1832)	INV13067	IBIHM638-21	BOLD:AAK7827	OR796665
Halictidae	*Lasioglossumpauperatum* (Brullé, 1832)	INV13097	IBIHM668-21	BOLD:AAK7827	OR795905
Halictidae	*Lasioglossumpauperatum* (Brullé, 1832)	INV13098	IBIHM669-21	BOLD:AAK7827	OR796933
Halictidae	*Lasioglossumpauperatum* (Brullé, 1832)	INV13103	IBIHM674-21	BOLD:AAK7827	OR796579
Halictidae	*Lasioglossumpauperatum* (Brullé, 1832)	INV13284	IBIHM854-21	BOLD:AAK7827	OR795904
Halictidae	*Lasioglossumpauxillum* (Schenck, 1853)	INV12334	IBIHM405-21		OR796938
Halictidae	*Lasioglossumperclavipes* (Blüthgen, 1934)#	INV12333	IBIHM404-21	BOLD:AEO0577'	OR796046
Halictidae	*Lasioglossumprasinum* (Smith, 1848)#	INV12335	IBIHM406-21	BOLD:AEO4817'	OR796466
Halictidae	*Lasioglossumpunctatissimum* (Schenck, 1853)	INV01547	IBIHM030-19	BOLD:AAF4021	OR796625
Halictidae	*Lasioglossumpunctatissimum* (Schenck, 1853)	INV12336	IBIHM407-21	BOLD:AAF4021	OR796928
Halictidae	*Lasioglossumpunctatissimum* (Schenck, 1853)	INV13106	IBIHM677-21	BOLD:AAF4021	OR796663
Halictidae	*Lasioglossumpunctatissimum* (Schenck, 1853)	INV13279	IBIHM849-21	BOLD:AAF4021	OR796643
Halictidae	*Lasioglossumpuncticolle* (Morawitz, 1872)	INV12337	IBIHM408-21	BOLD:AEO0839'	OR796458
Halictidae	*Lasioglossumsexnotatum* (Kirby, 1802)	INV12339	IBIHM410-21	BOLD:AAW9816	OR796662
Halictidae	*Lasioglossumsexnotatum* (Kirby, 1802)	INV13070	IBIHM641-21	BOLD:AAW9816	OR796422
Halictidae	*Lasioglossumsexnotatum* (Kirby, 1802)	INV13078	IBIHM649-21	BOLD:AAW9816	OR796555
Halictidae	*Lasioglossumsphecodimorphum* (Vachal, 1892)	INV12341	IBIHM412-21	BOLD:AEO3826	OR796739
Halictidae	*Lasioglossumsphecodimorphum* (Vachal, 1892)#	INV13285	IBIHM855-21	BOLD:AEO3826	OR795942
Halictidae	*Lasioglossumstrictifrons* (Vachal, 1895)	INV12622	IBIHM1023-22	BOLD:AEN9032	OR796669
Halictidae	*Lasioglossumstrictifrons* (Vachal, 1895)	INV13068	IBIHM639-21	BOLD:AEN9032	OR796523
Halictidae	*Lasioglossumstrictifrons* (Vachal, 1895)	INV13079	IBIHM650-21	BOLD:AEN9032	OR796628
Halictidae	*Lasioglossumstrictifrons* (Vachal, 1895)	INV13099	IBIHM670-21	BOLD:AEN9032	OR796597
Halictidae	*Lasioglossumstrictifrons* (Vachal, 1895)	INV13100	IBIHM671-21	BOLD:AEN9032	OR796515
Halictidae	*Lasioglossumstrictifrons* (Vachal, 1895)	INV13101	IBIHM672-21	BOLD:AEN9032	OR796716
Halictidae	*Lasioglossumsubhirtum* (Lepeletier, 1841)	INV12340	IBIHM411-21	BOLD:ADM2541	OR796753
Halictidae	*Lasioglossumsubhirtum* (Lepeletier, 1841)	INV13090	IBIHM661-21	BOLD:ADM2541	OR796338
Halictidae	*Lasioglossumtransitorium* (Schenck, 1868)	INV13084	IBIHM655-21	BOLD:AEM8844	OR796539
Halictidae	*Lasioglossumtransitoriumplanulum* (Pérez, 1903)	INV12623	IBIHM1024-22	BOLD:AEM8844	OR796531
Halictidae	*Lasioglossumtransitoriumplanulum* (Pérez, 1903)	INV12625	IBIHM1026-22	BOLD:AEM8844	OR796478
Halictidae	*Lasioglossumtransitoriumplanulum* (Pérez, 1903)	INV12624	IBIHM1025-22	BOLD:AER6557	OR796352
Halictidae	*Lasioglossumvillosulum* (Kirby, 1802)	INV12342	IBIHM413-21	BOLD:AEW2300	OR796793
Halictidae	*Lasioglossumvillosulum* (Kirby, 1802)	INV13069	IBIHM640-21	BOLD:AEW2300	OR796878
Halictidae	*Lasioglossumvillosulum* (Kirby, 1802)	INV13088	IBIHM659-21	BOLD:AEW2300	OR796416
Halictidae	*Lasioglossumvillosulum* (Kirby, 1802)	INV13280	IBIHM850-21	BOLD:AEW2300	OR796228
Halictidae	*Lasioglossumvillosulum* (Kirby, 1802)	INV13283	IBIHM853-21	BOLD:AEW2300	OR796884
Halictidae	*Lasioglossumvillosulum* (Kirby, 1802)	INV15687	IBIHY028-22	BOLD:AEW2300	OR796233
Halictidae	*Lasioglossumvirens* (Erichson, 1835)#	INV12839	IBIHM1240-22	BOLD:AAZ9232	OR796242
Halictidae	*Lasioglossumvirens* (Erichson, 1835)#	INV12840	IBIHM1241-22	BOLD:AAZ9232	OR796152
Halictidae	*Lasioglossumxanthopus* (Kirby, 1802)	INV12344	IBIHM415-21	BOLD:AAE1789	OR796032
Halictidae	*Lasioglossumxanthopus* (Kirby, 1802)	INV13278	IBIHM848-21	BOLD:AEW1421	OR795914
Halictidae	*Lasioglossumzonulum* (Smith, 1848)	INV12345	IBIHM416-21	BOLD:AAB3147	OR796817
Halictidae	*Lasioglossumzonulum* (Smith, 1848)	INV13077	IBIHM648-21	BOLD:AAB3147	OR796309
Halictidae	*Lasioglossumzonulum* (Smith, 1848)	INV13080	IBIHM651-21	BOLD:AAB3147	OR796763
Halictidae	*Lasioglossumzonulum* (Smith, 1848)	INV13081	IBIHM652-21	BOLD:AAB3147	OR796514
Halictidae	*Lasioglossumzonulum* (Smith, 1848)	INV13082	IBIHM653-21	BOLD:AAB3147	OR796513
Halictidae	*Nomiapisdiversipes* (Latreille, 1806)	INV02319	IBIHM016-19	BOLD:AAF6016	OR796483
Halictidae	*Nomiapisdiversipes* (Latreille, 1806)	INV12278	IBIHM349-21	BOLD:AAF6016	OR796846
Halictidae	*Nomiapisdiversipes* (Latreille, 1806)	INV13219	IBIHM790-21	BOLD:AAF6016	OR796329
Halictidae	*Nomiapisdiversipes* (Latreille, 1806)	INV13273	IBIHM843-21	BOLD:AAF6016	OR796328
Halictidae	*Nomiapispaulyi* Wood & Le Divelec, 2022	INV13272	IBIHM842-21	BOLD:ADD4589	OR796401
Halictidae	*Nomioidesfacilis* (Smith, 1853)	INV12280	IBIHM351-21	BOLD:AEJ1330	OR796135
Halictidae	*Nomioidesminutissimus* (Rossi, 1790)	INV12282	IBIHM353-21	BOLD:AEO2657'	OR796520
Halictidae	*Sphecodesalbilabris* (Fabricius, 1793)	INV12283	IBIHM354-21	BOLD:AAI0130	OR796694
Halictidae	*Sphecodesalternatus* Smith, 1853	INV12284	IBIHM355-21	BOLD:ACD7802	OR795918
Halictidae	*Sphecodesalternatus* Smith, 1853	INV13303	IBIHM873-21	BOLD:AEO6580	OR795940
Halictidae	*Sphecodescrassanus* Warncke, 1992	INV12285	IBIHM356-21	BOLD:ACD7802	OR796474
Halictidae	*Sphecodescrassanus* Warncke, 1992	INV13294	IBIHM864-21	BOLD:ACD7802	OR796115
Halictidae	*Sphecodescroaticus* Meyer, 1922	INV12286	IBIHM357-21	BOLD:ACP5320	OR796439
Halictidae	*Sphecodesephippius* (Linnaeus, 1767)	INV12287	IBIHM358-21	BOLD:AAD0332	OR795936
Halictidae	*Sphecodesephippius* (Linnaeus, 1767)	INV13298	IBIHM868-21	BOLD:AAD0332	OR796660
Halictidae	*Sphecodesgibbus* (Linnaeus, 1758)	INV12289	IBIHM360-21	BOLD:AAD5232	OR796455
Halictidae	*Sphecodeshirtellus* Blüthgen, 1923#	INV13117	IBIHM688-21	BOLD:AEO0791'	OR796859
Halictidae	*Sphecodeslongulus* Hagens, 1882	INV13116	IBIHM687-21	BOLD:AFM8564	OR796939
Halictidae	*Sphecodesmarginatus* Hagens, 1882	INV12308	IBIHM379-21	BOLD:AAY9961	OR796732
Halictidae	*Sphecodesmarginatus* Hagens, 1882	INV13085	IBIHM656-21	BOLD:AAY9961	OR796758
Halictidae	*Sphecodesmarginatus* Hagens, 1882	INV13295	IBIHM865-21	BOLD:AAY9961	OR796911
Halictidae	*Sphecodesmonilicornis* (Kirby, 1802)	INV12290	IBIHM361-21	BOLD:AAI0259	OR796749
Halictidae	*Sphecodesmonilicornis* (Kirby, 1802)	INV13121	IBIHM692-21	BOLD:AAI0259	OR796438
Halictidae	*Sphecodesmonilicornis* (Kirby, 1802)	INV13297	IBIHM867-21	BOLD:AAI0259	OR796947
Halictidae	*Sphecodesmonilicornis* (Kirby, 1802)	INV13304	IBIHM874-21	BOLD:AAI0259	OR796125
Halictidae	*Sphecodesmonilicornis* (Kirby, 1802)	INV13305	IBIHM875-21	BOLD:AAI0259	OR796204
Halictidae	*Sphecodesniger* Hagens, 1874	INV13115	IBIHM686-21	BOLD:AAN0084	OR796179
Halictidae	*Sphecodesolivieri* Lepeletier, 1825	INV12291	IBIHM362-21	BOLD:ACR8452	OR796589
Halictidae	*Sphecodespellucidus* Smith, 1845	INV12848	IBIHM1249-22	BOLD:AAI0260	OR796409
Halictidae	*Sphecodespseudofasciatus* Blüthgen, 1925	INV13289	IBIHM859-21	BOLD:AEO2633'	OR796725
Halictidae	*Sphecodespuncticeps* Thomson, 1870	INV12288	IBIHM359-21	BOLD:AAY9709	OR796664
Halictidae	*Sphecodespuncticeps* Thomson, 1870	INV12293	IBIHM364-21	BOLD:AAY9709	OR796418
Halictidae	*Sphecodespuncticeps* Thomson, 1870	INV13299	IBIHM869-21	BOLD:AAY9709	OR796341
Halictidae	*Sphecodespuncticeps* Thomson, 1870	INV13300	IBIHM870-21	BOLD:AAY9709	OR796556
Halictidae	*Sphecodespuncticeps* Thomson, 1870	INV13306	IBIHM876-21	BOLD:AAY9709	OR796011
Halictidae	*Sphecodesreticulatus* Thomson, 1870	INV12309	IBIHM380-21	BOLD:ABV3743	OR796220
Halictidae	*Sphecodesreticulatus* Thomson, 1870	INV13296	IBIHM866-21	BOLD:ABV3743	OR796903
Halictidae	*Sphecodesrubicundus* Hagens, 1875	INV13119	IBIHM690-21	BOLD:AAJ3388	OR796026
Halictidae	*Sphecodesrubicundus* Hagens, 1875	INV13122	IBIHM693-21	BOLD:AAJ3388	OR796827
Halictidae	*Sphecodesrubicundus* Hagens, 1875	INV13302	IBIHM872-21	BOLD:AAJ3388	OR796605
Halictidae	*Sphecodesrubripes* Spinola, 1838#	INV12294	IBIHM365-21	BOLD:AAM4113	OR796687
Halictidae	*Sphecodesruficrus* (Erichson, 1835)	INV12296	IBIHM367-21	BOLD:AAF2925	OR795901
Halictidae	*Sphecodesruficrus* (Erichson, 1835)	INV13120	IBIHM691-21	BOLD:AAF2925	OR796013
Halictidae	*Sphecodesruficrus* (Erichson, 1835)	INV13123	IBIHM694-21	BOLD:AAF2925	OR796553
Halictidae	*Sphecodesruficrus* (Erichson, 1835)	INV13124	IBIHM695-21	BOLD:AAF2925	OR796373
Halictidae	*Sphecodesruficrus* (Erichson, 1835)	INV13301	IBIHM871-21	BOLD:AAF2925	OR796906
Halictidae	*Sphecodesrufiventris* (Panzer, 1798)	INV12295	IBIHM366-21	BOLD:ABA8649	OR796177
Halictidae	*Sphecodesspinulosus* Hagens, 1875	INV12297	IBIHM368-21	BOLD:ACQ2281	OR796564
Halictidae	*Systrophagrandimargo* Pérez, 1905	INV12272	IBIHM343-21	BOLD:AEO6068'	OR796633
Megachilidae	*Afranthidiumcarduelemalacopygum* (Gribodo, 1894)	INV12447	IBIHM1300-22	BOLD:AEO1769	OR796464
Megachilidae	*Afranthidiumcarduelemalacopygum* (Gribodo, 1894)	INV12448	IBIHM1301-22	BOLD:AEO1769	OR796832
Megachilidae	*Afranthidiumcarduelemalacopygum* (Gribodo, 1894)	INV13349	IBIHM919-21	BOLD:AEO1769	OR796188
Megachilidae	*Afranthidiumschulthessii* (Friese, 1897)	INV12211	IBIHM282-21	BOLD:AEO4048	OR796024
Megachilidae	*Aglaoapistridentata* (Nylander, 1848)	INV12022	IBIHM093-21	BOLD:AAO2962	OR796801
Megachilidae	*Anthidiellumbreviusculum* (Pérez, 1890)	INV12212	IBIHM283-21	BOLD:AEN7284	OR796051
Megachilidae	*Anthidiellumstrigatum* (Panzer, 1804)	INV02280	IBIHM010-19	BOLD:AAY8402	OR796788
Megachilidae	*Anthidiellumstrigatum* (Panzer, 1804)	INV12213	IBIHM284-21	BOLD:AAY8402	OR796748
Megachilidae	*Anthidiellumstrigatum* (Panzer, 1804)	INV13350	IBIHM920-21	BOLD:AAY8402	OR796325
Megachilidae	*Anthidiumcingulatum* Latreille, 1809	INV12215	IBIHM286-21	BOLD:AAN9423	OR796467
Megachilidae	*Anthidiumdiadema* Latreille, 1809	INV12445	IBIHM1298-22	BOLD:AER0584	OR796499
Megachilidae	*Anthidiumdiadema* Latreille, 1809	INV12446	IBIHM1299-22	BOLD:AET3713	OR796149
Megachilidae	*Anthidiumflorentinum* (Fabricius, 1775)	INV12214	IBIHM285-21	BOLD:ABW0328	OR796484
Megachilidae	*Anthidiumflorentinum* (Fabricius, 1775)	INV15674	IBIHY017-22	BOLD:ABW0328	OR795941
Megachilidae	*Anthidiumloti* Perris, 1852	INV12216	IBIHM287-21	BOLD:ACG1108	OR796007
Megachilidae	*Anthidiummanicatum* (Linnaeus, 1758)	INV12217	IBIHM288-21	BOLD:AAA9946	OR796247
Megachilidae	*Anthidiummanicatum* (Linnaeus, 1758)	INV13142	IBIHM713-21	BOLD:AAA9946	OR796417
Megachilidae	*Anthidiummanicatum* (Linnaeus, 1758)	INV13143	IBIHM714-21	BOLD:AAA9946	OR796645
Megachilidae	*Anthidiumoblongatum* (Illiger, 1806)	INV12219	IBIHM290-21	BOLD:AAC5344	OR795959
Megachilidae	*Anthidiumpunctatum* Latreille, 1809	INV12218	IBIHM289-21	BOLD:AEN2704	OR796680
Megachilidae	*Anthidiumtaeniatum* Latreille, 1809	INV12220	IBIHM291-21	BOLD:AEO3965	OR796160
Megachilidae	*Anthidiumtaeniatum* Latreille, 1809	INV13341	IBIHM911-21	BOLD:AEO3965	OR796399
Megachilidae	*Chelostomacampanularum* (Kirby, 1802)	INV13220	IBIHM791-21	BOLD:AAB1123	OR796035
Megachilidae	*Chelostomacampanularum* (Kirby, 1802)	INV13221	IBIHM792-21	BOLD:AAB1123	OR796600
Megachilidae	*Chelostomacampanularum* (Kirby, 1802)	INV12443	IBIHM1296-22	BOLD:AET7155'	OR796495
Megachilidae	*Chelostomacampanularum* (Kirby, 1802)	INV12444	IBIHM1297-22	BOLD:AET7155'	OR796053
Megachilidae	*Chelostomaflorisomne* (Linnaeus, 1758)	INV12442	IBIHM1295-22	BOLD:AEU4316'	OR796494
Megachilidae	*Chelostomarapunculi* (Lepeletier, 1841)	INV12400	IBIHM1253-22	BOLD:AAA7122	OR796768
Megachilidae	*Chelostomarapunculi* (Lepeletier, 1841)	INV12401	IBIHM1254-22	BOLD:AAA7122	OR796371
Megachilidae	*Coelioxysacanthurus* (Illiger, 1806)	INV12449	IBIHM1302-22	BOLD:AET0371	OR796065
Megachilidae	*Coelioxysafer* Lepeletier, 1841	INV12450	IBIHM1303-22	BOLD:AAV8048	OR796344
Megachilidae	*Coelioxysafer* Lepeletier, 1841	INV13347	IBIHM917-21	BOLD:AAV8048	OR796385
Megachilidae	*Coelioxysargenteus* Lepeletier, 1841	INV01263	IBIHM023-19	BOLD:AAV9350	OR796944
Megachilidae	*Coelioxysargenteus* Lepeletier, 1841	INV12031	IBIHM102-21	BOLD:AAV9350	OR796524
Megachilidae	*Coelioxysaurolimbatus* Förster, 1853	INV12452	IBIHM1305-22	BOLD:AES4301'	OR796710
Megachilidae	*Coelioxysbrevis* Eversmann, 1852	INV12032	IBIHM103-21	BOLD:ADD7530	OR796712
Megachilidae	*Coelioxysechinatus* Förster, 1853	INV12034	IBIHM105-21	BOLD:AET4410'	OR796408
Megachilidae	*Coelioxysechinatus* Förster, 1853	INV12451	IBIHM1304-22	BOLD:AET4410'	OR796886
Megachilidae	*Coelioxyshaemorrhoa* Förster, 1853	INV12033	IBIHM104-21	BOLD:ADC9939	OR796175
Megachilidae	*Coelioxyshaemorrhoa* Förster, 1853	INV12453	IBIHM1306-22	BOLD:ADC9939	OR796411
Megachilidae	*Coelioxyshaemorrhoa* Förster, 1853	INV12454	IBIHM1307-22	BOLD:ADC9939	OR796918
Megachilidae	*Coelioxysobtusus* Pérez, 1884	INV12455	IBIHM1308-22	BOLD:AEO4483'	OR795979
Megachilidae	*Coelioxysobtusus* Pérez, 1884	INV13345	IBIHM915-21	BOLD:AEO4483'	OR796752
Megachilidae	*Coelioxysobtusus* Pérez, 1884	INV13346	IBIHM916-21	BOLD:AEO4483'	OR796683
Megachilidae	*Dioxyscinctus* (Jurine, 1807)	INV12017	IBIHM088-21	BOLD:AAY8179	OR795977
Megachilidae	*Dioxyscinctus* (Jurine, 1807)	INV12018	IBIHM089-21	BOLD:AAY8179	OR796094
Megachilidae	*Dioxyspumilus* Gerstäcker, 1869	INV12019	IBIHM090-21	BOLD:AEO4333'	OR796459
Megachilidae	*Dioxyspumilus* Gerstäcker, 1869	INV12020	IBIHM091-21	BOLD:AEO4333'	OR796017
Megachilidae	*Dioxyspumilus* Gerstäcker, 1869	INV12021	IBIHM092-21	BOLD:AEO4333'	OR796503
Megachilidae	*Enslinianabidentata* (Friese, 1899)#	INV12016	IBIHM087-21	BOLD:AEN9289'	OR796930
Megachilidae	*Heriadescrenulata* Nylander, 1856	INV12579	IBIHM550-21	BOLD:AAO0527	OR796208
Megachilidae	*Heriadescrenulata* Nylander, 1856	INV13355	IBIHM925-21	BOLD:AAO0527	OR795921
Megachilidae	*Heriadesrubicola* Pérez, 1890	INV12578	IBIHM549-21	BOLD:ABV4003	OR796690
Megachilidae	*Heriadesrubicola* Pérez, 1890	INV13354	IBIHM924-21	BOLD:ABV4003	OR796343
Megachilidae	*Heriadestruncorum* (Linnaeus, 1758)	INV12596	IBIHM567-21	BOLD:AAI8422	OR796706
Megachilidae	*Heriadestruncorum* (Linnaeus, 1758)	INV13125	IBIHM696-21	BOLD:AAI8422	OR796003
Megachilidae	*Heriadestruncorum* (Linnaeus, 1758)	INV13356	IBIHM926-21	BOLD:AAI8422	OR796811
Megachilidae	*Hoplitisacuticornis* (Dufour & Perris, 1840)	INV12386	IBIHM457-21	BOLD:AAK6030	OR796711
Megachilidae	*Hoplitisadunca* (Panzer, 1798)	INV12421	IBIHM1274-22	BOLD:AAI1794	OR796834
Megachilidae	*Hoplitisalbiscopa* (Friese, 1899)#	INV12411	IBIHM1264-22	BOLD:AEO0400'	OR796864
Megachilidae	*Hoplitisalbiscopa* (Friese, 1899)#	INV12412	IBIHM1265-22	BOLD:AEO0400'	OR796350
Megachilidae	*Hoplitisalbiscopa* (Friese, 1899)#	INV13368	IBIHM938-21	BOLD:AEO0400'	OR796606
Megachilidae	*Hoplitisannulata* (Latreille, 1811)	INV12383	IBIHM454-21	BOLD:ADD6906	OR796334
Megachilidae	*Hoplitisannulata* (Latreille, 1811)	INV13366	IBIHM936-21	BOLD:ADD6906	OR796278
Megachilidae	*Hoplitisanthocopoides* (Schenck, 1853)	INV12422	IBIHM1275-22	BOLD:AAC3950	OR796540
Megachilidae	*Hoplitisantigae* (Pérez, 1895)	INV12416	IBIHM1269-22	BOLD:AEN2131	OR796920
Megachilidae	*Hoplitisantigae* (Pérez, 1895)	INV12599	IBIHM570-21	BOLD:AEN2131	OR796927
Megachilidae	*Hoplitisantigae* (Pérez, 1895)	INV13369	IBIHM939-21	BOLD:AEN2131	OR796276
Megachilidae	*Hoplitisbenoisti* (Alfken, 1935)	INV12423	IBIHM1276-22	BOLD:AEV6823'	OR796146
Megachilidae	*Hoplitisbisulca* (Gerstäcker, 1869)	INV12588	IBIHM559-21	BOLD:AEO0529	OR796955
Megachilidae	*Hoplitisbrachypogon* (Pérez, 1879)#	INV12393	IBIHM464-21	BOLD:AEP1421	OR796219
Megachilidae	*Hoplitisbrachypogon* (Pérez, 1879)#	INV13367	IBIHM937-21	BOLD:AEP1421	OR795944
Megachilidae	*Hoplitiscadiza* (Warncke, 1991)#	INV12419	IBIHM1272-22	BOLD:AER8784'	OR796351
Megachilidae	*Hoplitiscadiza* (Warncke, 1991)#	INV12420	IBIHM1273-22	BOLD:AER8784'	OR796941
Megachilidae	*Hoplitiscampanularis* (Morawitz, 1879)#	INV12413	IBIHM1266-22	BOLD:AES6061'	OR796849
Megachilidae	*Hoplitiscampanularis* (Morawitz, 1879)#	INV12414	IBIHM1267-22	BOLD:AES6061'	OR796517
Megachilidae	*Hoplitisclaviventris* (Thomson, 1872)	INV12402	IBIHM1255-22	BOLD:AAE5480	OR796781
Megachilidae	*Hoplitiscristatula* (van der Zanden, 1990)	INV12593	IBIHM564-21	BOLD:AEL6177	OR796087
Megachilidae	*Hoplitisgrumi* (Morawitz, 1894)#	INV12403	IBIHM1256-22	BOLD:AET3653'	OR796482
Megachilidae	*Hoplitisleucomelana* (Kirby, 1802)	INV12398	IBIHM469-21	BOLD:AEL2158	OR796262
Megachilidae	*Hoplitismarchali* (Pérez, 1902)	INV12404	IBIHM1257-22	BOLD:AET3654'	OR796375
Megachilidae	*Hoplitisochraceicornis* (Ferton, 1902)#	INV12424	IBIHM1277-22	BOLD:AER7475'	OR796745
Megachilidae	*Hoplitisochraceicornis* (Ferton, 1902)#	INV12425	IBIHM1278-22	BOLD:AER7475'	OR796551
Megachilidae	*Hoplitispraestans* (Morawitz, 1894)	INV12415	IBIHM1268-22	BOLD:AER7476'	OR796883
Megachilidae	*Hoplitisravouxi* (Pérez, 1902)	INV12417	IBIHM1270-22	BOLD:AAI1840	OR796297
Megachilidae	*Hoplitisravouxi* (Pérez, 1902)	INV12418	IBIHM1271-22	BOLD:AET2330'	OR796284
Megachilidae	*Hoplitisstecki* (Frey-Gessner, 1908)	INV02288	IBIHM012-19	BOLD:ACF9884	OR796747
Megachilidae	*Hoplitisstecki* (Frey-Gessner, 1908)	INV12577	IBIHM548-21	BOLD:ACF9884	OR796934
Megachilidae	*Hoplitistridentata* (Dufour & Perris, 1840)	INV12592	IBIHM563-21	BOLD:AAF2246	OR796545
Megachilidae	*Icteranthidiumgrohmanni* (Spinola, 1838)	INV12221	IBIHM292-21	BOLD:AEH0395	OR796554
Megachilidae	*Icteranthidiumgrohmanni* (Spinola, 1838)	INV13353	IBIHM923-21	BOLD:AEH0395	OR796612
Megachilidae	*Lithurguschrysurus* Fonscolombe, 1834	INV12234	IBIHM305-21	BOLD:AAY7690	OR796128
Megachilidae	*Lithurgustibialis* Morawitz, 1875	INV12235	IBIHM306-21	BOLD:ABW1174	OR796131
Megachilidae	*Megachilealbisecta* (Klug, 1817)	INV12237	IBIHM308-21	BOLD:AEO5749	OR796529
Megachilidae	*Megachilealbisecta* (Klug, 1817)	INV02293	IBIHM043-19	BOLD:AEO5750	OR796820
Megachilidae	*Megachilealbonotata* Radoszkowski, 1886#	INV12236	IBIHM307-21	BOLD:AAY7863	OR796644
Megachilidae	*Megachileapicalis* Spinola, 1808	INV12238	IBIHM309-21	BOLD:AAF4159	OR796843
Megachilidae	*Megachileapicalis* Spinola, 1808	INV13127	IBIHM698-21	BOLD:AAF4159	OR796073
Megachilidae	*Megachileapicalis* Spinola, 1808	INV13342	IBIHM912-21	BOLD:AAF4159	OR796453
Megachilidae	*Megachileargentata* (Fabricius, 1793)	INV03735	IBIHM048-19	BOLD:AAD7803	OR796463
Megachilidae	*Megachileargentata* (Fabricius, 1793)	INV12437	IBIHM1290-22	BOLD:AAD7803	OR796168
Megachilidae	*Megachileargentata* (Fabricius, 1793)	INV12438	IBIHM1291-22	BOLD:AAD7803	OR796062
Megachilidae	*Megachileargentata* (Fabricius, 1793)	INV13129	IBIHM700-21	BOLD:AAD7803	OR796759
Megachilidae	*Megachileargentata* (Fabricius, 1793)	INV13343	IBIHM913-21	BOLD:AAD7803	OR796717
Megachilidae	*Megachileargentata* (Fabricius, 1793)	INV13348	IBIHM918-21	BOLD:AAD7803	OR795928
Megachilidae	*Megachileargentata* (Fabricius, 1793)	INV15662	IBIHY006-22	BOLD:AAD7803	OR796548
Megachilidae	*Megachileargentata* (Fabricius, 1793)	INV15675	IBIHY018-22	BOLD:AAD7803	OR796040
Megachilidae	*Megachilecentuncularis* (Linnaeus, 1758)	INV12239	IBIHM310-21	BOLD:AAA8356	OR796591
Megachilidae	*Megachilecentuncularis* (Linnaeus, 1758)	INV13130	IBIHM701-21	BOLD:AAA8356	OR796214
Megachilidae	*Megachilecentuncularis* (Linnaeus, 1758)	INV15682	IBIHY024-22	BOLD:AAA8356	OR795976
Megachilidae	*Megachilecircumcincta* (Kirby, 1802)	INV12240	IBIHM311-21	BOLD:AAC0049	OR796424
Megachilidae	*Megachileericetorum* Lepeletier, 1841	INV12241	IBIHM312-21	BOLD:AEA9090	OR796315
Megachilidae	*Megachilegiraudi* Gerstäcker, 1869	INV12242	IBIHM313-21	BOLD:AAY7848	OR796814
Megachilidae	*Megachilegiraudi* Gerstäcker, 1869	INV12250	IBIHM321-21	BOLD:AAY7848	OR795983
Megachilidae	*Megachilelagopoda* (Linnaeus, 1761)	INV12243	IBIHM314-21	BOLD:AAE8874	OR796800
Megachilidae	*Megachileleachella* Curtis, 1828	INV12244	IBIHM315-21	BOLD:AAD2767	OR796799
Megachilidae	*Megachileleachella* Curtis, 1828	INV13126	IBIHM697-21	BOLD:AAD2767	OR796560
Megachilidae	*Megachilelefebvrei* (Lepeletier, 1841)	INV12245	IBIHM316-21	BOLD:ABW1753	OR796585
Megachilidae	*Megachilemarginata* Smith, 1853	INV12256	IBIHM327-21	BOLD:AAK6960	OR796666
Megachilidae	*Megachilemaritima* (Kirby, 1802)	INV12246	IBIHM317-21	BOLD:AAD7888	OR796336
Megachilidae	*Megachilemelanopyga* Costa, 1863	INV12247	IBIHM318-21	BOLD:AAE8870	OR796688
Megachilidae	*Megachilemelanopyga* Costa, 1863	INV13128	IBIHM699-21	BOLD:AAE8870	OR796692
Megachilidae	*Megachileoctosignata* Nylander, 1852	INV12248	IBIHM319-21	BOLD:ADM5545	OR796519
Megachilidae	*Megachileopacifrons* Pérez, 1897	INV12249	IBIHM320-21	BOLD:ACF9800	OR796342
Megachilidae	*Megachilepusilla* Pérez, 1884	INV12254	IBIHM325-21	BOLD:AAQ0516	OR796440
Megachilidae	*Megachilepusilla* Pérez, 1884	INV15709	IBIHY032-22	BOLD:AAQ0516	OR796806
Megachilidae	*Megachilepyrenaica* Lepeletier, 1841	INV12251	IBIHM322-21	BOLD:AAO0637	OR795950
Megachilidae	*Megachilerotundata* (Fabricius, 1787)	INV12252	IBIHM323-21	BOLD:AEO2625'	OR796888
Megachilidae	*Megachilesicula* (Rossi, 1792)	INV12253	IBIHM324-21	BOLD:AEO4733	OR796310
Megachilidae	*Megachilethevestensis* Ferton, 1909#	INV12439	IBIHM1292-22	BOLD:AEU0213'	OR796095
Megachilidae	*Megachileversicolor* Smith, 1844	INV12440	IBIHM1293-22	BOLD:AAD5414	OR796286
Megachilidae	*Megachileversicolor* Smith, 1844	INV12441	IBIHM1294-22	BOLD:AAD5414	OR796813
Megachilidae	*Megachilewillughbiella* (Kirby, 1802)	INV12255	IBIHM326-21	BOLD:ACE6545	OR796784
Megachilidae	*Osmiaanceyi* Pérez, 1879#	INV12435	IBIHM1288-22	BOLD:AEO4345'	OR795934
Megachilidae	*Osmiaanceyi* Pérez, 1879#	INV12436	IBIHM1289-22	BOLD:AEO4345'	OR796078
Megachilidae	*Osmiaanceyi* Pérez, 1879#	INV13361	IBIHM931-21	BOLD:AEO4345'	OR796194
Megachilidae	*Osmiaandrenoides* Spinola, 1808	INV12585	IBIHM556-21	BOLD:AEO0032'	OR796613
Megachilidae	*Osmiaargyropyga* Pérez, 1879#	INV12582	IBIHM553-21	BOLD:AEO4346'	OR796318
Megachilidae	*Osmiaaurulenta* (Panzer, 1799)	INV12580	IBIHM551-21	BOLD:AAE5409	OR796218
Megachilidae	*Osmiaaurulenta* (Panzer, 1799)	INV13131	IBIHM702-21	BOLD:AAE5409	OR796819
Megachilidae	*Osmiabicornis* (Linnaeus, 1758)	INV12434	IBIHM1287-22	BOLD:AAD6282	OR796570
Megachilidae	*Osmiabicornis* (Linnaeus, 1758)	INV12590	IBIHM561-21	BOLD:AAD6282	OR796571
Megachilidae	*Osmiabicornis* (Linnaeus, 1758)	INV13140	IBIHM711-21	BOLD:AAD6282	OR796206
Megachilidae	*Osmiabicornis* (Linnaeus, 1758)	INV13141	IBIHM712-21	BOLD:AAD6282	OR796069
Megachilidae	*Osmiabrevicornis* (Fabricius, 1798)	INV12586	IBIHM557-21	BOLD:AEF6206	OR796722
Megachilidae	*Osmiacaerulescens* (Linnaeus, 1758)	INV01272	IBIHM024-19	BOLD:AAD0313	OR796932
Megachilidae	*Osmiacaerulescens* (Linnaeus, 1758)	INV12389	IBIHM460-21	BOLD:AAD0313	OR796122
Megachilidae	*Osmiacaerulescens* (Linnaeus, 1758)	INV13132	IBIHM703-21	BOLD:AAD0313	OR796264
Megachilidae	*Osmiacaerulescens* (Linnaeus, 1758)	INV13133	IBIHM704-21	BOLD:AAD0313	OR796121
Megachilidae	*Osmiacaerulescens* (Linnaeus, 1758)	INV13136	IBIHM707-21	BOLD:AAD0313	OR796831
Megachilidae	*Osmiacaerulescens* (Linnaeus, 1758)	INV13362	IBIHM932-21	BOLD:AAD0313	OR796700
Megachilidae	*Osmiacaerulescens* (Linnaeus, 1758)	INV13364	IBIHM934-21	BOLD:AAD0313	OR796822
Megachilidae	*Osmiacephalotes* Morawitz, 1870	INV12584	IBIHM555-21	BOLD:AEN9982'	OR796902
Megachilidae	*Osmiacyanoxantha* Pérez, 1879#	INV12387	IBIHM458-21	BOLD:AEO1610'	OR796769
Megachilidae	*Osmiadimidiata* Morawitz, 1870	INV12583	IBIHM554-21	BOLD:AEO3684	OR796840
Megachilidae	*Osmiaemarginata* Lepeletier, 1841	INV12589	IBIHM560-21	BOLD:AAE4126	OR796550
Megachilidae	*Osmiaferruginea* Latreille, 1811	INV12390	IBIHM461-21	BOLD:AEN8200'	OR796956
Megachilidae	*Osmiagallarum* Spinola, 1808	INV12388	IBIHM459-21	BOLD:AAO8736	OR796919
Megachilidae	*Osmiaiberica* van der Zanden, 1987#	INV12581	IBIHM552-21	BOLD:AEN8862'	OR796166
Megachilidae	*Osmialabialis* Pérez, 1879	INV12591	IBIHM562-21	BOLD:ADY8979	OR796634
Megachilidae	*Osmialabialis* Pérez, 1879	INV13360	IBIHM930-21	BOLD:ADY8979	OR796812
Megachilidae	*Osmialatreillei* (Spinola, 1806)	INV12397	IBIHM468-21	BOLD:AAZ7870	OR796226
Megachilidae	*Osmialeaiana* (Kirby, 1802)	INV12433	IBIHM1286-22	BOLD:AAI1846	OR795900
Megachilidae	*Osmialigurica* Morawitz, 1868	INV12391	IBIHM462-21	BOLD:ADH8891	OR796157
Megachilidae	*Osmialigurica* Morawitz, 1868	INV13134	IBIHM705-21	BOLD:ADH8891	OR796542
Megachilidae	*Osmialunata* Benoist, 1928#	INV12594	IBIHM565-21	BOLD:AEO3079'	OR796181
Megachilidae	*Osmiamelanogaster* Spinola, 1808	INV12392	IBIHM463-21	BOLD:AEN4264	OR796787
Megachilidae	*Osmianiveata* (Fabricius, 1804)	INV12399	IBIHM470-21	BOLD:AAP2416	OR796451
Megachilidae	*Osmianiveata* (Fabricius, 1804)	INV13137	IBIHM708-21	BOLD:AAP2416	OR796054
Megachilidae	*Osmianiveata* (Fabricius, 1804)	INV13138	IBIHM709-21	BOLD:AAP2416	OR796289
Megachilidae	*Osmianiveata* (Fabricius, 1804)	INV13363	IBIHM933-21	BOLD:AAP2416	OR796743
Megachilidae	*Osmianiveocincta* Pérez, 1879#	INV12394	IBIHM465-21	BOLD:AEO5031'	OR796088
Megachilidae	*Osmianiveocincta* Pérez, 1879#	INV12432	IBIHM1285-22	BOLD:AEO5031'	OR796215
Megachilidae	*Osmiarufohirta* Latreille, 1811	INV12396	IBIHM467-21	BOLD:AEO0004'	OR796661
Megachilidae	*Osmiascutellaris* Morawitz, 1868	INV12428	IBIHM1281-22	BOLD:AEJ4270	OR795981
Megachilidae	*Osmiascutellaris* Morawitz, 1868	INV12429	IBIHM1282-22	BOLD:AEJ4270	OR796066
Megachilidae	*Osmiasignata* Erichson, 1839	INV12395	IBIHM466-21	BOLD:AEA2838	OR796369
Megachilidae	*Osmiasignata* Erichson, 1839	INV13139	IBIHM710-21	BOLD:AEA2838	OR796012
Megachilidae	*Osmiasubmicans* Morawitz, 1870	INV12385	IBIHM456-21	BOLD:AAK5820	OR796167
Megachilidae	*Osmiasubmicans* Morawitz, 1870	INV13135	IBIHM706-21	BOLD:AAK5820	OR796821
Megachilidae	*Osmiatergestensis* Ducke, 1897	INV12426	IBIHM1279-22	BOLD:AES2567	OR796151
Megachilidae	*Osmiatergestensis* Ducke, 1897	INV12427	IBIHM1280-22	BOLD:AES2567	OR796693
Megachilidae	*Osmiatricornis* Latreille, 1811	INV12587	IBIHM558-21	BOLD:AEN7876	OR796603
Megachilidae	*Osmiauncicornis* Pérez, 1895#	INV12430	IBIHM1283-22	BOLD:AET6037'	OR796119
Megachilidae	*Osmiauncicornis* Pérez, 1895#	INV12431	IBIHM1284-22	BOLD:AET6037'	OR796632
Megachilidae	*Osmiaversicolor* Latreille, 1811	INV12384	IBIHM455-21	BOLD:AAZ7638	OR796491
Megachilidae	*Protosmiaasensioi* Griswold & Parker, 1987#	INV12409	IBIHM1262-22	BOLD:AET0069'	OR796232
Megachilidae	*Protosmiacapitata* (Schletterer, 1889)#	INV12595	IBIHM566-21	BOLD:AEO6065'	OR796234
Megachilidae	*Protosmiaexenterata* (Pérez, 1895)#	INV12407	IBIHM1260-22	BOLD:ACL7801	OR796481
Megachilidae	*Protosmiaexenterata* (Pérez, 1895)#	INV12408	IBIHM1261-22	BOLD:ACL7801	OR796034
Megachilidae	*Protosmiaglutinosa* (Giraud, 1871)#	INV12405	IBIHM1258-22	BOLD:AET0068'	OR796626
Megachilidae	*Protosmiaglutinosa* (Giraud, 1871)#	INV12406	IBIHM1259-22	BOLD:AET0068'	OR796923
Megachilidae	*Pseudoanthidiumeximium* (Giraud, 1863)	INV12222	IBIHM293-21	BOLD:AEO0467	OR796544
Megachilidae	*Pseudoanthidiummelanurum* (Klug, 1832)	INV12223	IBIHM294-21	BOLD:AEN8472'	OR796397
Megachilidae	*Pseudoanthidiumreticulatum* (Mocsáry, 1884)	INV12226	IBIHM297-21	BOLD:AEO1983	OR796518
Megachilidae	*Pseudoanthidiumscapulare* (Latreille, 1809)	INV12224	IBIHM295-21	BOLD:AAK2762	OR796572
Megachilidae	*Pseudoanthidiumscapulare* (Latreille, 1809)	INV13351	IBIHM921-21	BOLD:AAK2762	OR796139
Megachilidae	*Pseudoanthidiumscapulare* (Latreille, 1809)	INV13352	IBIHM922-21	BOLD:AAK2762	OR796446
Megachilidae	*Pseudoanthidiumstigmaticorne* (Dours, 1873)	INV12225	IBIHM296-21	BOLD:ABV1020	OR795943
Megachilidae	*Rhodanthidiuminfuscatum* (Erichson, 1839)	INV12233	IBIHM304-21	BOLD:AEO5272	OR796410
Megachilidae	*Rhodanthidiumseptemdentatum* (Latreille, 1809)	INV12232	IBIHM303-21	BOLD:AAY5674	OR796686
Megachilidae	*Rhodanthidiumsiculum* (Spinola, 1838)	INV12230	IBIHM301-21	BOLD:AEA0375	OR796863
Megachilidae	*Rhodanthidiumsticticum* (Fabricius, 1787)	INV12231	IBIHM302-21	BOLD:AEH2459	OR796679
Megachilidae	*Stelisbreviuscula* (Nylander, 1848)	INV12026	IBIHM097-21	BOLD:ACE4655	OR796532
Megachilidae	*Stelisbreviuscula* (Nylander, 1848)	INV13357	IBIHM927-21	BOLD:ACE4655	OR796105
Megachilidae	*Stelisnasuta* (Latreille, 1809)	INV12000	IBIHM071-21	BOLD:AEC4732	OR796907
Megachilidae	*Stelisortizi* M.Schwarz & Gusenleitner, 2010#	INV12023	IBIHM094-21	BOLD:AEO2242'	OR796249
Megachilidae	*Stelisphaeoptera* (Kirby, 1802)	INV12024	IBIHM095-21	BOLD:AAY9796	OR796535
Megachilidae	*Stelispunctulatissima* (Kirby, 1802)	INV12025	IBIHM096-21	BOLD:AAJ4972	OR795972
Megachilidae	*Trachusabyssina* (Panzer, 1798)	INV12228	IBIHM299-21	BOLD:AAF1587	OR796792
Megachilidae	*Trachusainterrupta* (Fabricius, 1781)	INV12229	IBIHM300-21	BOLD:ACW1366	OR796154
Megachilidae	*Trachusalaeviventris* (Dours, 1873)	INV12227	IBIHM298-21	BOLD:AEN7816	OR796437
Melittidae	*Dasypodacingulata* Erichson, 1835	INV12480	IBIHM1333-22	BOLD:ACK7071	OR796071
Melittidae	*Dasypodacingulata* Erichson, 1835	INV12481	IBIHM1334-22	BOLD:ACK7071	OR796577
Melittidae	*Dasypodacingulata* Erichson, 1835	INV13148	IBIHM719-21	BOLD:ACK7071	OR796207
Melittidae	*Dasypodacrassicornis* Friese, 1896	INV12366	IBIHM437-21	BOLD:AEO3711	OR795961
Melittidae	*Dasypodacrassicornis* Friese, 1896	INV13311	IBIHM881-21	BOLD:AEO3711	OR796874
Melittidae	*Dasypodacrassicornis* Friese, 1896	INV13313	IBIHM883-21	BOLD:AEO3711	OR796331
Melittidae	*Dasypodadusmeti* Quilis, 1928	INV12363	IBIHM434-21	BOLD:ACK5966	OR796505
Melittidae	*Dasypodadusmeti* Quilis, 1928	INV13144	IBIHM715-21	BOLD:ACK5966	OR796584
Melittidae	*Dasypodadusmeti* Quilis, 1928	INV13152	IBIHM723-21	BOLD:ACK5966	OR796682
Melittidae	*Dasypodahirtipes* (Fabricius, 1793)	INV12365	IBIHM436-21	BOLD:AEO2254'	OR796810
Melittidae	*Dasypodahirtipes* (Fabricius, 1793)	INV13145	IBIHM716-21	BOLD:AEO2254'	OR796001
Melittidae	*Dasypodahirtipes* (Fabricius, 1793)	INV13151	IBIHM722-21	BOLD:AEO2254'	OR796845
Melittidae	*Dasypodamorotei* Quilis, 1928	INV12362	IBIHM433-21	BOLD:AEN9179	OR796897
Melittidae	*Dasypodamorotei* Quilis, 1928	INV12478	IBIHM1331-22	BOLD:AEN9179	OR796496
Melittidae	*Dasypodamorotei* Quilis, 1928	INV12479	IBIHM1332-22	BOLD:AEN9179	OR795926
Melittidae	*Dasypodamorotei* Quilis, 1928	INV13312	IBIHM882-21	BOLD:AEN9179	OR796109
Melittidae	*Dasypodapyrotrichia* Förster, 1855	INV12476	IBIHM1329-22	BOLD:AES3843'	OR796042
Melittidae	*Dasypodaradchenkoi* Ghisbain & Wood, 2023	INV12361	IBIHM432-21	BOLD:AEO4511'	OR796794
Melittidae	*Dasypodaradchenkoi* Ghisbain & Wood, 2023	INV12477	IBIHM1330-22	BOLD:AEO4511'	OR796019
Melittidae	*Dasypodavisnaga* (Rossi, 1790)	INV12367	IBIHM438-21	BOLD:AEO0075	OR796650
Melittidae	*Macropisfulvipes* (Fabricius, 1804)	INV12360	IBIHM431-21	BOLD:AAJ1462	OR795995
Melittidae	*Macropisfulvipes* (Fabricius, 1804)	INV13321	IBIHM891-21	BOLD:AAJ1462	OR796427
Melittidae	*Melittaleporina* (Panzer, 1799)	INV12368	IBIHM439-21	BOLD:AAF4411	OR796838

**Table 2.** Bee species from Iberia that were assigned to two or more BINs. Percentage of divergence represents the maximum sequence distance between specimens from our dataset in the BINs nominally comprising a species.

No.	Species	No. of BINs	% Divergence	Sympatric	Geographic origin	BIN
1	*Amegillaalbigena* (Lepeletier, 1841)	2	14.02	No	Spain (Murcia)	BOLD:AEL4482
					Portugal (Castelo Branco), Spain (Granada)	BOLD:AEO2968
2	*Andrenaampla* Warncke, 1967	2	2.81	No	Portugal (Aveiro)	BOLD:ABA2611
					Spain (Ávila)	BOLD:AES0278
3	*Andrenahesperia* Smith, 1853	2	10.20	Yes	Portugal (Beja)	BOLD:AEN4997
					Portugal (Beja; Castelo Branco)	BOLD:AEO5653
4	*Andrenalimata* Smith, 1853	2	3.77	Yes	Portugal (Castelo Branco), Spain (Girona)	BOLD:AAE1815
					Portugal (Castelo Branco)	BOLD:AEX3903
5	*Andrenamorio* Brullé, 1832	2	2.64	Yes	Portugal (Faro, Castelo Branco)	BOLD:AAJ2141
					Portugal (Castelo Branco)	BOLD:AER2061
6	*Andrenapropinqua* Schenck, 1853	2	5.41	Yes	Portugal (Castelo Branco, Coimbra), Spain (Málaga)	BOLD:AAJ2115
					Portugal (Aveiro, Castelo Branco, Coimbra)	BOLD:AEN9473
7	*Andrenarussula* Lepeletier, 1841	2	5.60	No	Portugal (Castelo Branco, Santarém)	BOLD:AAZ1205
					Portugal (Faro, Setubal)	BOLD:AEN8931
8	*Anthidiumdiadema* Latreille, 1809	2	2.81	Yes	Spain (Madrid)	BOLD:AER0584
					Spain (Madrid)	BOLD:AET3713
9	*Anthophoraplumipes* (Pallas, 1772)	2	5.86	No	Portugal (Coimbra), Spain (Cádiz)	BOLD:AEN8725
					Portugal (Braga)	BOLD:AEO1358
10	*Chelostomacampanularum* (Kirby, 1802)	2	4.25	No	Portugal (Viseu)	BOLD:AAB1123
					Spain (Granada, Málaga)	BOLD:AET7155
11	*Colleteshylaeiformis* Eversmann, 1852	2	4.70	No	Portugal (Castelo Branco)	BOLD:AEU8542
					Spain (Ávila)	BOLD:AEW0951
12	*Flavipanurgusgranadensis* (Warncke, 1987)	2	5.93	No	Spain (Cádiz)	BOLD:AEO4846
					Spain (Murcia)	BOLD:AEO4847
13	*Halictusmaculatus* Smith, 1848	2	3.60	No	Portugal (Porto)	BOLD:ACH4344
					Spain (Granada)	BOLD:AEO0160
14	*Hoplitisravouxi* (Pérez, 1902)	2	2.64	No	Spain (Teruel)	BOLD:AAI1840
					Spain (Málaga)	BOLD:AET2330
15	*Hylaeusangustatus* (Schenck, 1861)	2	6.84%	No	Spain (León, Teruel)	BOLD:AAK3477
					Portugal (Castelo Branco, Viseu)	BOLD:AEN9677
16	*Lasioglossumcalceatum* (Scopoli, 1763)	2	6.55	No	Portugal (Aveiro, Viana do Castelo)	BOLD:AAB0353
					Spain (Málaga)	BOLD:AEO1453
17	*Lasioglossumleucozonium* (Schrank, 1781)	2	6.42	Yes	Portugal (Aveiro)	BOLD:AAA2322
					Portugal (Aveiro, Leiria), Spain (Granada)	BOLD:AEN9620
18	*Lasioglossummalachurum* (Kirby, 1802)	2	13.66	No	Spain (Málaga)	BOLD:AAE5496
					Portugal (Aveiro)	BOLD:AEO8786
19	*Lasioglossumtransitorium* (Schenck, 1868)	2	2.01	No	Portugal (Aveiro), Spain (Cuenca, Granada)	BOLD:AEM8844
					Spain (Lleida)	BOLD:AER6557
20	*Lasioglossumxanthopus* (Kirby, 1802)	2	2.01	No	Spain (Granada)	BOLD:AAE1789
					Portugal (Castelo Branco)	BOLD:AEW1421
21	*Megachilealbisecta* (Klug, 1817)	2	2.17	No	Spain (Granada)	BOLD:AEO5749
					Portugal (Bragança)	BOLD:AEO5750
22	*Nomadabasalis* Herrich-Schäffer, 1839	3	5.71	No	Portugal (Castelo Branco)	BOLD:AEK6178
					Spain (Cuenca)	BOLD:AEN3462
					Portugal (Lisbon), Spain (Madrid, Málaga)	BOLD:AEO4155
23	*Nomadadistinguenda* Morawitz, 1874	2	3.60	No	Portugal (Aveiro)	BOLD:ACY0250
					Portugal (Bragança)	BOLD:AET5764
24	*Nomadafemoralis* Morawitz, 1869	2	7.05	No	Spain (Granada)	BOLD:AAI2830
					Portugal (Aveiro, Faro), Spain (Ávila)	BOLD:AEO6156
25	*Nomadaglaucopis* Pérez, 1890	2	3.11	No	Portugal (Castelo Branco)	BOLD:AEJ1237
					Spain (Granada, Guadalajara, Madrid, Málaga)	BOLD:AEO3423
26	*Nomadaintegra* Brullé, 1832	3	3.43	No	Spain (Ávila)	BOLD:ABZ1320
					Portugal (Castelo Branco), Spain (Málaga)	BOLD:AEN8926
					Spain (Segovia)	BOLD:AEO3904
27	*Sphecodesalternatus* Smith, 1853	2	7.97	Yes	Portugal (Castelo Branco), Spain (Madrid, Granada)	BOLD:ACD7802
					Portugal (Castelo Branco)	BOLD:AEO6580
