# Supplementary material for: The InBIO Barcoding Initiative Database: DNA barcodes of Iberian Bees
Source: Biodivers Data J. 2024 Mar 5;12:e117172. doi: 10.3897/BDJ.12.e117172 (PMC10933585; doi:10.3897/BDJ.12.e117172)
Supplement: Supplementary material 4 — NJ tree of bee DNA barcodes [file bdj-12-e117172-s004.pdf]

# BOLD TaxonID Tree

| | |
|---|---|
| Title | : Tree Result - DS-IBIHY01 (1059 records selected) |
| Date | : 07-Feb-2024 |
| Data Type | : Nucleotide |
| Distance Model | : Kimura 2 Parameter |
| Marker | : COI-5P |
| Colourization | : [blue]=Stop Codons [red]=Contamination or misidentification |

| | |
|---|---|
| Label | : Sample ID |
| Label | : Taxon |
| Label | : Country |
| Label | : Barcode Cluster (BIN) |

| | |
|---|---|
| Sequence Count | : 1058 |
| Species count | : 513 |
| Genus count | : 52 |
| Family count | : 6 |
| Unidentified | : 0 |

| | |
|---|---|
| BIN Count | : 535 |

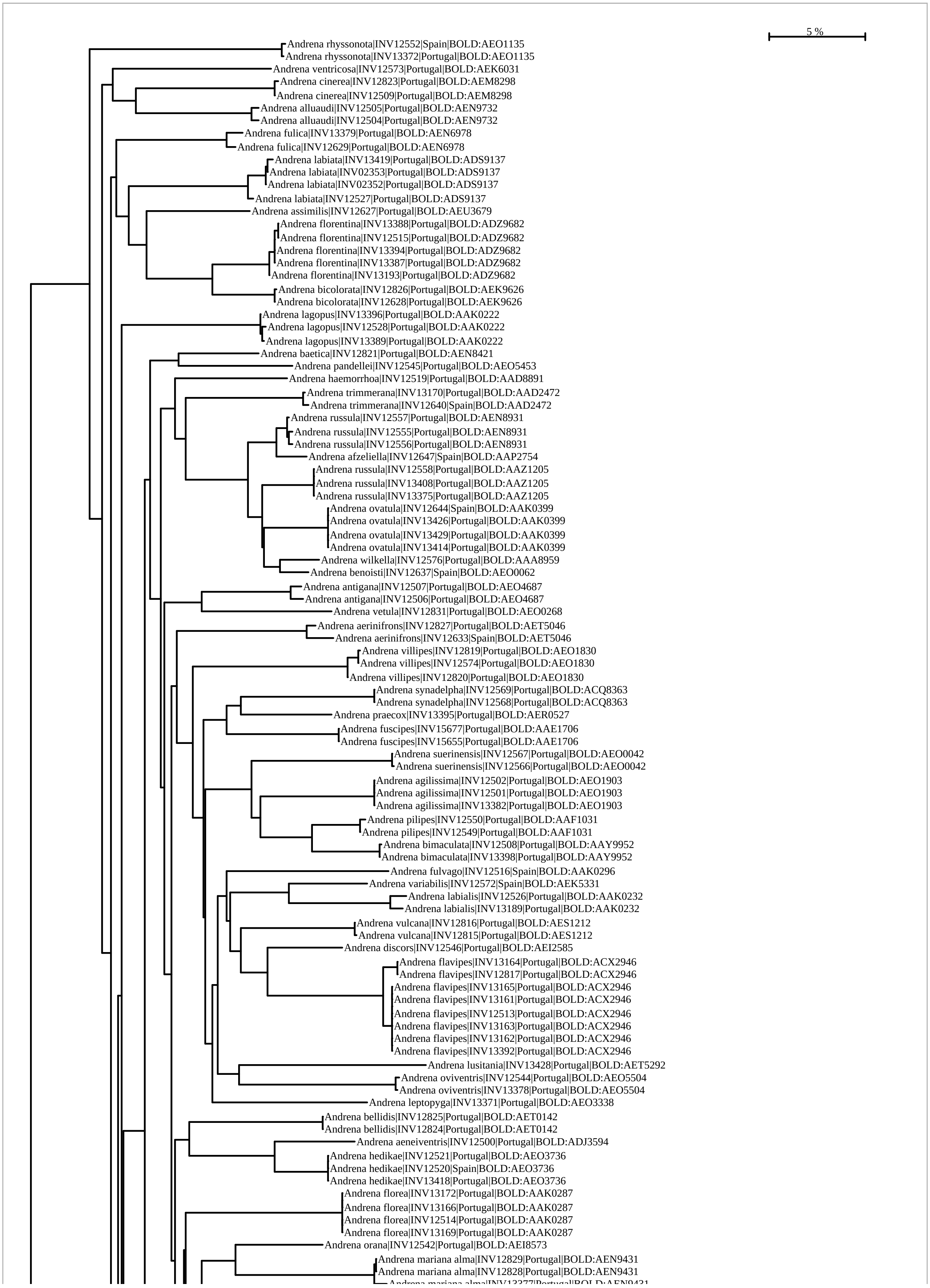
5 %
Andrena rhyssonota|INV12552|Spain|BOLD:AEO1135
Andrena rhyssonota|INV13372|Portugal|BOLD:AEO1135
Andrena ventricosa|INV12573|Portugal|BOLD:AEK6031
Andrena cinerea|INV12823|Portugal|BOLD:AEM8298
Andrena cinerea|INV12509|Portugal|BOLD:AEM8298
Andrena alluaudi|INV12505|Portugal|BOLD:AEN9732
Andrena alluaudi|INV12504|Portugal|BOLD:AEN9732
Andrena fulica|INV13379|Portugal|BOLD:AEN6978
Andrena fulica|INV12629|Portugal|BOLD:AEN6978
Andrena labiata|INV13419|Portugal|BOLD:ADS9137
Andrena labiata|INV02353|Portugal|BOLD:ADS9137
Andrena labiata|INV02352|Portugal|BOLD:ADS9137
Andrena labiata|INV12527|Portugal|BOLD:ADS9137
Andrena assimilis|INV12627|Portugal|BOLD:AEU3679
Andrena florentina|INV13388|Portugal|BOLD:ADZ9682
Andrena florentina|INV12515|Portugal|BOLD:ADZ9682
Andrena florentina|INV13394|Portugal|BOLD:ADZ9682
Andrena florentina|INV13387|Portugal|BOLD:ADZ9682
Andrena florentina|INV13193|Portugal|BOLD:ADZ9682
Andrena bicolorata|INV12826|Portugal|BOLD:AEK9626
Andrena bicolorata|INV12628|Portugal|BOLD:AEK9626
Andrena lagopus|INV13396|Portugal|BOLD:AAK0222
Andrena lagopus|INV12528|Portugal|BOLD:AAK0222
Andrena lagopus|INV13389|Portugal|BOLD:AAK0222
Andrena baetica|INV12821|Portugal|BOLD:AEN8421
Andrena pandellei|INV12545|Portugal|BOLD:AEO5453
Andrena haemorrhoa|INV12519|Portugal|BOLD:AAD8891
Andrena trimmerana|INV13170|Portugal|BOLD:AAD2472
Andrena trimmerana|INV12640|Spain|BOLD:AAD2472
Andrena russula|INV12557|Portugal|BOLD:AEN8931
Andrena russula|INV12555|Portugal|BOLD:AEN8931
Andrena russula|INV12556|Portugal|BOLD:AEN8931
Andrena afzeliella|INV12647|Spain|BOLD:AAP2754
Andrena russula|INV12558|Portugal|BOLD:AAZ1205
Andrena russula|INV13408|Portugal|BOLD:AAZ1205
Andrena russula|INV13375|Portugal|BOLD:AAZ1205
Andrena ovatula|INV12644|Spain|BOLD:AAK0399
Andrena ovatula|INV13426|Portugal|BOLD:AAK0399
Andrena ovatula|INV13429|Portugal|BOLD:AAK0399
Andrena ovatula|INV13414|Portugal|BOLD:AAK0399
Andrena wilkella|INV12576|Portugal|BOLD:AAA8959
Andrena benoisti|INV12637|Spain|BOLD:AEO0062
Andrena antigana|INV12507|Portugal|BOLD:AEO4687
Andrena antigana|INV12506|Portugal|BOLD:AEO4687
Andrena vetula|INV12831|Portugal|BOLD:AEO0268
Andrena aerinifrons|INV12827|Portugal|BOLD:AET5046
Andrena aerinifrons|INV12633|Spain|BOLD:AET5046
Andrena villipes|INV12819|Portugal|BOLD:AEO1830
Andrena villipes|INV12574|Portugal|BOLD:AEO1830
Andrena villipes|INV12820|Portugal|BOLD:AEO1830
Andrena synadelpha|INV12569|Portugal|BOLD:ACQ8363
Andrena synadelpha|INV12568|Portugal|BOLD:ACQ8363
Andrena praecox|INV13395|Portugal|BOLD:AER0527
Andrena fuscipes|INV15677|Portugal|BOLD:AAE1706
Andrena fuscipes|INV15655|Portugal|BOLD:AAE1706
Andrena suerinensis|INV12567|Portugal|BOLD:AEO0042
Andrena suerinensis|INV12566|Portugal|BOLD:AEO0042
Andrena agilissima|INV12502|Portugal|BOLD:AEO1903
Andrena agilissima|INV12501|Portugal|BOLD:AEO1903
Andrena agilissima|INV13382|Portugal|BOLD:AEO1903
Andrena pilipes|INV12550|Portugal|BOLD:AAF1031
Andrena pilipes|INV12549|Portugal|BOLD:AAF1031
Andrena bimaculata|INV12508|Portugal|BOLD:AAY9952
Andrena bimaculata|INV13398|Portugal|BOLD:AAY9952
Andrena fulvago|INV12516|Spain|BOLD:AAK0296
Andrena variabilis|INV12572|Spain|BOLD:AEK5331
Andrena labialis|INV12526|Portugal|BOLD:AAK0232
Andrena labialis|INV13189|Portugal|BOLD:AAK0232
Andrena vulcana|INV12816|Portugal|BOLD:AES1212
Andrena vulcana|INV12815|Portugal|BOLD:AES1212
Andrena discors|INV12546|Portugal|BOLD:AEI2585
Andrena flavipes|INV13164|Portugal|BOLD:ACX2946
Andrena flavipes|INV12817|Portugal|BOLD:ACX2946
Andrena flavipes|INV13165|Portugal|BOLD:ACX2946
Andrena flavipes|INV13161|Portugal|BOLD:ACX2946
Andrena flavipes|INV12513|Portugal|BOLD:ACX2946
Andrena flavipes|INV13163|Portugal|BOLD:ACX2946
Andrena flavipes|INV13162|Portugal|BOLD:ACX2946
Andrena flavipes|INV13392|Portugal|BOLD:ACX2946
Andrena lusitania|INV13428|Portugal|BOLD:AET5292
Andrena oviventris|INV12544|Portugal|BOLD:AEO5504
Andrena oviventris|INV13378|Portugal|BOLD:AEO5504
Andrena leptopyga|INV13371|Portugal|BOLD:AEO3338
Andrena bellidis|INV12825|Portugal|BOLD:AET0142
Andrena bellidis|INV12824|Portugal|BOLD:AET0142
Andrena aeneiventris|INV12500|Portugal|BOLD:ADJ3594
Andrena hedikae|INV12521|Portugal|BOLD:AEO3736
Andrena hedikae|INV12520|Spain|BOLD:AEO3736
Andrena hedikae|INV13418|Portugal|BOLD:AEO3736
Andrena florea|INV13172|Portugal|BOLD:AAK0287
Andrena florea|INV13166|Portugal|BOLD:AAK0287
Andrena florea|INV12514|Portugal|BOLD:AAK0287
Andrena florea|INV13169|Portugal|BOLD:AAK0287
Andrena orana|INV12542|Portugal|BOLD:AEI8573
Andrena mariana alma|INV12829|Portugal|BOLD:AEN9431
Andrena mariana alma|INV12828|Portugal|BOLD:AEN9431

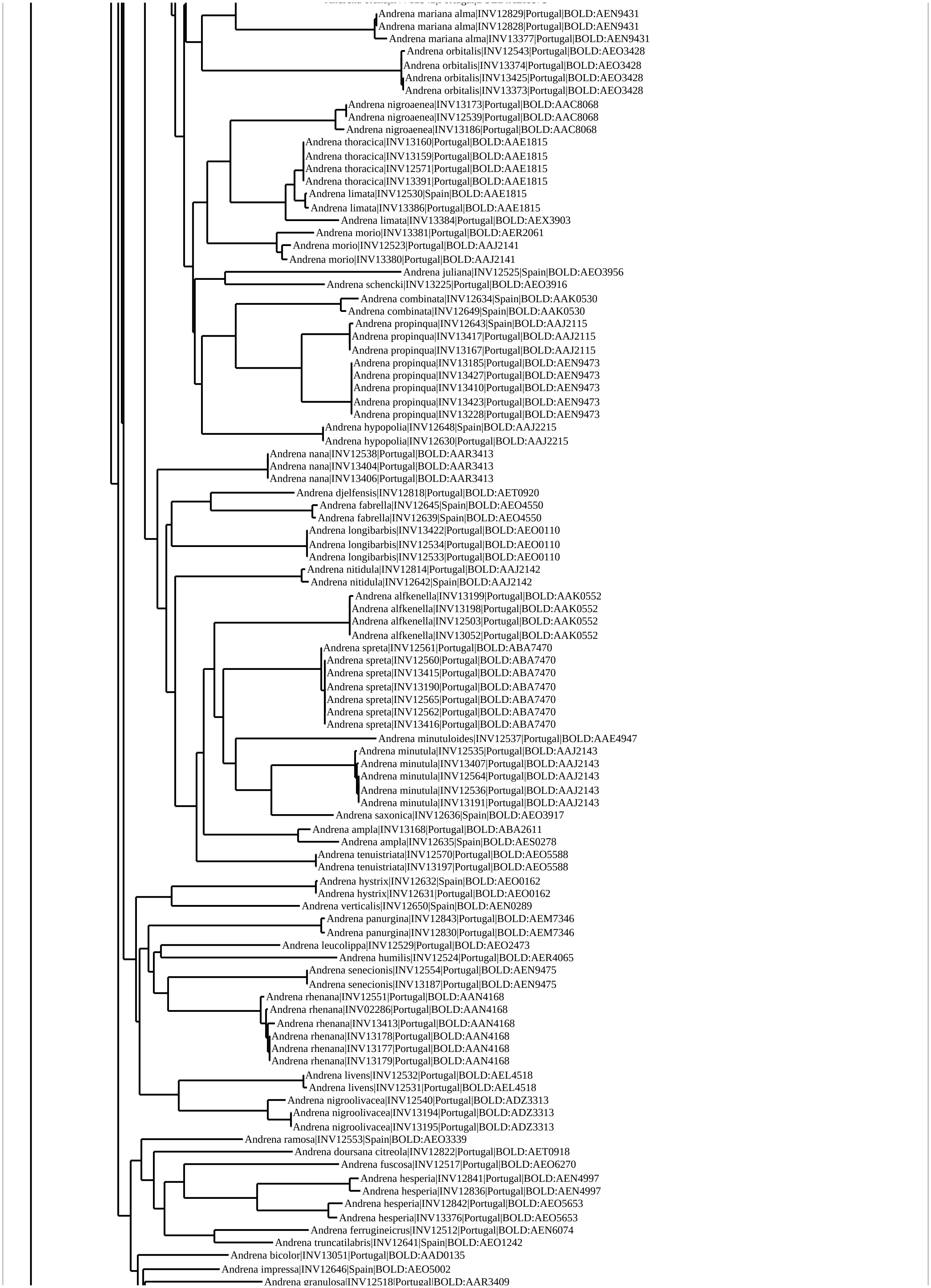
Andrena mariana alma|INV12829|Portugal|BOLD:AEN9431
Andrena mariana alma|INV12828|Portugal|BOLD:AEN9431
Andrena mariana alma|INV13377|Portugal|BOLD:AEN9431
Andrena orbitalis|INV12543|Portugal|BOLD:AEO3428
Andrena orbitalis|INV13374|Portugal|BOLD:AEO3428
Andrena orbitalis|INV13425|Portugal|BOLD:AEO3428
Andrena orbitalis|INV13373|Portugal|BOLD:AEO3428
Andrena nigroaenea|INV13173|Portugal|BOLD:AAC8068
Andrena nigroaenea|INV12539|Portugal|BOLD:AAC8068
Andrena nigroaenea|INV13186|Portugal|BOLD:AAC8068
Andrena thoracica|INV13160|Portugal|BOLD:AAE1815
Andrena thoracica|INV13159|Portugal|BOLD:AAE1815
Andrena thoracica|INV12571|Portugal|BOLD:AAE1815
Andrena thoracica|INV13391|Portugal|BOLD:AAE1815
Andrena limata|INV12530|Spain|BOLD:AAE1815
Andrena limata|INV13386|Portugal|BOLD:AAE1815
Andrena limata|INV13384|Portugal|BOLD:AEX3903
Andrena morio|INV13381|Portugal|BOLD:AER2061
Andrena morio|INV12523|Portugal|BOLD:AAJ2141
Andrena morio|INV13380|Portugal|BOLD:AAJ2141
Andrena juliana|INV12525|Spain|BOLD:AEO3956
Andrena schencki|INV13225|Portugal|BOLD:AEO3916
Andrena combinata|INV12634|Spain|BOLD:AAK0530
Andrena combinata|INV12649|Spain|BOLD:AAK0530
Andrena propinqua|INV12643|Spain|BOLD:AAJ2115
Andrena propinqua|INV13417|Portugal|BOLD:AAJ2115
Andrena propinqua|INV13167|Portugal|BOLD:AAJ2115
Andrena propinqua|INV13185|Portugal|BOLD:AEN9473
Andrena propinqua|INV13427|Portugal|BOLD:AEN9473
Andrena propinqua|INV13410|Portugal|BOLD:AEN9473
Andrena propinqua|INV13423|Portugal|BOLD:AEN9473
Andrena propinqua|INV13228|Portugal|BOLD:AEN9473
Andrena hypopolia|INV12648|Spain|BOLD:AAJ2215
Andrena hypopolia|INV12630|Portugal|BOLD:AAJ2215
Andrena nana|INV12538|Portugal|BOLD:AAR3413
Andrena nana|INV13404|Portugal|BOLD:AAR3413
Andrena nana|INV13406|Portugal|BOLD:AAR3413
Andrena djelfensis|INV12818|Portugal|BOLD:AET0920
Andrena fabrella|INV12645|Spain|BOLD:AEO4550
Andrena fabrella|INV12639|Spain|BOLD:AEO4550
Andrena longibarbis|INV13422|Portugal|BOLD:AEO0110
Andrena longibarbis|INV12534|Portugal|BOLD:AEO0110
Andrena longibarbis|INV12533|Portugal|BOLD:AEO0110
Andrena nitidula|INV12814|Portugal|BOLD:AAJ2142
Andrena nitidula|INV12642|Spain|BOLD:AAJ2142
Andrena alfkenella|INV13199|Portugal|BOLD:AAK0552
Andrena alfkenella|INV13198|Portugal|BOLD:AAK0552
Andrena alfkenella|INV12503|Portugal|BOLD:AAK0552
Andrena alfkenella|INV13052|Portugal|BOLD:AAK0552
Andrena spreta|INV12561|Portugal|BOLD:ABA7470
Andrena spreta|INV12560|Portugal|BOLD:ABA7470
Andrena spreta|INV13415|Portugal|BOLD:ABA7470
Andrena spreta|INV13190|Portugal|BOLD:ABA7470
Andrena spreta|INV12565|Portugal|BOLD:ABA7470
Andrena spreta|INV12562|Portugal|BOLD:ABA7470
Andrena spreta|INV13416|Portugal|BOLD:ABA7470
Andrena minutuloides|INV12537|Portugal|BOLD:AAE4947
Andrena minutula|INV12535|Portugal|BOLD:AAJ2143
Andrena minutula|INV13407|Portugal|BOLD:AAJ2143
Andrena minutula|INV12564|Portugal|BOLD:AAJ2143
Andrena minutula|INV12536|Portugal|BOLD:AAJ2143
Andrena minutula|INV13191|Portugal|BOLD:AAJ2143
Andrena saxonica|INV12636|Spain|BOLD:AEO3917
Andrena ampla|INV13168|Portugal|BOLD:ABA2611
Andrena ampla|INV12635|Spain|BOLD:AES0278
Andrena tenuistriata|INV12570|Portugal|BOLD:AEO5588
Andrena tenuistriata|INV13197|Portugal|BOLD:AEO5588
Andrena hystrix|INV12632|Spain|BOLD:AEO0162
Andrena hystrix|INV12631|Portugal|BOLD:AEO0162
Andrena verticalis|INV12650|Spain|BOLD:AEN0289
Andrena panurgina|INV12843|Portugal|BOLD:AEM7346
Andrena panurgina|INV12830|Portugal|BOLD:AEM7346
Andrena leucolippa|INV12529|Portugal|BOLD:AEO2473
Andrena humilis|INV12524|Portugal|BOLD:AER4065
Andrena senecionis|INV12554|Portugal|BOLD:AEN9475
Andrena senecionis|INV13187|Portugal|BOLD:AEN9475
Andrena rhenana|INV12551|Portugal|BOLD:AAN4168
Andrena rhenana|INV02286|Portugal|BOLD:AAN4168
Andrena rhenana|INV13413|Portugal|BOLD:AAN4168
Andrena rhenana|INV13178|Portugal|BOLD:AAN4168
Andrena rhenana|INV13177|Portugal|BOLD:AAN4168
Andrena rhenana|INV13179|Portugal|BOLD:AAN4168
Andrena livens|INV12532|Portugal|BOLD:AEL4518
Andrena livens|INV12531|Portugal|BOLD:AEL4518
Andrena nigroolivacea|INV12540|Portugal|BOLD:ADZ3313
Andrena nigroolivacea|INV13194|Portugal|BOLD:ADZ3313
Andrena nigroolivacea|INV13195|Portugal|BOLD:ADZ3313
Andrena ramosa|INV12553|Spain|BOLD:AEO3339
Andrena doursana citreola|INV12822|Portugal|BOLD:AET0918
Andrena fuscosa|INV12517|Portugal|BOLD:AEO6270
Andrena hesperia|INV12841|Portugal|BOLD:AEN4997
Andrena hesperia|INV12836|Portugal|BOLD:AEN4997
Andrena hesperia|INV12842|Portugal|BOLD:AEO5653
Andrena hesperia|INV13376|Portugal|BOLD:AEO5653
Andrena ferrugineicrus|INV12512|Portugal|BOLD:AEN6074
Andrena truncatilabris|INV12641|Spain|BOLD:AEO1242
Andrena bicolor|INV13051|Portugal|BOLD:AAD0135
Andrena impressa|INV12646|Spain|BOLD:AEO5002
Andrena granulosa|INV12518|Portugal|BOLD:AAR3409

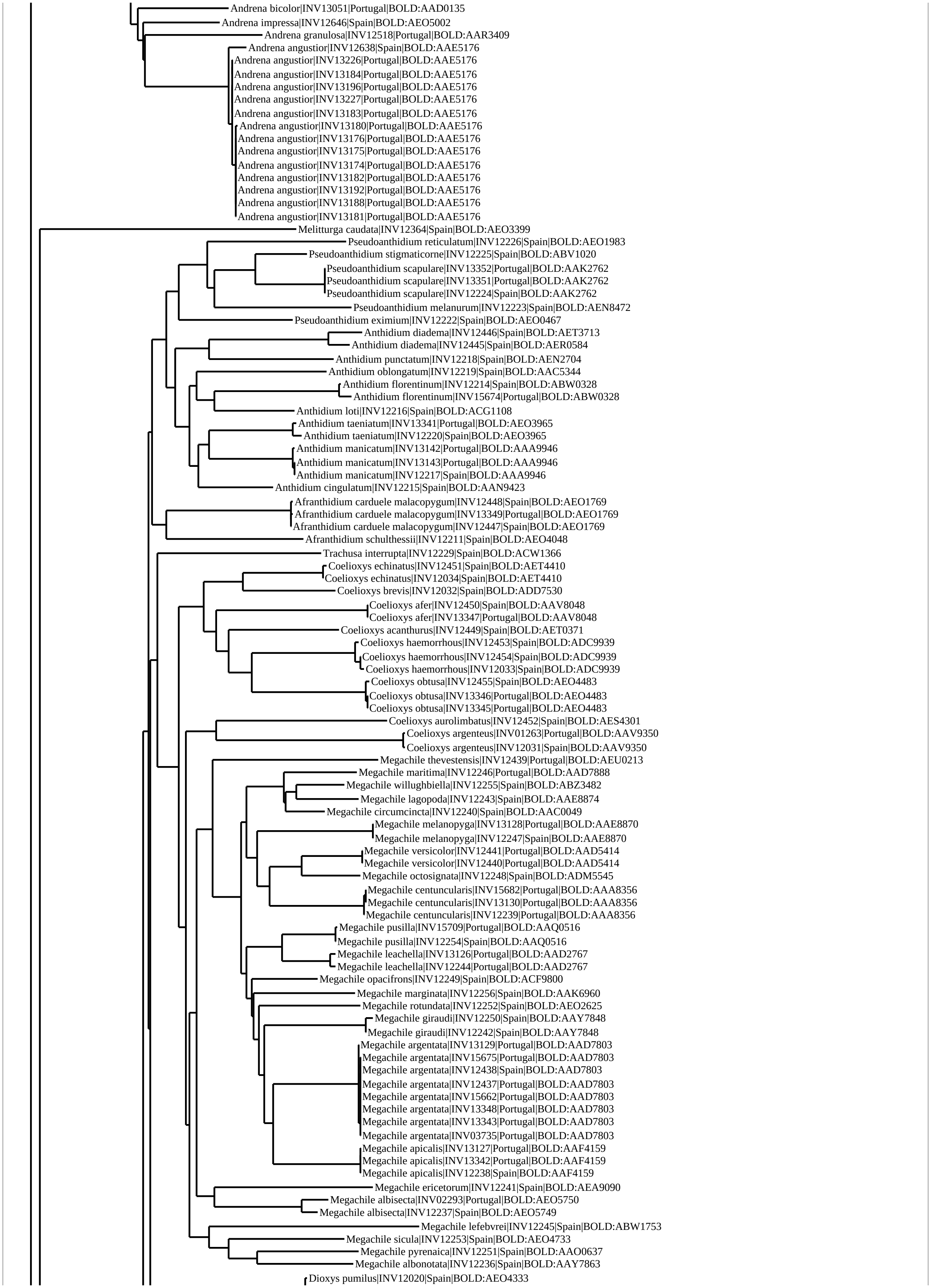
Andrena bicolor|INV13051|Portugal|BOLD:AAD0135
Andrena impressa|INV12646|Spain|BOLD:AEO5002
Andrena granulosa|INV12518|Portugal|BOLD:AAR3409
Andrena angustior|INV12638|Spain|BOLD:AAE5176
Andrena angustior|INV13226|Portugal|BOLD:AAE5176
Andrena angustior|INV13184|Portugal|BOLD:AAE5176
Andrena angustior|INV13196|Portugal|BOLD:AAE5176
Andrena angustior|INV13227|Portugal|BOLD:AAE5176
Andrena angustior|INV13183|Portugal|BOLD:AAE5176
Andrena angustior|INV13180|Portugal|BOLD:AAE5176
Andrena angustior|INV13176|Portugal|BOLD:AAE5176
Andrena angustior|INV13175|Portugal|BOLD:AAE5176
Andrena angustior|INV13174|Portugal|BOLD:AAE5176
Andrena angustior|INV13182|Portugal|BOLD:AAE5176
Andrena angustior|INV13192|Portugal|BOLD:AAE5176
Andrena angustior|INV13188|Portugal|BOLD:AAE5176
Andrena angustior|INV13181|Portugal|BOLD:AAE5176
Melitturga caudata|INV12364|Spain|BOLD:AEO3399
Pseudoanthidium reticulatum|INV12226|Spain|BOLD:AEO1983
Pseudoanthidium stigmaticorne|INV12225|Spain|BOLD:ABV1020
Pseudoanthidium scapulare|INV13352|Portugal|BOLD:AAK2762
Pseudoanthidium scapulare|INV13351|Portugal|BOLD:AAK2762
Pseudoanthidium scapulare|INV12224|Spain|BOLD:AAK2762
Pseudoanthidium melanurum|INV12223|Spain|BOLD:AEN8472
Pseudoanthidium eximium|INV12222|Spain|BOLD:AEO0467
Anthidium diadema|INV12446|Spain|BOLD:AET3713
Anthidium diadema|INV12445|Spain|BOLD:AER0584
Anthidium punctatum|INV12218|Spain|BOLD:AEN2704
Anthidium oblongatum|INV12219|Spain|BOLD:AAC5344
Anthidium florentinum|INV12214|Spain|BOLD:ABW0328
Anthidium florentinum|INV15674|Portugal|BOLD:ABW0328
Anthidium loti|INV12216|Spain|BOLD:ACG1108
Anthidium taeniatum|INV13341|Portugal|BOLD:AEO3965
Anthidium taeniatum|INV12220|Spain|BOLD:AEO3965
Anthidium manicatum|INV13142|Portugal|BOLD:AAA9946
Anthidium manicatum|INV13143|Portugal|BOLD:AAA9946
Anthidium manicatum|INV12217|Spain|BOLD:AAA9946
Anthidium cingulatum|INV12215|Spain|BOLD:AAN9423
Afranthidium carduele malacopygum|INV12448|Spain|BOLD:AEO1769
Afranthidium carduele malacopygum|INV13349|Portugal|BOLD:AEO1769
Afranthidium carduele malacopygum|INV12447|Spain|BOLD:AEO1769
Afranthidium schulthessii|INV12211|Spain|BOLD:AEO4048
Trachusa interrupta|INV12229|Spain|BOLD:ACW1366
Coelioxys echinatus|INV12451|Spain|BOLD:AET4410
Coelioxys echinatus|INV12034|Spain|BOLD:AET4410
Coelioxys brevis|INV12032|Spain|BOLD:ADD7530
Coelioxys afer|INV12450|Spain|BOLD:AAV8048
Coelioxys afer|INV13347|Portugal|BOLD:AAV8048
Coelioxys acanthurus|INV12449|Spain|BOLD:AET0371
Coelioxys haemorrhous|INV12453|Spain|BOLD:ADC9939
Coelioxys haemorrhous|INV12454|Spain|BOLD:ADC9939
Coelioxys haemorrhous|INV12033|Spain|BOLD:ADC9939
Coelioxys obtusa|INV12455|Spain|BOLD:AEO4483
Coelioxys obtusa|INV13346|Portugal|BOLD:AEO4483
Coelioxys obtusa|INV13345|Portugal|BOLD:AEO4483
Coelioxys aurolimbatus|INV12452|Spain|BOLD:AES4301
Coelioxys argenteus|INV01263|Portugal|BOLD:AAV9350
Coelioxys argenteus|INV12031|Spain|BOLD:AAV9350
Megachile thevestensis|INV12439|Portugal|BOLD:AEU0213
Megachile maritima|INV12246|Portugal|BOLD:AAD7888
Megachile willughbiella|INV12255|Spain|BOLD:ABZ3482
Megachile lagopoda|INV12243|Spain|BOLD:AAE8874
Megachile circumcincta|INV12240|Spain|BOLD:AAC0049
Megachile melanopyga|INV13128|Portugal|BOLD:AAE8870
Megachile melanopyga|INV12247|Spain|BOLD:AAE8870
Megachile versicolor|INV12441|Portugal|BOLD:AAD5414
Megachile versicolor|INV12440|Portugal|BOLD:AAD5414
Megachile octosignata|INV12248|Spain|BOLD:ADM5545
Megachile centuncularis|INV15682|Portugal|BOLD:AAA8356
Megachile centuncularis|INV13130|Portugal|BOLD:AAA8356
Megachile centuncularis|INV12239|Portugal|BOLD:AAA8356
Megachile pusilla|INV15709|Portugal|BOLD:AAQ0516
Megachile pusilla|INV12254|Spain|BOLD:AAQ0516
Megachile leachella|INV13126|Portugal|BOLD:AAD2767
Megachile leachella|INV12244|Portugal|BOLD:AAD2767
Megachile opacifrons|INV12249|Spain|BOLD:ACF9800
Megachile marginata|INV12256|Spain|BOLD:AAK6960
Megachile rotundata|INV12252|Spain|BOLD:AEO2625
Megachile giraudi|INV12250|Spain|BOLD:AAY7848
Megachile giraudi|INV12242|Spain|BOLD:AAY7848
Megachile argentata|INV13129|Portugal|BOLD:AAD7803
Megachile argentata|INV15675|Portugal|BOLD:AAD7803
Megachile argentata|INV12438|Spain|BOLD:AAD7803
Megachile argentata|INV12437|Portugal|BOLD:AAD7803
Megachile argentata|INV15662|Portugal|BOLD:AAD7803
Megachile argentata|INV13348|Portugal|BOLD:AAD7803
Megachile argentata|INV13343|Portugal|BOLD:AAD7803
Megachile argentata|INV03735|Portugal|BOLD:AAD7803
Megachile apicalis|INV13127|Portugal|BOLD:AAF4159
Megachile apicalis|INV13342|Portugal|BOLD:AAF4159
Megachile apicalis|INV12238|Spain|BOLD:AAF4159
Megachile ericetorum|INV12241|Spain|BOLD:AEA9090
Megachile albisecta|INV02293|Portugal|BOLD:AEO5750
Megachile albisecta|INV12237|Spain|BOLD:AEO5749
Megachile lefebvrei|INV12245|Spain|BOLD:ABW1753
Megachile sicula|INV12253|Spain|BOLD:AEO4733
Megachile pyrenaica|INV12251|Spain|BOLD:AAO0637
Megachile albonotata|INV12236|Spain|BOLD:AAY7863
Dioxys pumilus|INV12020|Spain|BOLD:AEO4333

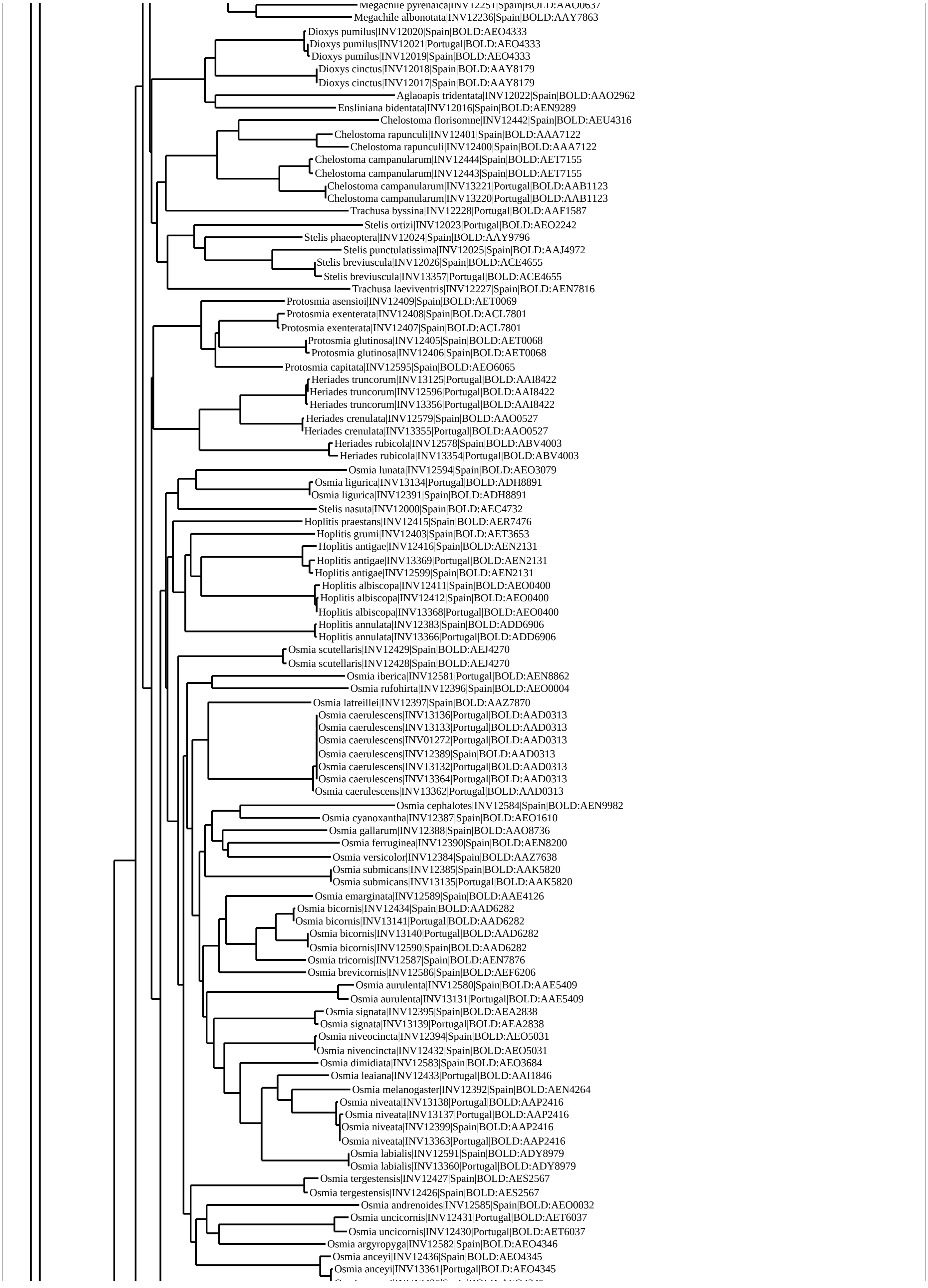
Megachile pyrenaica|INV12251|Spain|BOLD:AAO0637
Megachile albonotata|INV12236|Spain|BOLD:AAY7863
Dioxys pumilus|INV12020|Spain|BOLD:AEO4333
Dioxys pumilus|INV12021|Portugal|BOLD:AEO4333
Dioxys pumilus|INV12019|Spain|BOLD:AEO4333
Dioxys cinctus|INV12018|Spain|BOLD:AAY8179
Dioxys cinctus|INV12017|Spain|BOLD:AAY8179
Aglaoapis tridentata|INV12022|Spain|BOLD:AAO2962
Ensliniana bidentata|INV12016|Spain|BOLD:AEN9289
Chelostoma florisomne|INV12442|Spain|BOLD:AEU4316
Chelostoma rapunculi|INV12401|Spain|BOLD:AAA7122
Chelostoma rapunculi|INV12400|Spain|BOLD:AAA7122
Chelostoma campanularum|INV12444|Spain|BOLD:AET7155
Chelostoma campanularum|INV12443|Spain|BOLD:AET7155
Chelostoma campanularum|INV13221|Portugal|BOLD:AAB1123
Chelostoma campanularum|INV13220|Portugal|BOLD:AAB1123
Trachusa byssina|INV12228|Portugal|BOLD:AAF1587
Stelis ortizi|INV12023|Portugal|BOLD:AEO2242
Stelis phaeoptera|INV12024|Spain|BOLD:AAY9796
Stelis punctulatissima|INV12025|Spain|BOLD:AAJ4972
Stelis breviuscula|INV12026|Spain|BOLD:ACE4655
Stelis breviuscula|INV13357|Portugal|BOLD:ACE4655
Trachusa laeviventris|INV12227|Spain|BOLD:AEN7816
Protosmia asensioi|INV12409|Spain|BOLD:AET0069
Protosmia exenterata|INV12408|Spain|BOLD:ACL7801
Protosmia exenterata|INV12407|Spain|BOLD:ACL7801
Protosmia glutinosa|INV12405|Spain|BOLD:AET0068
Protosmia glutinosa|INV12406|Spain|BOLD:AET0068
Protosmia capitata|INV12595|Spain|BOLD:AEO6065
Heriades truncorum|INV13125|Portugal|BOLD:AAI8422
Heriades truncorum|INV12596|Portugal|BOLD:AAI8422
Heriades truncorum|INV13356|Portugal|BOLD:AAI8422
Heriades crenulata|INV12579|Spain|BOLD:AAO0527
Heriades crenulata|INV13355|Portugal|BOLD:AAO0527
Heriades rubicola|INV12578|Spain|BOLD:ABV4003
Heriades rubicola|INV13354|Portugal|BOLD:ABV4003
Osmia lunata|INV12594|Spain|BOLD:AEO3079
Osmia ligurica|INV13134|Portugal|BOLD:ADH8891
Osmia ligurica|INV12391|Spain|BOLD:ADH8891
Stelis nasuta|INV12000|Spain|BOLD:AEC4732
Hoplitis praestans|INV12415|Spain|BOLD:AER7476
Hoplitis grumi|INV12403|Spain|BOLD:AET3653
Hoplitis antigae|INV12416|Spain|BOLD:AEN2131
Hoplitis antigae|INV13369|Portugal|BOLD:AEN2131
Hoplitis antigae|INV12599|Spain|BOLD:AEN2131
Hoplitis albiscopa|INV12411|Spain|BOLD:AEO0400
Hoplitis albiscopa|INV12412|Spain|BOLD:AEO0400
Hoplitis albiscopa|INV13368|Portugal|BOLD:AEO0400
Hoplitis annulata|INV12383|Spain|BOLD:ADD6906
Hoplitis annulata|INV13366|Portugal|BOLD:ADD6906
Osmia scutellaris|INV12429|Spain|BOLD:AEJ4270
Osmia scutellaris|INV12428|Spain|BOLD:AEJ4270
Osmia iberica|INV12581|Portugal|BOLD:AEN8862
Osmia rufohirta|INV12396|Spain|BOLD:AEO0004
Osmia latreillei|INV12397|Spain|BOLD:AAZ7870
Osmia caerulescens|INV13136|Portugal|BOLD:AAD0313
Osmia caerulescens|INV13133|Portugal|BOLD:AAD0313
Osmia caerulescens|INV01272|Portugal|BOLD:AAD0313
Osmia caerulescens|INV12389|Spain|BOLD:AAD0313
Osmia caerulescens|INV13132|Portugal|BOLD:AAD0313
Osmia caerulescens|INV13364|Portugal|BOLD:AAD0313
Osmia caerulescens|INV13362|Portugal|BOLD:AAD0313
Osmia cephalotes|INV12584|Spain|BOLD:AEN9982
Osmia cyanoxantha|INV12387|Spain|BOLD:AEO1610
Osmia gallarum|INV12388|Spain|BOLD:AAO8736
Osmia ferruginea|INV12390|Spain|BOLD:AEN8200
Osmia versicolor|INV12384|Spain|BOLD:AAZ7638
Osmia submicans|INV12385|Spain|BOLD:AAK5820
Osmia submicans|INV13135|Portugal|BOLD:AAK5820
Osmia emarginata|INV12589|Spain|BOLD:AAE4126
Osmia bicornis|INV12434|Spain|BOLD:AAD6282
Osmia bicornis|INV13141|Portugal|BOLD:AAD6282
Osmia bicornis|INV13140|Portugal|BOLD:AAD6282
Osmia bicornis|INV12590|Spain|BOLD:AAD6282
Osmia tricornis|INV12587|Spain|BOLD:AEN7876
Osmia brevicornis|INV12586|Spain|BOLD:AEF6206
Osmia aurulenta|INV12580|Spain|BOLD:AAE5409
Osmia aurulenta|INV13131|Portugal|BOLD:AAE5409
Osmia signata|INV12395|Spain|BOLD:AEA2838
Osmia signata|INV13139|Portugal|BOLD:AEA2838
Osmia niveocincta|INV12394|Spain|BOLD:AEO5031
Osmia niveocincta|INV12432|Spain|BOLD:AEO5031
Osmia dimidiata|INV12583|Spain|BOLD:AEO3684
Osmia leaiana|INV12433|Portugal|BOLD:AAI1846
Osmia melanogaster|INV12392|Spain|BOLD:AEN4264
Osmia niveata|INV13138|Portugal|BOLD:AAP2416
Osmia niveata|INV13137|Portugal|BOLD:AAP2416
Osmia niveata|INV12399|Spain|BOLD:AAP2416
Osmia niveata|INV13363|Portugal|BOLD:AAP2416
Osmia labialis|INV12591|Spain|BOLD:ADY8979
Osmia labialis|INV13360|Portugal|BOLD:ADY8979
Osmia tergestensis|INV12427|Spain|BOLD:AES2567
Osmia tergestensis|INV12426|Spain|BOLD:AES2567
Osmia andrenoides|INV12585|Spain|BOLD:AEO0032
Osmia uncicornis|INV12431|Portugal|BOLD:AET6037
Osmia uncicornis|INV12430|Portugal|BOLD:AET6037
Osmia argyropyga|INV12582|Spain|BOLD:AEO4346
Osmia anceyi|INV12436|Spain|BOLD:AEO4345
Osmia anceyi|INV13361|Portugal|BOLD:AEO4345

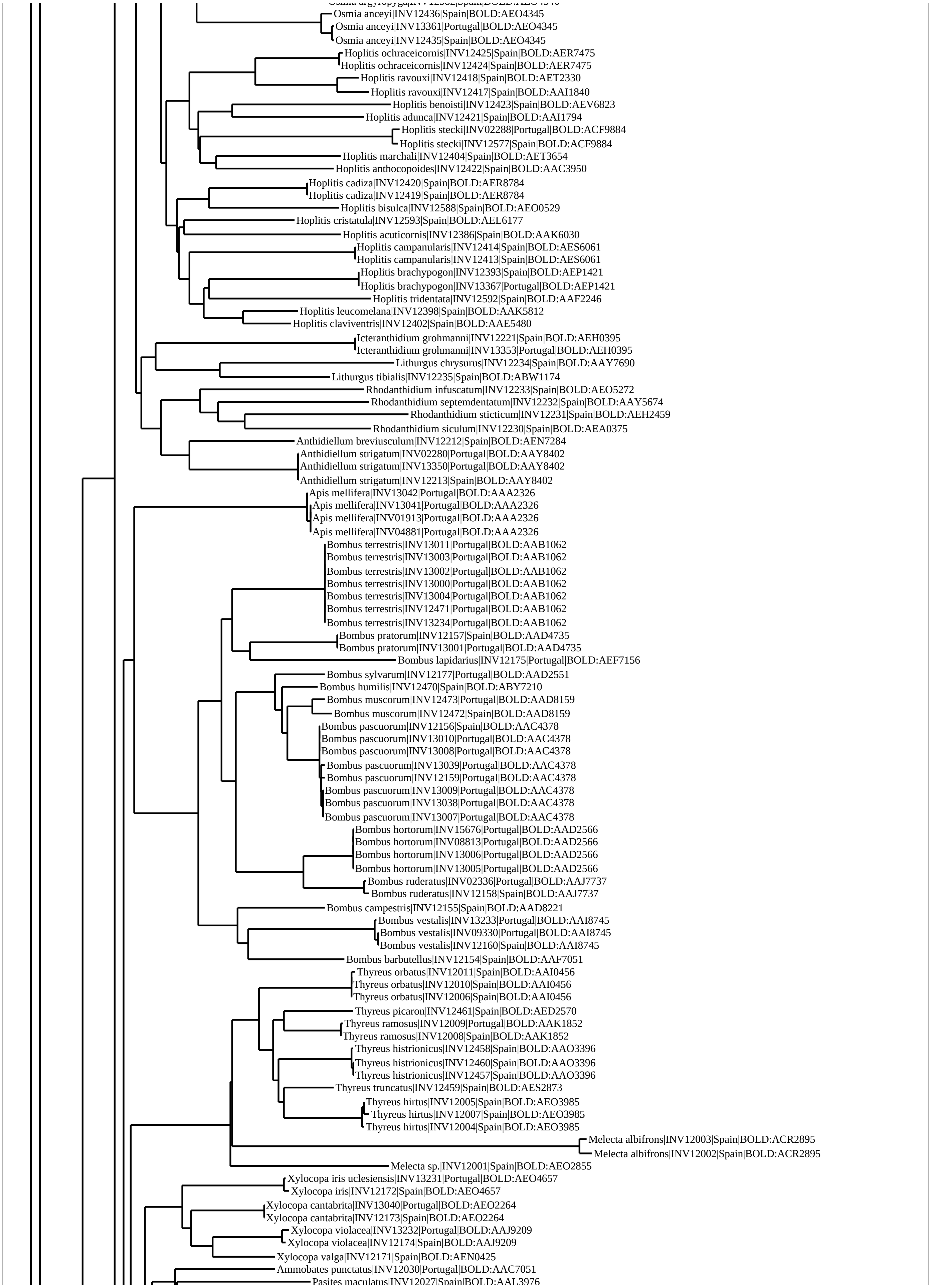
Osmia anceyi|INV12436|Spain|BOLD:AEO4345
Osmia anceyi|INV13361|Portugal|BOLD:AEO4345
Osmia anceyi|INV12435|Spain|BOLD:AEO4345
Hoplitis ochraceicornis|INV12425|Spain|BOLD:AER7475
Hoplitis ochraceicornis|INV12424|Spain|BOLD:AER7475
Hoplitis ravouxi|INV12418|Spain|BOLD:AET2330
Hoplitis ravouxi|INV12417|Spain|BOLD:AAI1840
Hoplitis benoisti|INV12423|Spain|BOLD:AEV6823
Hoplitis adunca|INV12421|Spain|BOLD:AAI1794
Hoplitis stecki|INV02288|Portugal|BOLD:ACF9884
Hoplitis stecki|INV12577|Spain|BOLD:ACF9884
Hoplitis marchali|INV12404|Spain|BOLD:AET3654
Hoplitis anthocopoides|INV12422|Spain|BOLD:AAC3950
Hoplitis cadiza|INV12420|Spain|BOLD:AER8784
Hoplitis cadiza|INV12419|Spain|BOLD:AER8784
Hoplitis bisulca|INV12588|Spain|BOLD:AEO0529
Hoplitis cristatula|INV12593|Spain|BOLD:AEL6177
Hoplitis acuticornis|INV12386|Spain|BOLD:AAK6030
Hoplitis campanularis|INV12414|Spain|BOLD:AES6061
Hoplitis campanularis|INV12413|Spain|BOLD:AES6061
Hoplitis brachypogon|INV12393|Spain|BOLD:AEP1421
Hoplitis brachypogon|INV13367|Portugal|BOLD:AEP1421
Hoplitis tridentata|INV12592|Spain|BOLD:AAF2246
Hoplitis leucomelana|INV12398|Spain|BOLD:AAK5812
Hoplitis claviventris|INV12402|Spain|BOLD:AAE5480
Icteranthidium grohmanni|INV12221|Spain|BOLD:AEH0395
Icteranthidium grohmanni|INV13353|Portugal|BOLD:AEH0395
Lithurgus chrysurus|INV12234|Spain|BOLD:AAY7690
Lithurgus tibialis|INV12235|Spain|BOLD:ABW1174
Rhodanthidium infuscatum|INV12233|Spain|BOLD:AEO5272
Rhodanthidium septemdentatum|INV12232|Spain|BOLD:AAY5674
Rhodanthidium sticticum|INV12231|Spain|BOLD:AEH2459
Rhodanthidium siculum|INV12230|Spain|BOLD:AEA0375
Anthidiellum breviusculum|INV12212|Spain|BOLD:AEN7284
Anthidiellum strigatum|INV02280|Portugal|BOLD:AAY8402
Anthidiellum strigatum|INV13350|Portugal|BOLD:AAY8402
Anthidiellum strigatum|INV12213|Spain|BOLD:AAY8402
Apis mellifera|INV13042|Portugal|BOLD:AAA2326
Apis mellifera|INV13041|Portugal|BOLD:AAA2326
Apis mellifera|INV01913|Portugal|BOLD:AAA2326
Apis mellifera|INV04881|Portugal|BOLD:AAA2326
Bombus terrestris|INV13011|Portugal|BOLD:AAB1062
Bombus terrestris|INV13003|Portugal|BOLD:AAB1062
Bombus terrestris|INV13002|Portugal|BOLD:AAB1062
Bombus terrestris|INV13000|Portugal|BOLD:AAB1062
Bombus terrestris|INV13004|Portugal|BOLD:AAB1062
Bombus terrestris|INV12471|Portugal|BOLD:AAB1062
Bombus terrestris|INV13234|Portugal|BOLD:AAB1062
Bombus pratorum|INV12157|Spain|BOLD:AAD4735
Bombus pratorum|INV13001|Portugal|BOLD:AAD4735
Bombus lapidarius|INV12175|Portugal|BOLD:AEF7156
Bombus sylvarum|INV12177|Portugal|BOLD:AAD2551
Bombus humilis|INV12470|Spain|BOLD:ABY7210
Bombus muscorum|INV12473|Portugal|BOLD:AAD8159
Bombus muscorum|INV12472|Spain|BOLD:AAD8159
Bombus pascuorum|INV12156|Spain|BOLD:AAC4378
Bombus pascuorum|INV13010|Portugal|BOLD:AAC4378
Bombus pascuorum|INV13008|Portugal|BOLD:AAC4378
Bombus pascuorum|INV13039|Portugal|BOLD:AAC4378
Bombus pascuorum|INV12159|Portugal|BOLD:AAC4378
Bombus pascuorum|INV13009|Portugal|BOLD:AAC4378
Bombus pascuorum|INV13038|Portugal|BOLD:AAC4378
Bombus pascuorum|INV13007|Portugal|BOLD:AAC4378
Bombus hortorum|INV15676|Portugal|BOLD:AAD2566
Bombus hortorum|INV08813|Portugal|BOLD:AAD2566
Bombus hortorum|INV13006|Portugal|BOLD:AAD2566
Bombus hortorum|INV13005|Portugal|BOLD:AAD2566
Bombus ruderatus|INV02336|Portugal|BOLD:AAJ7737
Bombus ruderatus|INV12158|Spain|BOLD:AAJ7737
Bombus campestris|INV12155|Spain|BOLD:AAD8221
Bombus vestalis|INV13233|Portugal|BOLD:AAI8745
Bombus vestalis|INV09330|Portugal|BOLD:AAI8745
Bombus vestalis|INV12160|Spain|BOLD:AAI8745
Bombus barbutellus|INV12154|Spain|BOLD:AAF7051
Thyreus orbatus|INV12011|Spain|BOLD:AAI0456
Thyreus orbatus|INV12010|Spain|BOLD:AAI0456
Thyreus orbatus|INV12006|Spain|BOLD:AAI0456
Thyreus picaron|INV12461|Spain|BOLD:AED2570
Thyreus ramosus|INV12009|Portugal|BOLD:AAK1852
Thyreus ramosus|INV12008|Spain|BOLD:AAK1852
Thyreus histrionicus|INV12458|Spain|BOLD:AAO3396
Thyreus histrionicus|INV12460|Spain|BOLD:AAO3396
Thyreus histrionicus|INV12457|Spain|BOLD:AAO3396
Thyreus truncatus|INV12459|Spain|BOLD:AES2873
Thyreus hirtus|INV12005|Spain|BOLD:AEO3985
Thyreus hirtus|INV12007|Spain|BOLD:AEO3985
Thyreus hirtus|INV12004|Spain|BOLD:AEO3985
Melecta albifrons|INV12003|Spain|BOLD:ACR2895
Melecta albifrons|INV12002|Spain|BOLD:ACR2895
Melecta sp.|INV12001|Spain|BOLD:AEO2855
Xylocopa iris uclesiensis|INV13231|Portugal|BOLD:AEO4657
Xylocopa iris|INV12172|Spain|BOLD:AEO4657
Xylocopa cantabrita|INV13040|Portugal|BOLD:AEO2264
Xylocopa cantabrita|INV12173|Spain|BOLD:AEO2264
Xylocopa violacea|INV13232|Portugal|BOLD:AAJ9209
Xylocopa violacea|INV12174|Spain|BOLD:AAJ9209
Xylocopa valga|INV12171|Spain|BOLD:AEN0425
Ammobates punctatus|INV12030|Portugal|BOLD:AAC7051
Pasites maculatus|INV12027|Spain|BOLD:AAL3976

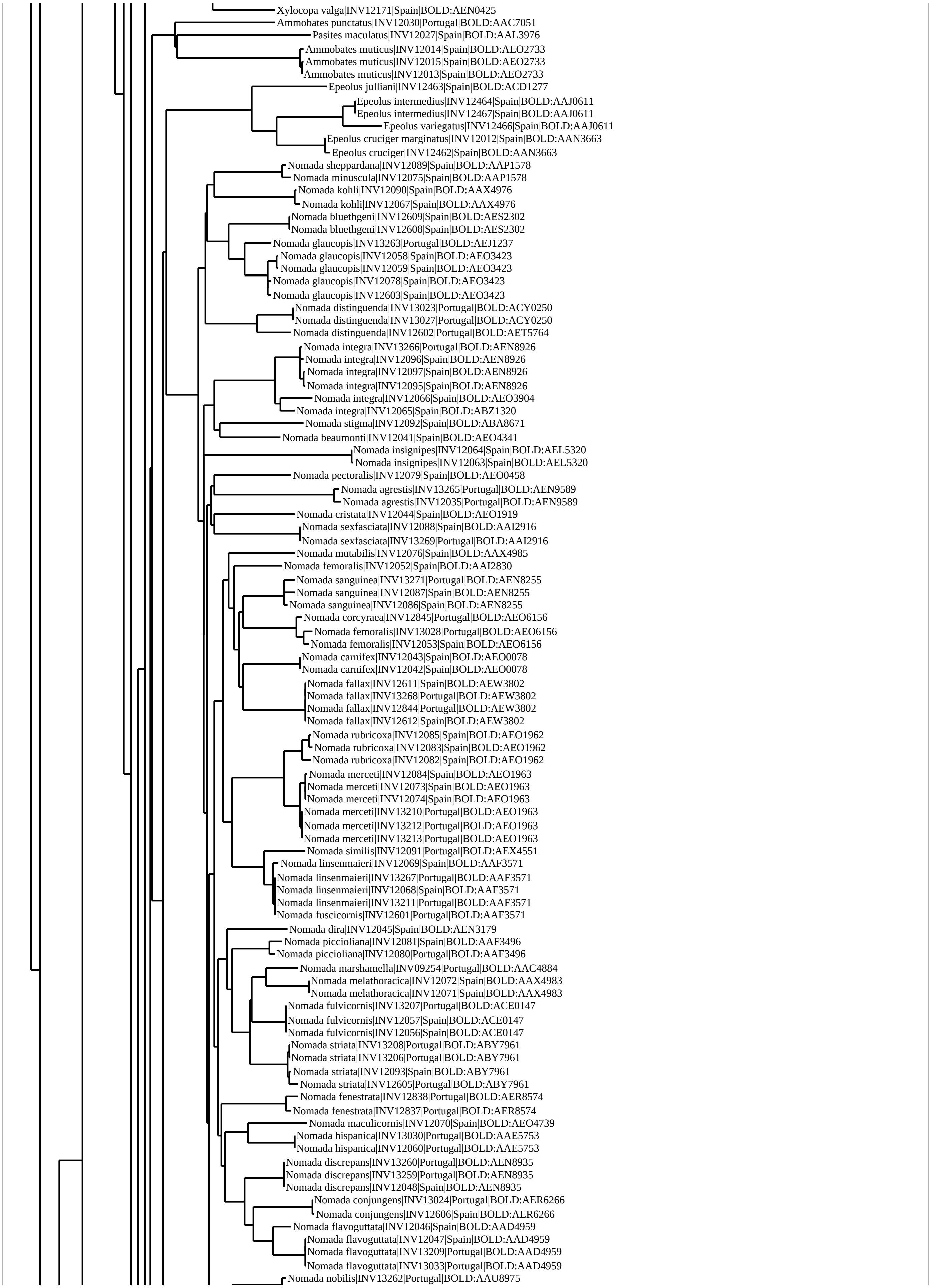
Xylocopa valga|INV12171|Spain|BOLD:AEN0425
Ammobates punctatus|INV12030|Portugal|BOLD:AAC7051
Pasites maculatus|INV12027|Spain|BOLD:AAL3976
Ammobates muticus|INV12014|Spain|BOLD:AEO2733
Ammobates muticus|INV12015|Spain|BOLD:AEO2733
Ammobates muticus|INV12013|Spain|BOLD:AEO2733
Epeolus julliani|INV12463|Spain|BOLD:ACD1277
Epeolus intermedius|INV12464|Spain|BOLD:AAJ0611
Epeolus intermedius|INV12467|Spain|BOLD:AAJ0611
Epeolus variegatus|INV12466|Spain|BOLD:AAJ0611
Epeolus cruciger marginatus|INV12012|Spain|BOLD:AAN3663
Epeolus cruciger|INV12462|Spain|BOLD:AAN3663
Nomada sheppardana|INV12089|Spain|BOLD:AAP1578
Nomada minuscula|INV12075|Spain|BOLD:AAP1578
Nomada kohli|INV12090|Spain|BOLD:AAX4976
Nomada kohli|INV12067|Spain|BOLD:AAX4976
Nomada bluethgeni|INV12609|Spain|BOLD:AES2302
Nomada bluethgeni|INV12608|Spain|BOLD:AES2302
Nomada glaucopis|INV13263|Portugal|BOLD:AEJ1237
Nomada glaucopis|INV12058|Spain|BOLD:AEO3423
Nomada glaucopis|INV12059|Spain|BOLD:AEO3423
Nomada glaucopis|INV12078|Spain|BOLD:AEO3423
Nomada glaucopis|INV12603|Spain|BOLD:AEO3423
Nomada distinguenda|INV13023|Portugal|BOLD:ACY0250
Nomada distinguenda|INV13027|Portugal|BOLD:ACY0250
Nomada distinguenda|INV12602|Portugal|BOLD:AET5764
Nomada integra|INV13266|Portugal|BOLD:AEN8926
Nomada integra|INV12096|Spain|BOLD:AEN8926
Nomada integra|INV12097|Spain|BOLD:AEN8926
Nomada integra|INV12095|Spain|BOLD:AEN8926
Nomada integra|INV12066|Spain|BOLD:AEO3904
Nomada integra|INV12065|Spain|BOLD:ABZ1320
Nomada stigma|INV12092|Spain|BOLD:ABA8671
Nomada beaumonti|INV12041|Spain|BOLD:AEO4341
Nomada insignipes|INV12064|Spain|BOLD:AEL5320
Nomada insignipes|INV12063|Spain|BOLD:AEL5320
Nomada pectoralis|INV12079|Spain|BOLD:AEO0458
Nomada agrestis|INV13265|Portugal|BOLD:AEN9589
Nomada agrestis|INV12035|Portugal|BOLD:AEN9589
Nomada cristata|INV12044|Spain|BOLD:AEO1919
Nomada sexfasciata|INV12088|Spain|BOLD:AAI2916
Nomada sexfasciata|INV13269|Portugal|BOLD:AAI2916
Nomada mutabilis|INV12076|Spain|BOLD:AAX4985
Nomada femoralis|INV12052|Spain|BOLD:AAI2830
Nomada sanguinea|INV13271|Portugal|BOLD:AEN8255
Nomada sanguinea|INV12087|Spain|BOLD:AEN8255
Nomada sanguinea|INV12086|Spain|BOLD:AEN8255
Nomada corcyraea|INV12845|Portugal|BOLD:AEO6156
Nomada femoralis|INV13028|Portugal|BOLD:AEO6156
Nomada femoralis|INV12053|Spain|BOLD:AEO6156
Nomada carnifex|INV12043|Spain|BOLD:AEO0078
Nomada carnifex|INV12042|Spain|BOLD:AEO0078
Nomada fallax|INV12611|Spain|BOLD:AEW3802
Nomada fallax|INV13268|Portugal|BOLD:AEW3802
Nomada fallax|INV12844|Portugal|BOLD:AEW3802
Nomada fallax|INV12612|Spain|BOLD:AEW3802
Nomada rubricoxa|INV12085|Spain|BOLD:AEO1962
Nomada rubricoxa|INV12083|Spain|BOLD:AEO1962
Nomada rubricoxa|INV12082|Spain|BOLD:AEO1962
Nomada merceti|INV12084|Spain|BOLD:AEO1963
Nomada merceti|INV12073|Spain|BOLD:AEO1963
Nomada merceti|INV12074|Spain|BOLD:AEO1963
Nomada merceti|INV13210|Portugal|BOLD:AEO1963
Nomada merceti|INV13212|Portugal|BOLD:AEO1963
Nomada merceti|INV13213|Portugal|BOLD:AEO1963
Nomada similis|INV12091|Portugal|BOLD:AEX4551
Nomada linsenmaieri|INV12069|Spain|BOLD:AAF3571
Nomada linsenmaieri|INV13267|Portugal|BOLD:AAF3571
Nomada linsenmaieri|INV12068|Spain|BOLD:AAF3571
Nomada linsenmaieri|INV13211|Portugal|BOLD:AAF3571
Nomada fuscicornis|INV12601|Portugal|BOLD:AAF3571
Nomada dira|INV12045|Spain|BOLD:AEN3179
Nomada piccioliana|INV12081|Spain|BOLD:AAF3496
Nomada piccioliana|INV12080|Portugal|BOLD:AAF3496
Nomada marshamella|INV09254|Portugal|BOLD:AAC4884
Nomada melathoracica|INV12072|Spain|BOLD:AAX4983
Nomada melathoracica|INV12071|Spain|BOLD:AAX4983
Nomada fulvicornis|INV13207|Portugal|BOLD:ACE0147
Nomada fulvicornis|INV12057|Spain|BOLD:ACE0147
Nomada fulvicornis|INV12056|Spain|BOLD:ACE0147
Nomada striata|INV13208|Portugal|BOLD:ABY7961
Nomada striata|INV13206|Portugal|BOLD:ABY7961
Nomada striata|INV12093|Spain|BOLD:ABY7961
Nomada striata|INV12605|Portugal|BOLD:ABY7961
Nomada fenestrata|INV12838|Portugal|BOLD:AER8574
Nomada fenestrata|INV12837|Portugal|BOLD:AER8574
Nomada maculicornis|INV12070|Spain|BOLD:AEO4739
Nomada hispanica|INV13030|Portugal|BOLD:AAE5753
Nomada hispanica|INV12060|Portugal|BOLD:AAE5753
Nomada discrepans|INV13260|Portugal|BOLD:AEN8935
Nomada discrepans|INV13259|Portugal|BOLD:AEN8935
Nomada discrepans|INV12048|Spain|BOLD:AEN8935
Nomada conjungens|INV13024|Portugal|BOLD:AER6266
Nomada conjungens|INV12606|Spain|BOLD:AER6266
Nomada flavoguttata|INV12046|Spain|BOLD:AAD4959
Nomada flavoguttata|INV12047|Spain|BOLD:AAD4959
Nomada flavoguttata|INV13209|Portugal|BOLD:AAD4959
Nomada flavoguttata|INV13033|Portugal|BOLD:AAD4959
Nomada nobilis|INV13262|Portugal|BOLD:AAU8975

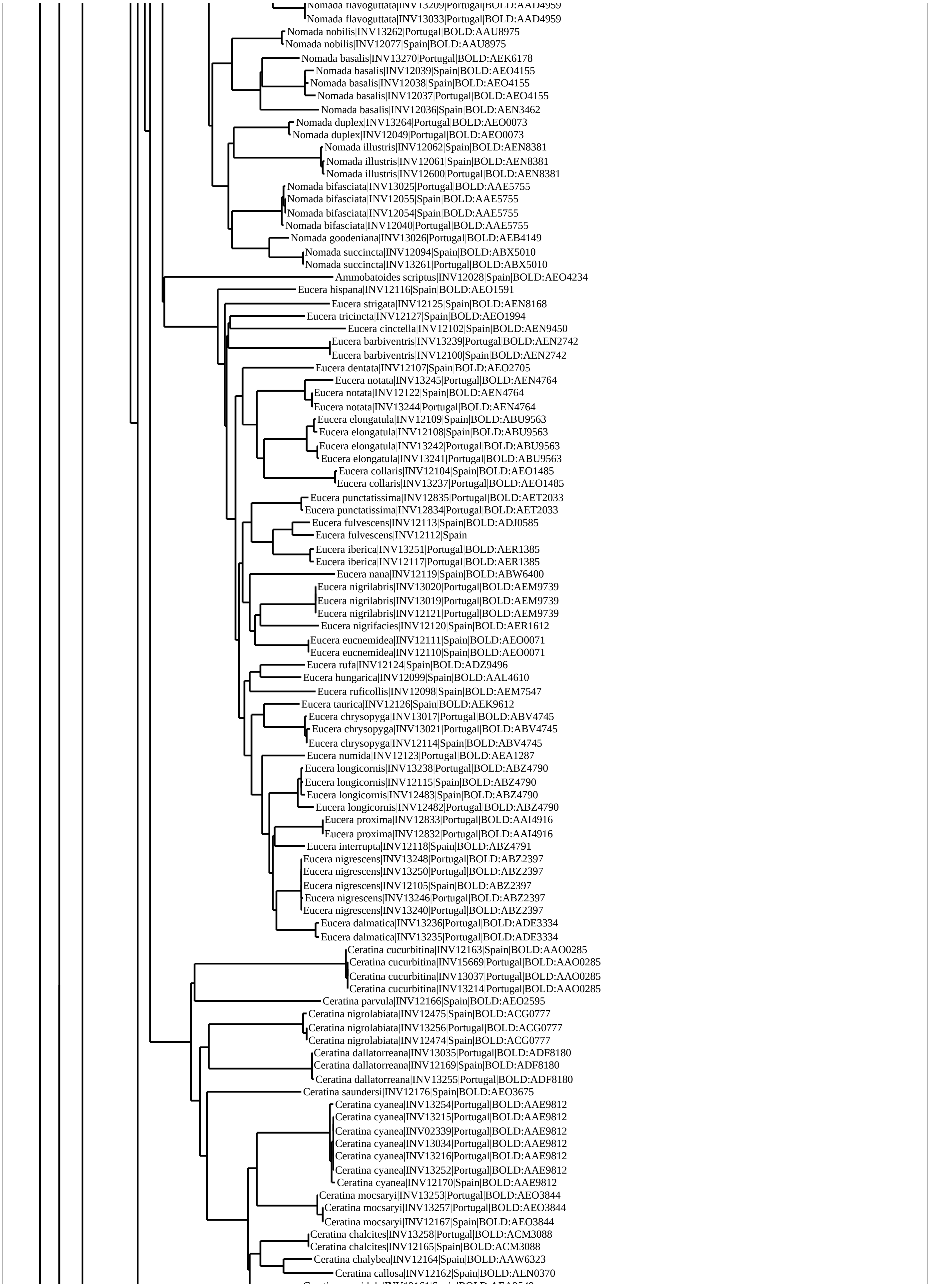
Nomada flavoguttata|INV13209|Portugal|BOLD:AAD4959
Nomada flavoguttata|INV13033|Portugal|BOLD:AAD4959
Nomada nobilis|INV13262|Portugal|BOLD:AAU8975
Nomada nobilis|INV12077|Spain|BOLD:AAU8975
Nomada basalis|INV13270|Portugal|BOLD:AEK6178
Nomada basalis|INV12039|Spain|BOLD:AEO4155
Nomada basalis|INV12038|Spain|BOLD:AEO4155
Nomada basalis|INV12037|Portugal|BOLD:AEO4155
Nomada basalis|INV12036|Spain|BOLD:AEN3462
Nomada duplex|INV13264|Portugal|BOLD:AEO0073
Nomada duplex|INV12049|Portugal|BOLD:AEO0073
Nomada illustris|INV12062|Spain|BOLD:AEN8381
Nomada illustris|INV12061|Spain|BOLD:AEN8381
Nomada illustris|INV12600|Portugal|BOLD:AEN8381
Nomada bifasciata|INV13025|Portugal|BOLD:AAE5755
Nomada bifasciata|INV12055|Spain|BOLD:AAE5755
Nomada bifasciata|INV12054|Spain|BOLD:AAE5755
Nomada bifasciata|INV12040|Portugal|BOLD:AAE5755
Nomada goodeniana|INV13026|Portugal|BOLD:AEB4149
Nomada succincta|INV12094|Spain|BOLD:ABX5010
Nomada succincta|INV13261|Portugal|BOLD:ABX5010
Ammobatoides scriptus|INV12028|Spain|BOLD:AEO4234
Eucera hispana|INV12116|Spain|BOLD:AEO1591
Eucera strigata|INV12125|Spain|BOLD:AEN8168
Eucera tricincta|INV12127|Spain|BOLD:AEO1994
Eucera cinctella|INV12102|Spain|BOLD:AEN9450
Eucera barbiventris|INV13239|Portugal|BOLD:AEN2742
Eucera barbiventris|INV12100|Spain|BOLD:AEN2742
Eucera dentata|INV12107|Spain|BOLD:AEO2705
Eucera notata|INV13245|Portugal|BOLD:AEN4764
Eucera notata|INV12122|Spain|BOLD:AEN4764
Eucera notata|INV13244|Portugal|BOLD:AEN4764
Eucera elongatula|INV12109|Spain|BOLD:ABU9563
Eucera elongatula|INV12108|Spain|BOLD:ABU9563
Eucera elongatula|INV13242|Portugal|BOLD:ABU9563
Eucera elongatula|INV13241|Portugal|BOLD:ABU9563
Eucera collaris|INV12104|Spain|BOLD:AEO1485
Eucera collaris|INV13237|Portugal|BOLD:AEO1485
Eucera punctatissima|INV12835|Portugal|BOLD:AET2033
Eucera punctatissima|INV12834|Portugal|BOLD:AET2033
Eucera fulvescens|INV12113|Spain|BOLD:ADJ0585
Eucera fulvescens|INV12112|Spain
Eucera iberica|INV13251|Portugal|BOLD:AER1385
Eucera iberica|INV12117|Portugal|BOLD:AER1385
Eucera nana|INV12119|Spain|BOLD:ABW6400
Eucera nigrilabris|INV13020|Portugal|BOLD:AEM9739
Eucera nigrilabris|INV13019|Portugal|BOLD:AEM9739
Eucera nigrilabris|INV12121|Portugal|BOLD:AEM9739
Eucera nigrifacies|INV12120|Spain|BOLD:AER1612
Eucera eucnemidea|INV12111|Spain|BOLD:AEO0071
Eucera eucnemidea|INV12110|Spain|BOLD:AEO0071
Eucera rufa|INV12124|Spain|BOLD:ADZ9496
Eucera hungarica|INV12099|Spain|BOLD:AAL4610
Eucera ruficollis|INV12098|Spain|BOLD:AEM7547
Eucera taurica|INV12126|Spain|BOLD:AEK9612
Eucera chrysopyga|INV13017|Portugal|BOLD:ABV4745
Eucera chrysopyga|INV13021|Portugal|BOLD:ABV4745
Eucera chrysopyga|INV12114|Spain|BOLD:ABV4745
Eucera numida|INV12123|Portugal|BOLD:AEA1287
Eucera longicornis|INV13238|Portugal|BOLD:ABZ4790
Eucera longicornis|INV12115|Spain|BOLD:ABZ4790
Eucera longicornis|INV12483|Spain|BOLD:ABZ4790
Eucera longicornis|INV12482|Portugal|BOLD:ABZ4790
Eucera proxima|INV12833|Portugal|BOLD:AAI4916
Eucera proxima|INV12832|Portugal|BOLD:AAI4916
Eucera interrupta|INV12118|Spain|BOLD:ABZ4791
Eucera nigrescens|INV13248|Portugal|BOLD:ABZ2397
Eucera nigrescens|INV13250|Portugal|BOLD:ABZ2397
Eucera nigrescens|INV12105|Spain|BOLD:ABZ2397
Eucera nigrescens|INV13246|Portugal|BOLD:ABZ2397
Eucera nigrescens|INV13240|Portugal|BOLD:ABZ2397
Eucera dalmatica|INV13236|Portugal|BOLD:ADE3334
Eucera dalmatica|INV13235|Portugal|BOLD:ADE3334
Ceratina cucurbitina|INV12163|Spain|BOLD:AAO0285
Ceratina cucurbitina|INV15669|Portugal|BOLD:AAO0285
Ceratina cucurbitina|INV13037|Portugal|BOLD:AAO0285
Ceratina cucurbitina|INV13214|Portugal|BOLD:AAO0285
Ceratina parvula|INV12166|Spain|BOLD:AEO2595
Ceratina nigrolabiata|INV12475|Spain|BOLD:ACG0777
Ceratina nigrolabiata|INV13256|Portugal|BOLD:ACG0777
Ceratina nigrolabiata|INV12474|Spain|BOLD:ACG0777
Ceratina dallatorreana|INV13035|Portugal|BOLD:ADF8180
Ceratina dallatorreana|INV12169|Spain|BOLD:ADF8180
Ceratina dallatorreana|INV13255|Portugal|BOLD:ADF8180
Ceratina saundersi|INV12176|Spain|BOLD:AEO3675
Ceratina cyanea|INV13254|Portugal|BOLD:AAE9812
Ceratina cyanea|INV13215|Portugal|BOLD:AAE9812
Ceratina cyanea|INV02339|Portugal|BOLD:AAE9812
Ceratina cyanea|INV13034|Portugal|BOLD:AAE9812
Ceratina cyanea|INV13216|Portugal|BOLD:AAE9812
Ceratina cyanea|INV13252|Portugal|BOLD:AAE9812
Ceratina cyanea|INV12170|Spain|BOLD:AAE9812
Ceratina mocsaryi|INV13253|Portugal|BOLD:AEO3844
Ceratina mocsaryi|INV13257|Portugal|BOLD:AEO3844
Ceratina mocsaryi|INV12167|Spain|BOLD:AEO3844
Ceratina chalcites|INV13258|Portugal|BOLD:ACM3088
Ceratina chalcites|INV12165|Spain|BOLD:ACM3088
Ceratina chalybea|INV12164|Spain|BOLD:AAW6323
Ceratina callosa|INV12162|Spain|BOLD:AEN0370

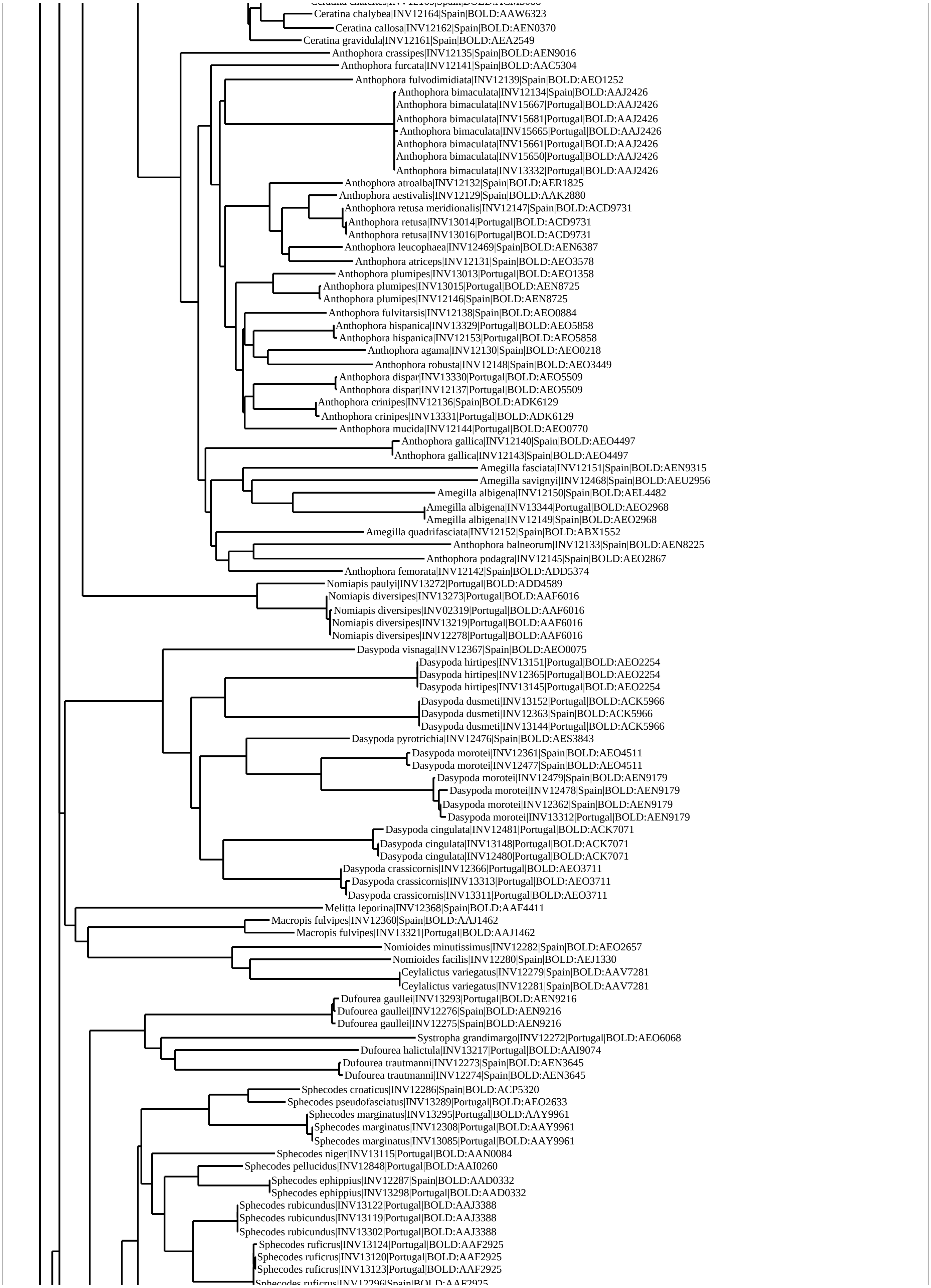
Ceratina chalybea|INV12164|Spain|BOLD:AAW6323
Ceratina callosa|INV12162|Spain|BOLD:AEN0370
Ceratina gravidula|INV12161|Spain|BOLD:AEA2549
Anthophora crassipes|INV12135|Spain|BOLD:AEN9016
Anthophora furcata|INV12141|Spain|BOLD:AAC5304
Anthophora fulvodimidiata|INV12139|Spain|BOLD:AEO1252
Anthophora bimaculata|INV12134|Spain|BOLD:AAJ2426
Anthophora bimaculata|INV15667|Portugal|BOLD:AAJ2426
Anthophora bimaculata|INV15681|Portugal|BOLD:AAJ2426
Anthophora bimaculata|INV15665|Portugal|BOLD:AAJ2426
Anthophora bimaculata|INV15661|Portugal|BOLD:AAJ2426
Anthophora bimaculata|INV15650|Portugal|BOLD:AAJ2426
Anthophora bimaculata|INV13332|Portugal|BOLD:AAJ2426
Anthophora atroalba|INV12132|Spain|BOLD:AER1825
Anthophora aestivalis|INV12129|Spain|BOLD:AAK2880
Anthophora retusa meridionalis|INV12147|Spain|BOLD:ACD9731
Anthophora retusa|INV13014|Portugal|BOLD:ACD9731
Anthophora retusa|INV13016|Portugal|BOLD:ACD9731
Anthophora leucophaea|INV12469|Spain|BOLD:AEN6387
Anthophora atriceps|INV12131|Spain|BOLD:AEO3578
Anthophora plumipes|INV13013|Portugal|BOLD:AEO1358
Anthophora plumipes|INV13015|Portugal|BOLD:AEN8725
Anthophora plumipes|INV12146|Spain|BOLD:AEN8725
Anthophora fulvitarsis|INV12138|Spain|BOLD:AEO0884
Anthophora hispanica|INV13329|Portugal|BOLD:AEO5858
Anthophora hispanica|INV12153|Portugal|BOLD:AEO5858
Anthophora agama|INV12130|Spain|BOLD:AEO0218
Anthophora robusta|INV12148|Spain|BOLD:AEO3449
Anthophora dispar|INV13330|Portugal|BOLD:AEO5509
Anthophora dispar|INV12137|Portugal|BOLD:AEO5509
Anthophora crinipes|INV12136|Spain|BOLD:ADK6129
Anthophora crinipes|INV13331|Portugal|BOLD:ADK6129
Anthophora mucida|INV12144|Portugal|BOLD:AEO0770
Anthophora gallica|INV12140|Spain|BOLD:AEO4497
Anthophora gallica|INV12143|Spain|BOLD:AEO4497
Amegilla fasciata|INV12151|Spain|BOLD:AEN9315
Amegilla savignyi|INV12468|Spain|BOLD:AEU2956
Amegilla albigena|INV12150|Spain|BOLD:AEL4482
Amegilla albigena|INV13344|Portugal|BOLD:AEO2968
Amegilla albigena|INV12149|Spain|BOLD:AEO2968
Amegilla quadrifasciata|INV12152|Spain|BOLD:ABX1552
Anthophora balneorum|INV12133|Spain|BOLD:AEN8225
Anthophora podagra|INV12145|Spain|BOLD:AEO2867
Anthophora femorata|INV12142|Spain|BOLD:ADD5374
Nomiapis paulyi|INV13272|Portugal|BOLD:ADD4589
Nomiapis diversipes|INV13273|Portugal|BOLD:AAF6016
Nomiapis diversipes|INV02319|Portugal|BOLD:AAF6016
Nomiapis diversipes|INV13219|Portugal|BOLD:AAF6016
Nomiapis diversipes|INV12278|Portugal|BOLD:AAF6016
Dasypoda visnaga|INV12367|Spain|BOLD:AEO0075
Dasypoda hirtipes|INV13151|Portugal|BOLD:AEO2254
Dasypoda hirtipes|INV12365|Portugal|BOLD:AEO2254
Dasypoda hirtipes|INV13145|Portugal|BOLD:AEO2254
Dasypoda dusmeti|INV13152|Portugal|BOLD:ACK5966
Dasypoda dusmeti|INV12363|Spain|BOLD:ACK5966
Dasypoda dusmeti|INV13144|Portugal|BOLD:ACK5966
Dasypoda pyrotrichia|INV12476|Spain|BOLD:AES3843
Dasypoda morotei|INV12361|Spain|BOLD:AEO4511
Dasypoda morotei|INV12477|Spain|BOLD:AEO4511
Dasypoda morotei|INV12479|Spain|BOLD:AEN9179
Dasypoda morotei|INV12478|Spain|BOLD:AEN9179
Dasypoda morotei|INV12362|Spain|BOLD:AEN9179
Dasypoda morotei|INV13312|Portugal|BOLD:AEN9179
Dasypoda cingulata|INV12481|Portugal|BOLD:ACK7071
Dasypoda cingulata|INV13148|Portugal|BOLD:ACK7071
Dasypoda cingulata|INV12480|Portugal|BOLD:ACK7071
Dasypoda crassicornis|INV12366|Portugal|BOLD:AEO3711
Dasypoda crassicornis|INV13313|Portugal|BOLD:AEO3711
Dasypoda crassicornis|INV13311|Portugal|BOLD:AEO3711
Melitta leporina|INV12368|Spain|BOLD:AAF4411
Macropis fulvipes|INV12360|Spain|BOLD:AAJ1462
Macropis fulvipes|INV13321|Portugal|BOLD:AAJ1462
Nomioides minutissimus|INV12282|Spain|BOLD:AEO2657
Nomioides facilis|INV12280|Spain|BOLD:AEJ1330
Ceylalictus variegatus|INV12279|Spain|BOLD:AAV7281
Ceylalictus variegatus|INV12281|Spain|BOLD:AAV7281
Dufourea gaullei|INV13293|Portugal|BOLD:AEN9216
Dufourea gaullei|INV12276|Spain|BOLD:AEN9216
Dufourea gaullei|INV12275|Spain|BOLD:AEN9216
Systropha grandimargo|INV12272|Portugal|BOLD:AEO6068
Dufourea halictula|INV13217|Portugal|BOLD:AAI9074
Dufourea trautmanni|INV12273|Spain|BOLD:AEN3645
Dufourea trautmanni|INV12274|Spain|BOLD:AEN3645
Sphecodes croaticus|INV12286|Spain|BOLD:ACP5320
Sphecodes pseudofasciatus|INV13289|Portugal|BOLD:AEO2633
Sphecodes marginatus|INV13295|Portugal|BOLD:AAY9961
Sphecodes marginatus|INV12308|Portugal|BOLD:AAY9961
Sphecodes marginatus|INV13085|Portugal|BOLD:AAY9961
Sphecodes niger|INV13115|Portugal|BOLD:AAN0084
Sphecodes pellucidus|INV12848|Portugal|BOLD:AAI0260
Sphecodes ephippius|INV12287|Spain|BOLD:AAD0332
Sphecodes ephippius|INV13298|Portugal|BOLD:AAD0332
Sphecodes rubicundus|INV13122|Portugal|BOLD:AAJ3388
Sphecodes rubicundus|INV13119|Portugal|BOLD:AAJ3388
Sphecodes rubicundus|INV13302|Portugal|BOLD:AAJ3388
Sphecodes ruficrus|INV13124|Portugal|BOLD:AAF2925
Sphecodes ruficrus|INV13120|Portugal|BOLD:AAF2925
Sphecodes ruficrus|INV13123|Portugal|BOLD:AAF2925
Sphecodes ruficrus|INV12296|Spain|BOLD:AAF2925

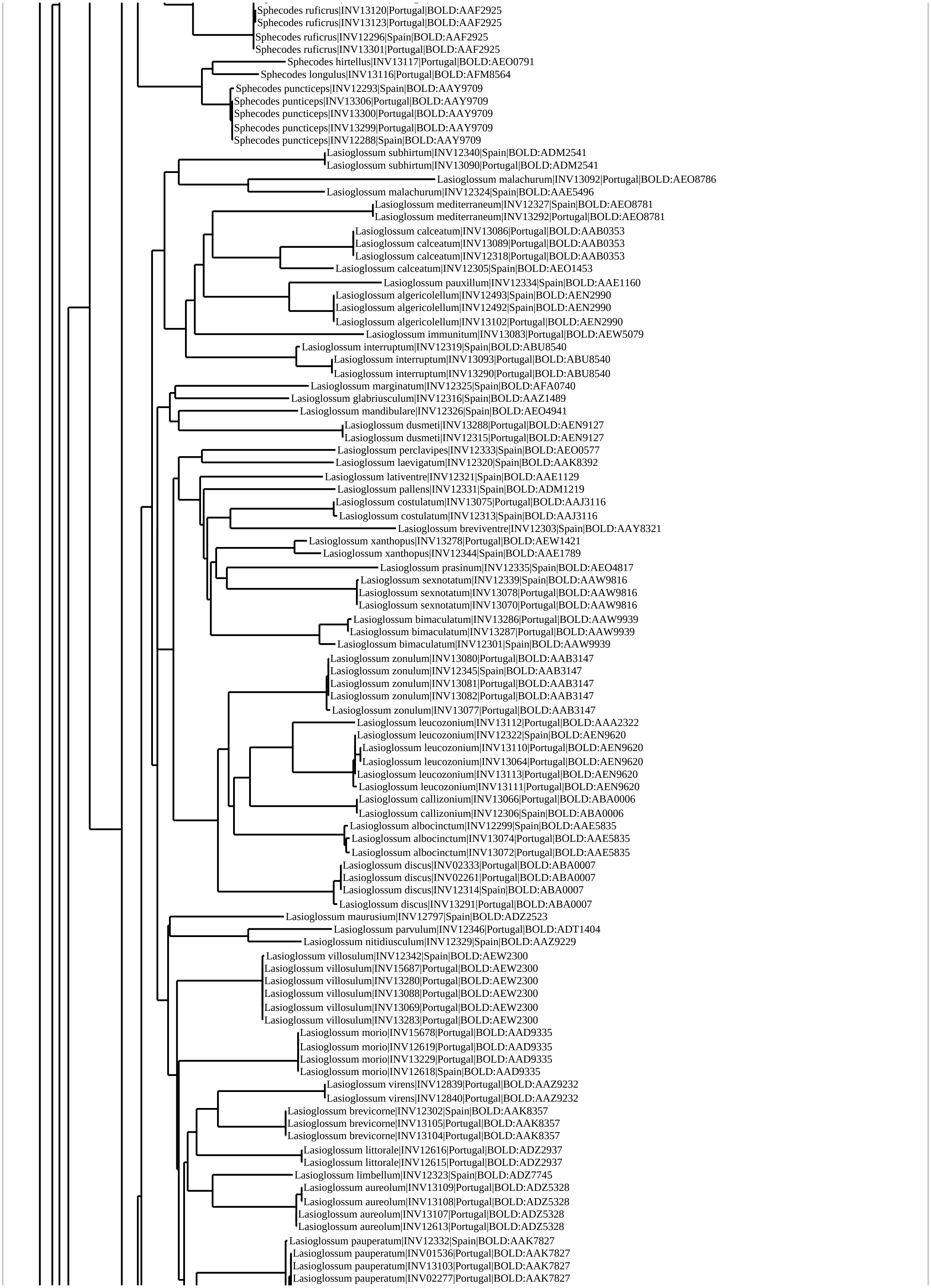
Sphecodes ruficrus|INV13120|Portugal|BOLD:AAF2925
Sphecodes ruficrus|INV13123|Portugal|BOLD:AAF2925
Sphecodes ruficrus|INV12296|Spain|BOLD:AAF2925
Sphecodes ruficrus|INV13301|Portugal|BOLD:AAF2925
Sphecodes hirtellus|INV13117|Portugal|BOLD:AEO0791
Sphecodes longulus|INV13116|Portugal|BOLD:AFM8564
Sphecodes puncticeps|INV12293|Spain|BOLD:AAY9709
Sphecodes punticeps|INV13306|Portugal|BOLD:AAY9709
Sphecodes puncticeps|INV13300|Portugal|BOLD:AAY9709
Sphecodes puncticeps|INV13299|Portugal|BOLD:AAY9709
Sphecodes puncticeps|INV12288|Spain|BOLD:AAY9709
Lasioglossum subhirtum|INV12340|Spain|BOLD:ADM2541
Lasioglossum subhirtum|INV13090|Spain|BOLD:ADM2541
Lasioglossum malachurum|INV13092|Portugal|BOLD:AEO8786
Lasioglossum malachurum|INV12324|Spain|BOLD:AAE5496
Lasioglossum mediterraneum|INV12327|Spain|BOLD:AEO8781
Lasioglossum mediterraneum|INV13292|Portugal|BOLD:AEO8781
Lasioglossum calceatum|INV13086|Portugal|BOLD:AAB0353
Lasioglossum calceatum|INV13089|Portugal|BOLD:AAB0353
Lasioglossum calceatum|INV12318|Portugal|BOLD:AAB0353
Lasioglossum calceatum|INV12305|Spain|BOLD:AEO1453
Lasioglossum pauxillum|INV12334|Spain|BOLD:AAE1160
Lasioglossum algericolellum|INV12493|Spain|BOLD:AEN2990
Lasioglossum algericolellum|INV12492|Spain|BOLD:AEN2990
Lasioglossum algericolellum|INV13102|Portugal|BOLD:AEN2990
Lasioglossum immunitum|INV13083|Portugal|BOLD:AEW5079
Lasioglossum interruptum|INV12319|Spain|BOLD:ABU8540
Lasioglossum interruptum|INV13093|Portugal|BOLD:ABU8540
Lasioglossum interruptum|INV13290|Portugal|BOLD:ABU8540
Lasioglossum marginatum|INV12325|Spain|BOLD:AFA0740
Lasioglossum glabriusculum|INV12316|Spain|BOLD:AAZ1489
Lasioglossum mandibulare|INV12326|Spain|BOLD:AEO4941
Lasioglossum dusmeti|INV13288|Portugal|BOLD:AEN9127
Lasioglossum dusmeti|INV12315|Portugal|BOLD:AEN9127
Lasioglossum perclavipes|INV12333|Spain|BOLD:AEO0577
Lasioglossum laevigatum|INV12320|Spain|BOLD:AAK8392
Lasioglossum lativentre|INV12321|Spain|BOLD:AAE1129
Lasioglossum pallens|INV12331|Spain|BOLD:ADM1219
Lasioglossum costulatum|INV13075|Portugal|BOLD:AAJ3116
Lasioglossum costulatum|INV12313|Spain|BOLD:AAJ3116
Lasioglossum breviventre|INV12303|Spain|BOLD:AAY8321
Lasioglossum xanthopus|INV13278|Portugal|BOLD:AEW1421
Lasioglossum xanthopus|INV12344|Spain|BOLD:AAE1789
Lasioglossum prasinum|INV12335|Spain|BOLD:AEO4817
Lasioglossum sexnotatum|INV12339|Spain|BOLD:AAW9816
Lasioglossum sexnotatum|INV13078|Portugal|BOLD:AAW9816
Lasioglossum sexnotatum|INV13070|Portugal|BOLD:AAW9816
Lasioglossum bimaculatum|INV13286|Portugal|BOLD:AAW9939
Lasioglossum bimaculatum|INV13287|Portugal|BOLD:AAW9939
Lasioglossum bimaculatum|INV12301|Spain|BOLD:AAW9939
Lasioglossum zonulum|INV13080|Portugal|BOLD:AAB3147
Lasioglossum zonulum|INV12345|Spain|BOLD:AAB3147
Lasioglossum zonulum|INV13081|Portugal|BOLD:AAB3147
Lasioglossum zonulum|INV13082|Portugal|BOLD:AAB3147
Lasioglossum zonulum|INV13077|Portugal|BOLD:AAB3147
Lasioglossum leucozonium|INV13112|Portugal|BOLD:AAA2322
Lasioglossum leucozonium|INV12322|Spain|BOLD:AEN9620
Lasioglossum leucozonium|INV13110|Portugal|BOLD:AEN9620
Lasioglossum leucozonium|INV13064|Portugal|BOLD:AEN9620
Lasioglossum leucozonium|INV13113|Portugal|BOLD:AEN9620
Lasioglossum leucozonium|INV13111|Portugal|BOLD:AEN9620
Lasioglossum callizonium|INV13066|Portugal|BOLD:ABA0006
Lasioglossum callizonium|INV12306|Spain|BOLD:ABA0006
Lasioglossum albocinctum|INV12299|Spain|BOLD:AAE5835
Lasioglossum albocinctum|INV13074|Portugal|BOLD:AAE5835
Lasioglossum albocinctum|INV13072|Portugal|BOLD:AAE5835
Lasioglossum discus|INV02333|Portugal|BOLD:ABA0007
Lasioglossum discus|INV02261|Portugal|BOLD:ABA0007
Lasioglossum discus|INV12314|Spain|BOLD:ABA0007
Lasioglossum discus|INV13291|Portugal|BOLD:ABA0007
Lasioglossum maurusium|INV12797|Spain|BOLD:ADZ2523
Lasioglossum parvulum|INV12346|Portugal|BOLD:ADT1404
Lasioglossum nitidiusculum|INV12329|Spain|BOLD:AAZ9229
Lasioglossum villosulum|INV12342|Spain|BOLD:AEW2300
Lasioglossum villosulum|INV15687|Portugal|BOLD:AEW2300
Lasioglossum villosulum|INV13280|Portugal|BOLD:AEW2300
Lasioglossum villosulum|INV13088|Portugal|BOLD:AEW2300
Lasioglossum villosulum|INV13069|Portugal|BOLD:AEW2300
Lasioglossum villosulum|INV13283|Portugal|BOLD:AEW2300
Lasioglossum morio|INV15678|Portugal|BOLD:AAD9335
Lasioglossum morio|INV12619|Portugal|BOLD:AAD9335
Lasioglossum morio|INV13229|Portugal|BOLD:AAD9335
Lasioglossum morio|INV12618|Spain|BOLD:AAD9335
Lasioglossum virens|INV12839|Portugal|BOLD:AAZ9232
Lasioglossum virens|INV12840|Portugal|BOLD:AAZ9232
Lasioglossum brevicorne|INV12302|Spain|BOLD:AAK8357
Lasioglossum brevicorne|INV13105|Portugal|BOLD:AAK8357
Lasioglossum brevicorne|INV13104|Portugal|BOLD:AAK8357
Lasioglossum littorale|INV12616|Portugal|BOLD:ADZ2937
Lasioglossum littorale|INV12615|Portugal|BOLD:ADZ2937
Lasioglossum limbellum|INV12323|Spain|BOLD:ADZ7745
Lasioglossum aureolum|INV13109|Portugal|BOLD:ADZ5328
Lasioglossum aureolum|INV13108|Portugal|BOLD:ADZ5328
Lasioglossum aureolum|INV13107|Portugal|BOLD:ADZ5328
Lasioglossum aureolum|INV12613|Portugal|BOLD:ADZ5328
Lasioglossum pauperatum|INV12332|Spain|BOLD:AAK7827
Lasioglossum pauperatum|INV01536|Portugal|BOLD:AAK7827
Lasioglossum pauperatum|INV13103|Portugal|BOLD:AAK7827
Lasioglossum pauperatum|INV02277|Portugal|BOLD:AAK7827

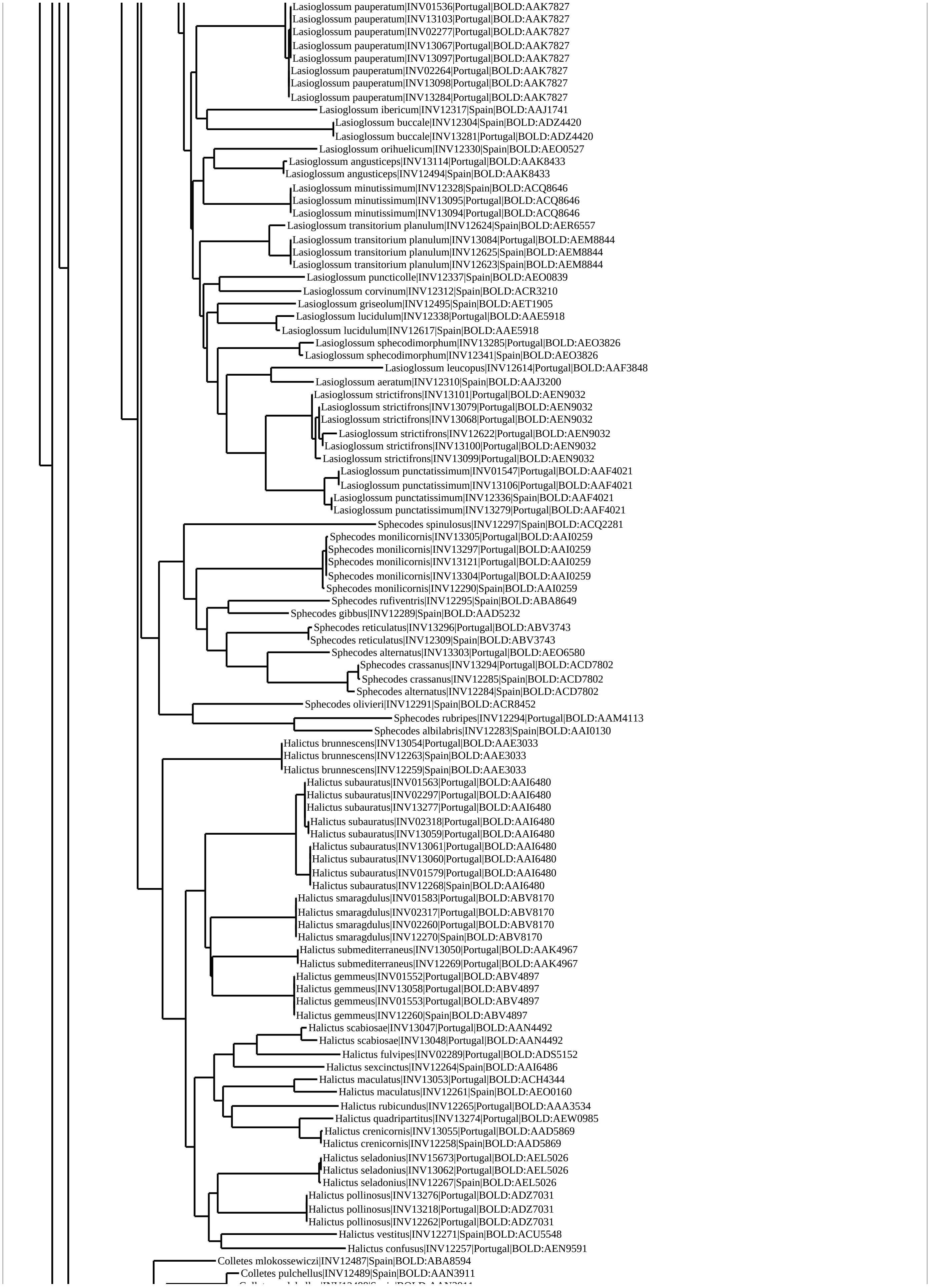
Lasioglossum pauperatum|INV01536|Portugal|BOLD:AAK7827
Lasioglossum pauperatum|INV13103|Portugal|BOLD:AAK7827
Lasioglossum pauperatum|INV02277|Portugal|BOLD:AAK7827
Lasioglossum pauperatum|INV13067|Portugal|BOLD:AAK7827
Lasioglossum pauperatum|INV13097|Portugal|BOLD:AAK7827
Lasioglossum pauperatum|INV02264|Portugal|BOLD:AAK7827
Lasioglossum pauperatum|INV13098|Portugal|BOLD:AAK7827
Lasioglossum pauperatum|INV13284|Portugal|BOLD:AAK7827
Lasioglossum ibericum|INV12317|Spain|BOLD:AAJ1741
Lasioglossum buccale|INV12304|Spain|BOLD:ADZ4420
Lasioglossum buccale|INV13281|Portugal|BOLD:ADZ4420
Lasioglossum orihuelicum|INV12330|Spain|BOLD:AEO0527
Lasioglossum angusticeps|INV13114|Portugal|BOLD:AAK8433
Lasioglossum angusticeps|INV12494|Spain|BOLD:AAK8433
Lasioglossum minutissimum|INV12328|Spain|BOLD:ACQ8646
Lasioglossum minutissimum|INV13095|Portugal|BOLD:ACQ8646
Lasioglossum minutissimum|INV13094|Portugal|BOLD:ACQ8646
Lasioglossum transitorium planulum|INV12624|Spain|BOLD:AER6557
Lasioglossum transitorium planulum|INV13084|Portugal|BOLD:AEM8844
Lasioglossum transitorium planulum|INV12625|Spain|BOLD:AEM8844
Lasioglossum transitorium planulum|INV12623|Spain|BOLD:AEM8844
Lasioglossum puncticolle|INV12337|Spain|BOLD:AEO0839
Lasioglossum corvinum|INV12312|Spain|BOLD:ACR3210
Lasioglossum griseolum|INV12495|Spain|BOLD:AET1905
Lasioglossum lucidulum|INV12338|Portugal|BOLD:AAE5918
Lasioglossum lucidulum|INV12617|Spain|BOLD:AAE5918
Lasioglossum sphecodimorphum|INV13285|Portugal|BOLD:AEO3826
Lasioglossum sphecodimorphum|INV12341|Spain|BOLD:AEO3826
Lasioglossum leucopus|INV12614|Portugal|BOLD:AAF3848
Lasioglossum aeratum|INV12310|Spain|BOLD:AAJ3200
Lasioglossum strictifrons|INV13101|Portugal|BOLD:AEN9032
Lasioglossum strictifrons|INV13079|Portugal|BOLD:AEN9032
Lasioglossum strictifrons|INV13068|Portugal|BOLD:AEN9032
Lasioglossum strictifrons|INV12622|Portugal|BOLD:AEN9032
Lasioglossum strictifrons|INV13100|Portugal|BOLD:AEN9032
Lasioglossum strictifrons|INV13099|Portugal|BOLD:AEN9032
Lasioglossum punctatissimum|INV01547|Portugal|BOLD:AAF4021
Lasioglossum punctatissimum|INV13106|Portugal|BOLD:AAF4021
Lasioglossum punctatissimum|INV12336|Spain|BOLD:AAF4021
Lasioglossum punctatissimum|INV13279|Portugal|BOLD:AAF4021
Sphecodes spinulosus|INV12297|Spain|BOLD:ACQ2281
Sphecodes monilicornis|INV13305|Portugal|BOLD:AAI0259
Sphecodes monilicornis|INV13297|Portugal|BOLD:AAI0259
Sphecodes monilicornis|INV13121|Portugal|BOLD:AAI0259
Sphecodes monilicornis|INV13304|Portugal|BOLD:AAI0259
Sphecodes monilicornis|INV12290|Spain|BOLD:AAI0259
Sphecodes rufiventris|INV12295|Spain|BOLD:ABA8649
Sphecodes gibbus|INV12289|Spain|BOLD:AAD5232
Sphecodes reticulatus|INV13296|Portugal|BOLD:ABV3743
Sphecodes reticulatus|INV12309|Spain|BOLD:ABV3743
Sphecodes alternatus|INV13303|Portugal|BOLD:AEO6580
Sphecodes crassanus|INV13294|Portugal|BOLD:ACD7802
Sphecodes crassanus|INV12285|Spain|BOLD:ACD7802
Sphecodes alternatus|INV12284|Spain|BOLD:ACD7802
Sphecodes olivieri|INV12291|Spain|BOLD:ACR8452
Sphecodes rubripes|INV12294|Portugal|BOLD:AAM4113
Sphecodes albilabris|INV12283|Spain|BOLD:AAI0130
Halictus brunnescens|INV13054|Portugal|BOLD:AAE3033
Halictus brunnescens|INV12263|Spain|BOLD:AAE3033
Halictus brunnescens|INV12259|Spain|BOLD:AAE3033
Halictus subauratus|INV01563|Portugal|BOLD:AAI6480
Halictus subauratus|INV02297|Portugal|BOLD:AAI6480
Halictus subauratus|INV13277|Portugal|BOLD:AAI6480
Halictus subauratus|INV02318|Portugal|BOLD:AAI6480
Halictus subauratus|INV13059|Portugal|BOLD:AAI6480
Halictus subauratus|INV13061|Portugal|BOLD:AAI6480
Halictus subauratus|INV13060|Portugal|BOLD:AAI6480
Halictus subauratus|INV01579|Portugal|BOLD:AAI6480
Halictus subauratus|INV12268|Spain|BOLD:AAI6480
Halictus smaragdulus|INV01583|Portugal|BOLD:ABV8170
Halictus smaragdulus|INV02317|Portugal|BOLD:ABV8170
Halictus smaragdulus|INV02260|Portugal|BOLD:ABV8170
Halictus smaragdulus|INV12270|Spain|BOLD:ABV8170
Halictus submediterraneus|INV13050|Portugal|BOLD:AAK4967
Halictus submediterraneus|INV12269|Portugal|BOLD:AAK4967
Halictus gemmeus|INV01552|Portugal|BOLD:ABV4897
Halictus gemmeus|INV13058|Portugal|BOLD:ABV4897
Halictus gemmeus|INV01553|Portugal|BOLD:ABV4897
Halictus gemmeus|INV12260|Spain|BOLD:ABV4897
Halictus scabiosae|INV13047|Portugal|BOLD:AAN4492
Halictus scabiosae|INV13048|Portugal|BOLD:AAN4492
Halictus fulvipes|INV02289|Portugal|BOLD:ADS5152
Halictus sexcinctus|INV12264|Spain|BOLD:AAI6486
Halictus maculatus|INV13053|Portugal|BOLD:ACH4344
Halictus maculatus|INV12261|Spain|BOLD:AEO0160
Halictus rubicundus|INV12265|Portugal|BOLD:AAA3534
Halictus quadripartitus|INV13274|Portugal|BOLD:AEW0985
Halictus crenicornis|INV13055|Portugal|BOLD:AAD5869
Halictus crenicornis|INV12258|Spain|BOLD:AAD5869
Halictus seladonius|INV15673|Portugal|BOLD:AEL5026
Halictus seladonius|INV13062|Portugal|BOLD:AEL5026
Halictus seladonius|INV12267|Spain|BOLD:AEL5026
Halictus pollinosus|INV13276|Portugal|BOLD:ADZ7031
Halictus pollinosus|INV13218|Portugal|BOLD:ADZ7031
Halictus pollinosus|INV12262|Portugal|BOLD:ADZ7031
Halictus vestitus|INV12271|Spain|BOLD:ACU5548
Halictus confusus|INV12257|Portugal|BOLD:AEN9591
Colletes mlokossewiczi|INV12487|Spain|BOLD:ABA8594
Colletes pulchellus|INV12489|Spain|BOLD:AAN3911

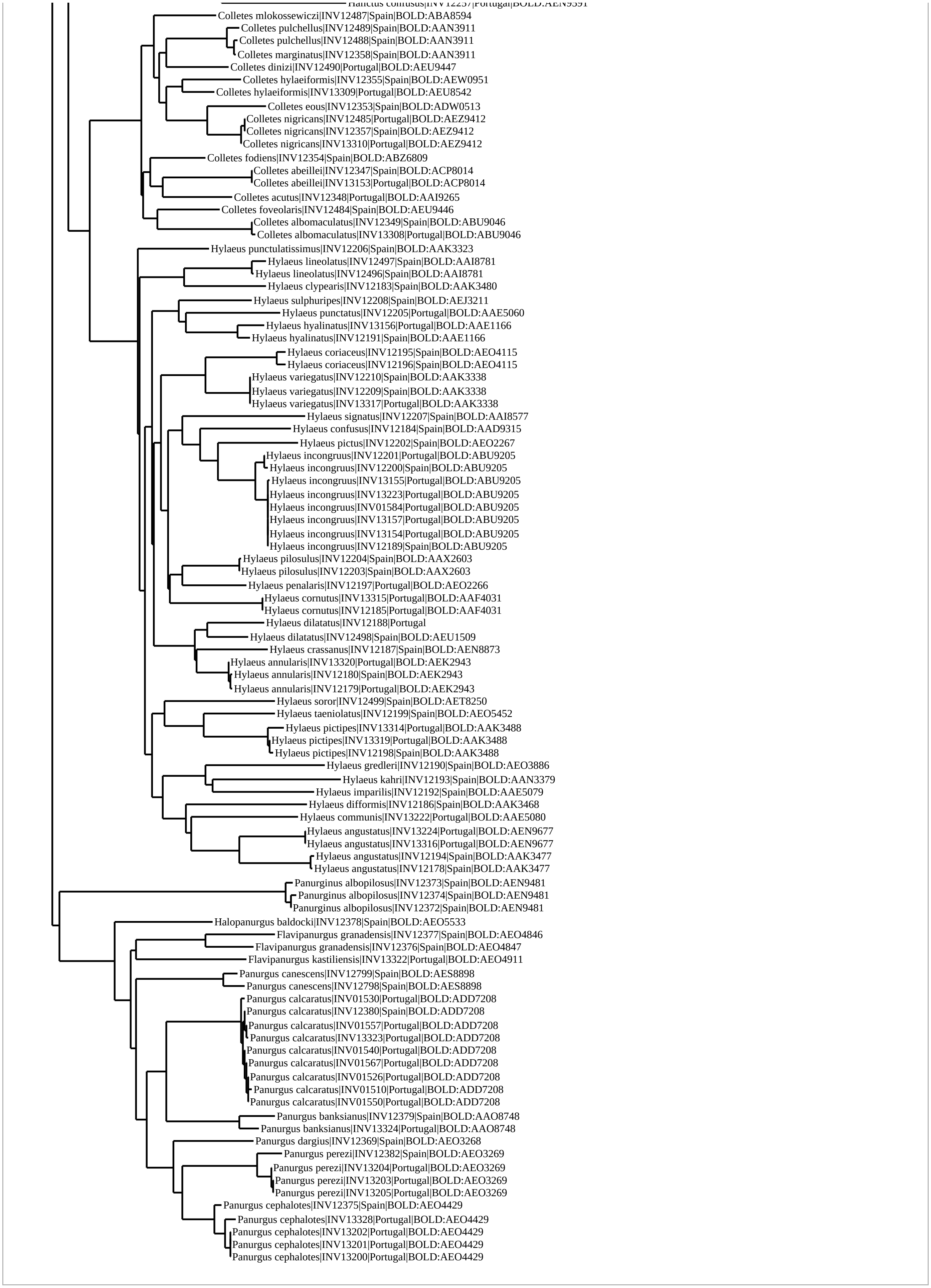
Halictus confusus|INV12257|Portugal|BOLD:AEN9591
Colletes mlokossewiczi|INV12487|Spain|BOLD:ABA8594
Colletes pulchellus|INV12489|Spain|BOLD:AAN3911
Colletes pulchellus|INV12488|Spain|BOLD:AAN3911
Colletes marginatus|INV12358|Spain|BOLD:AAN3911
Colletes dinizi|INV12490|Portugal|BOLD:AEU9447
Colletes hylaeiformis|INV12355|Spain|BOLD:AEW0951
Colletes hylaeiformis|INV13309|Portugal|BOLD:AEU8542
Colletes eous|INV12353|Spain|BOLD:ADW0513
Colletes nigricans|INV12485|Portugal|BOLD:AEZ9412
Colletes nigricans|INV12357|Spain|BOLD:AEZ9412
Colletes nigricans|INV13310|Portugal|BOLD:AEZ9412
Colletes fodiens|INV12354|Spain|BOLD:ABZ6809
Colletes abeillei|INV12347|Spain|BOLD:ACP8014
Colletes abeillei|INV13153|Portugal|BOLD:ACP8014
Colletes acutus|INV12348|Portugal|BOLD:AAI9265
Colletes foveolaris|INV12484|Spain|BOLD:AEU9446
Colletes albomaculatus|INV12349|Spain|BOLD:ABU9046
Colletes albomaculatus|INV13308|Portugal|BOLD:ABU9046
Hylaeus punctulatissimus|INV12206|Spain|BOLD:AAK3323
Hylaeus lineolatus|INV12497|Spain|BOLD:AAI8781
Hylaeus lineolatus|INV12496|Spain|BOLD:AAI8781
Hylaeus clypearis|INV12183|Spain|BOLD:AAK3480
Hylaeus sulphuripes|INV12208|Spain|BOLD:AEJ3211
Hylaeus punctatus|INV12205|Portugal|BOLD:AAE5060
Hylaeus hyalinatus|INV13156|Portugal|BOLD:AAE1166
Hylaeus hyalinatus|INV12191|Spain|BOLD:AAE1166
Hylaeus coriaceus|INV12195|Spain|BOLD:AEO4115
Hylaeus coriaceus|INV12196|Spain|BOLD:AEO4115
Hylaeus variegatus|INV12210|Spain|BOLD:AAK3338
Hylaeus variegatus|INV12209|Spain|BOLD:AAK3338
Hylaeus variegatus|INV13317|Portugal|BOLD:AAK3338
Hylaeus signatus|INV12207|Spain|BOLD:AAI8577
Hylaeus confusus|INV12184|Spain|BOLD:AAD9315
Hylaeus pictus|INV12202|Spain|BOLD:AEO2267
Hylaeus incongruus|INV12201|Portugal|BOLD:ABU9205
Hylaeus incongruus|INV12200|Spain|BOLD:ABU9205
Hylaeus incongruus|INV13155|Portugal|BOLD:ABU9205
Hylaeus incongruus|INV13223|Portugal|BOLD:ABU9205
Hylaeus incongruus|INV01584|Portugal|BOLD:ABU9205
Hylaeus incongruus|INV13157|Portugal|BOLD:ABU9205
Hylaeus incongruus|INV13154|Portugal|BOLD:ABU9205
Hylaeus incongruus|INV12189|Spain|BOLD:ABU9205
Hylaeus pilosulus|INV12204|Spain|BOLD:AAX2603
Hylaeus pilosulus|INV12203|Spain|BOLD:AAX2603
Hylaeus penalaris|INV12197|Portugal|BOLD:AEO2266
Hylaeus cornutus|INV13315|Portugal|BOLD:AAF4031
Hylaeus cornutus|INV12185|Portugal|BOLD:AAF4031
Hylaeus dilatatus|INV12188|Portugal
Hylaeus dilatatus|INV12498|Spain|BOLD:AEU1509
Hylaeus crassanus|INV12187|Spain|BOLD:AEN8873
Hylaeus annularis|INV13320|Portugal|BOLD:AEK2943
Hylaeus annularis|INV12180|Spain|BOLD:AEK2943
Hylaeus annularis|INV12179|Portugal|BOLD:AEK2943
Hylaeus soror|INV12499|Spain|BOLD:AET8250
Hylaeus taeniolatus|INV12199|Spain|BOLD:AEO5452
Hylaeus pictipes|INV13314|Portugal|BOLD:AAK3488
Hylaeus pictipes|INV13319|Portugal|BOLD:AAK3488
Hylaeus pictipes|INV12198|Spain|BOLD:AAK3488
Hylaeus gredleri|INV12190|Spain|BOLD:AEO3886
Hylaeus kahri|INV12193|Spain|BOLD:AAN3379
Hylaeus imparilis|INV12192|Spain|BOLD:AAE5079
Hylaeus difformis|INV12186|Spain|BOLD:AAK3468
Hylaeus communis|INV13222|Portugal|BOLD:AAE5080
Hylaeus angustatus|INV13224|Portugal|BOLD:AEN9677
Hylaeus angustatus|INV13316|Portugal|BOLD:AEN9677
Hylaeus angustatus|INV12194|Spain|BOLD:AAK3477
Hylaeus angustatus|INV12178|Spain|BOLD:AAK3477
Panurginus albopilosus|INV12373|Spain|BOLD:AEN9481
Panurginus albopilosus|INV12374|Spain|BOLD:AEN9481
Panurginus albopilosus|INV12372|Spain|BOLD:AEN9481
Halopanurgus baldocki|INV12378|Spain|BOLD:AEO5533
Flavipanurgus granadensis|INV12377|Spain|BOLD:AEO4846
Flavipanurgus granadensis|INV12376|Spain|BOLD:AEO4847
Flavipanurgus kastiliensis|INV13322|Portugal|BOLD:AEO4911
Panurgus canescens|INV12799|Spain|BOLD:AES8898
Panurgus canescens|INV12798|Spain|BOLD:AES8898
Panurgus calcaratus|INV01530|Portugal|BOLD:ADD7208
Panurgus calcaratus|INV12380|Spain|BOLD:ADD7208
Panurgus calcaratus|INV01557|Portugal|BOLD:ADD7208
Panurgus calcaratus|INV13323|Portugal|BOLD:ADD7208
Panurgus calcaratus|INV01540|Portugal|BOLD:ADD7208
Panurgus calcaratus|INV01567|Portugal|BOLD:ADD7208
Panurgus calcaratus|INV01526|Portugal|BOLD:ADD7208
Panurgus calcaratus|INV01510|Portugal|BOLD:ADD7208
Panurgus calcaratus|INV01550|Portugal|BOLD:ADD7208
Panurgus banksianus|INV12379|Spain|BOLD:AAO8748
Panurgus banksianus|INV13324|Portugal|BOLD:AAO8748
Panurgus dargius|INV12369|Spain|BOLD:AEO3268
Panurgus perezi|INV12382|Spain|BOLD:AEO3269
Panurgus perezi|INV13204|Portugal|BOLD:AEO3269
Panurgus perezi|INV13203|Portugal|BOLD:AEO3269
Panurgus perezi|INV13205|Portugal|BOLD:AEO3269
Panurgus cephalotes|INV12375|Spain|BOLD:AEO4429
Panurgus cephalotes|INV13328|Portugal|BOLD:AEO4429
Panurgus cephalotes|INV13202|Portugal|BOLD:AEO4429
Panurgus cephalotes|INV13201|Portugal|BOLD:AEO4429
Panurgus cephalotes|INV13200|Portugal|BOLD:AEO4429
